# Peri-weaning, diet-induced activation of an IFNγ-mediated regulatory circuit promotes cDC1 maturation and CD8^+^ T cell differentiation

**DOI:** 10.1038/s41467-026-75853-5

**Published:** 2026-08-04

**Authors:** Doğuş Altunöz, Ramin Shakiba, Kaushikk Ravi Rengarajan, Hamsa Narasimhan, Nikos E. Papaioannou, Sadiq Nasrah, Jessica Vetters, Maria L. Richter, María Parra Reyes, Nadine Nuschele, Denise Messerer, Sabine Schwamberger, Andreas Goschin, Dimitrios Starfas, Melanie Schmid, Tobias Straub, Michele Proietti, Katrin Böttcher, Maria Colomé-Tatché, Dirk Haller, Jan P. Böttcher, Stephanie C. Ganal-Vonarburg, Sophie Janssens, Christian Schulz, Anne B. Krug, Barbara U. Schraml

**Affiliations:** 1 Institute for Immunology, Biomedical Center, LMU Medizin, LMU Munich, Planegg-Martinsried, Germany; 2 Institute of Cardiovascular Physiology and Pathophysiology, Biomedical Center, LMU Medizin, LMU Munich, Planegg-Martinsried, Germany; 3 Laboratory for ER Stress and Inflammation, VIB-UGent Center for Inflammation Research, Ghent, Belgium; 4 Department of Internal Medicine and Pediatrics, Ghent University, Ghent, Belgium; 5 Physiological Chemistry, LMU Medizin, LMU Munich, Planegg-Martinsried, Germany; 6 Institute of Molecular Immunology, TUM University Hospital, Munich, Germany; 7 Department of Medicine I, LMU Klinikum, LMU Munich, Munich, Germany; 8 ZIEL Institute for Food & Health, TUM, Freising, Germany; 9 Institute for Immunodeficiency (IFI), Medical Center and Faculty of Medicine, University of Freiburg, Freiburg, Germany; 10 Department of Visceral Surgery and Medicine, Inselspital, Bern University Hospital, University of Bern, Bern, Switzerland; 11 Department for BioMedical Research, University of Bern, Bern, Switzerland; 12 Core Facility Bioinformatics, Biomedical Center, LMU Munich, Planegg-Martinsried, Germany; 13 Clinic for Immunology and Rheumatology, Hanover Medical School, Hanover, Germany; 14 RESiST-Cluster of Excellence 2155, Hanover Medical School, Hanover, Germany; 15 Department of Internal Medicine I, University Hospital Tübingen, Tübingen, Germany; 16 M3 Research Center, University Hospital Tübingen, University of Tübingen, Tübingen, Germany; 17 Chair of Nutrition and Immunology, School of Life Sciences, Technical, University of Munich, Freising, Germany; 18 Department of Experimental Immunology, Institute of Immunology, University of Tübingen, Tübingen, Germany; 19 Department of Immunopharmacology, Mannheim Institute for Innate Immunoscience (MI3), Medical Faculty Mannheim, University of Heidelberg, Mannheim, Germany; 20 Present Address: Laboratory of Immune Regulation, Center of Basic Sciences, Biomedical Research Foundation Academy of Athens, Athens, Greece

**Keywords:** Antigen-presenting cells, Conventional dendritic cells, Immune tolerance, Interferons

## Abstract

Maintaining a balanced immunity between pathogen defense and tolerance to environmental antigens in neonates is essential for survival and the establishment of life-long immune homeostasis. Instructed by environmental signals, type 1 conventional dendritic cells (cDC1) contribute to both processes but how the balance may be achieved is unclear. Here, we uncover an interferon (IFN)γ-driven regulatory circuit in early life that relays dietary cues to spleen cDC1. IFNγ-mediated STAT1-signaling induces an immunogenic maturation program in spleen cDC1 that enables them to shape the effector differentiation of antigen-experienced effector memory CD8⁺ T cells. This cDC1 program emerges during the transition from breastfeeding to solid food at weaning, occurs in germ-free mice, and remains operative to dietary intervention in adult mice. At weaning, this IFNγ signal enables spleen cDC1 to shape the effector phenotype of food-antigen-specific CD8^+^ T cells in a feedforward manner, thereby recalibrating the developing T cell pool. Our findings identify diet as a modifiable cue that can tune systemic cDC1-mediated immunity, opening new opportunities to steer immune responses during early life and beyond.

## Abstract (web summary)

Dendritic cells (DC) regulate CD8^+^ T cell differentiation to maintain a balance between pathogen protection and allergen tolerance, but how this balance may be enforced remain unclear. Here the authors show that weaning and the resulting food change modulate an IFNg-DC-CD8^+^ T cell circuit in infant mice to induce a switch in food antigen responses.

## Introduction

Early postnatal life is a critical period for developmental immune programming during which long-lasting immune phenotype and disease susceptibility are established^1–3^. During this time, specific regulatory circuits ensure tolerogenic immune responses against environmental antigens, such as food and commensal microbes, that can suppress inflammatory events even in later life^4–6^. Simultaneously, immune cells are programmed with effector functions that are beneficial for anti-pathogen defense but can be harmful if directed against environmental antigens^7,8^. Understanding these immunostimulatory circuits holds great promise for treating allergy, improving vaccine efficacy or boosting immunity in diseases characterized by immunosuppression, such as cancer^1^.

Conventional dendritic cells (cDCs) are potent orchestrators of T cell-mediated immunity^9,10^. Depending on the context in which they encounter antigen, such as the presence or absence of infection, inflammation, or tissue damage, cDCs can drive immunogenic or tolerogenic T cell responses^9,10^. cDCs exist as distinct subtypes with specific functions in pathogen defense. Type 1 conventional dendritic cells (cDC1) are potent activators of CD4^+^ T helper (Th) 1 and cytotoxic T cell responses against intracellular pathogens^9–12^. cDC2 on the other hand preferentially promote Th2 and Th17 responses against extracellular pathogens, as well as T follicular helper cell differentiation^9,10,13–16^. In addition to these well-defined roles in anti-pathogen defense, both cDC1 and cDC2 can adopt functional states that promote T cell tolerance or activation in response to environmental cues that are often tissue specific^6,17–20^.

One mechanism through which cDCs acquire functional states is a process known as maturation^9,21,22^. Upon recognition of pathogens via pattern recognition receptors (PRRs), both cDC types upregulate expression of antigen-presentation machinery, costimulatory molecules and C–C chemokine receptor type 7 (CCR7)—a process called “immunogenic” maturation in reference to stimulation by pathogenic signals^9,21,22^. CCR7-expressing cDCs can migrate to T cell areas of secondary lymphoid organs, where they subsequently initiate T cell responses^9,21,23^. The maturation into CCR7^+^ cDCs also takes place in steady-state in the absence of microbial signals and in germ-free conditions and is thus referred to as “homeostatic” maturation^21,24^. Homeostatic cDC maturation is thought to primarily promote T cell tolerance^21^. In cDC1, for instance, the homeostatic maturation into CCR7-expressing cells can be induced by uptake of apoptotic cells and subsequent LXRβ-dependent cholesterol efflux^25^. Loss of LXRβ signaling in cDC1 causes increased activation of B and T cells in steady state adult mice^25^. In the thymus of adult mice, thymic epithelial cells produce type I and III IFNs (IFN-I and IFN-III) that promote the maturation of cDC1 into CCR7^+^ cells^26^. Whether this IFN-induced maturation involves the recognition of apoptotic cells is unclear, but it occurs independent of microbial signals and is required for the thymic selection of regulatory T cells^26^. Thus, in homeostatic conditions, cDCs integrate tissue-derived signals, including apoptotic cell death and cytokines, to promote T cell tolerance.

Early life is a period of substantial immune challenge during which the immune system must continuously interpret and respond to a rapidly changing environment. The most profound changes to the external environment are dietary changes during weaning and the initial encounter with commensals and pathogenic microbes^27,28^. Although early-life cDCs were once considered functionally immature to accommodate these challenges, accumulating evidence suggests they are instead finely tuned to their developmental context, allowing them to establish and maintain homeostasis^3,6,29–33^. Apoptotic cell-driven cDC1 maturation is thought to suppress T cell responses to dying self during lung remodeling in neonatal mice^31^. In the pre-weaning period compared to adulthood, cDC2 exhibit a heightened ability to promote peripheral Treg differentiation in murine skin, lung, and spleen^6,29,30^. In neonatal skin, cDC2 induce commensal-specific Tregs during a defined developmental window that prevent inflammation in later life^5,6^. The signals that drive the tolerogenic potential of cDC2 in neonatal skin and spleen are undefined, but in neonatal lung, commensal encounter triggers a tolerogenic program in cDC2^29^. On the contrary, early microbial colonization enables spleen cDC1 to elicit Th1 responses to immunization with Ovalbumin (OVA) and protective CD8⁺ T cell responses to *Listeria monocytogenes*, suggesting the induction of immunogenic programs by commensal encounter^32,33^. Similarly, in adult mice, commensal microbes drive IFN-I production from plasmacytoid DCs (pDCs) that transcriptionally poises splenic cDCs with inflammatory capacity^34^. Of note, transcriptional analyses show higher IFN-induced genes in spleen cDC2 of adult compared to young mice, but lack of commensals does not cause cDC2 from adult mice to acquire a phenotype resembling cDC2 from young mice^30^. Thus, the functions of cDCs appear to be shaped by commensal microbiota in a tissue and age-specific manner.

Underscoring the influence of tissue-context on early-life cDC functions, neonatal cDC2 exhibit a Th2 bias in the lung, but not spleen, that is driven by type 2 cytokines during lung alveolarization^30,35^. In neonatal spleen, both cDC1 and cDC2 are functionally competent to induce inflammatory T cell responses^30,32,36^, while in murine Peyer’s patches, cDCs acquire immunocompetency only after weaning^37^. cDC immunocompetency at this site is reached independent of the microbiota and follows the differentiation of M cells within the follicle-associated epithelium^37^. Thus, cDCs do not reach immunocompetency in all tissues simultaneously, suggesting tight regulation of cDC function across developmental age that is coordinated by tissue-specific signals and external factors, such as commensals.

Here, we set out to define regulatory circuits that determine cDC1 function in the spleen during early life and weaning. We find that the dietary switch at weaning triggers a Myd88/TRIF-dependent immune response culminating in IFNγ production from lymphocytes. In the spleen, IFNγ pushes cDC1 towards an immunostimulatory cell state characterised by CXCL9 expression and distinct from the previously described CCR7⁺ homeostatic maturation program. At weaning, this diet-responsive IFNγ signal relays nutritional cues to splenic cDC1, enabling them to shape the effector phenotype of food antigen–specific CD8⁺ T cells at distal sites. Thus, homeostatic maturation of cDC1 is not simply associated with T cell tolerance, but can also promote the acquisition of effector function by antigen-specific CD8⁺ T cells. Moreover, cDC1 maturation remains responsive to dietary intervention in adult mice. These findings reveal diet as an instructive regulator of systemic cDC function, suggesting that dietary interventions could be harnessed to modulate cDC-mediated immunity in vaccination, immunotherapy, and immune-mediated disease.

## Results

### cDC1 from spleens of young and adult mice exhibit functional and transcriptional differences

**Fig. 1:**
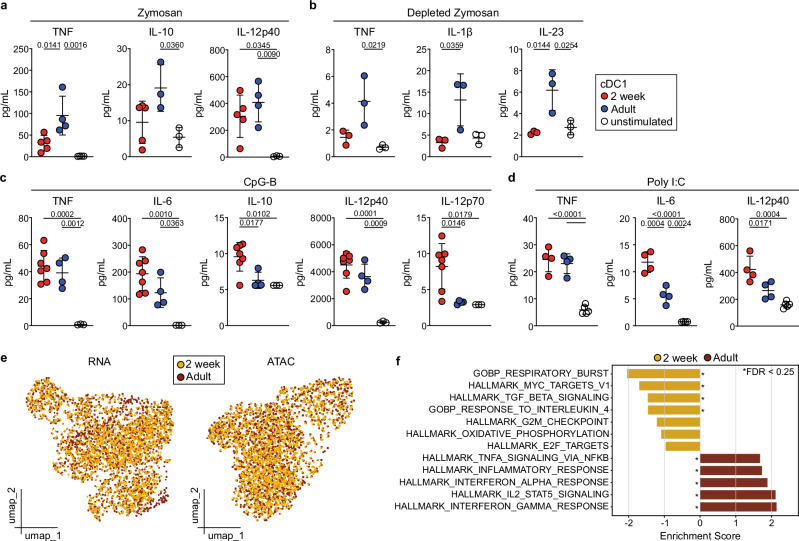
cDC1 from spleens of young and adult mice exhibit functional and transcriptional differences. **a–d** Splenic cDC1 from two or 7–14-week-old adult mice were left untreated or stimulated with **a** Zymosan (2 week (*n* = 5); Adult or unstimulated (*n* = 4)), **b** Depleted Zymosan (*n* = 3), **c** CpG-B (2 week (*n* = 7); Adult (*n* = 4); unstimulated (*n* = 3) or **d** Poly I:C (*n* = 4; unstimulated (*n* = 5)). After 20 h, cytokines were quantified in supernatants. Unstimulated data points are mixed from 2-week-old and adult mice. Dots represent biological replicates. Data are pooled from two independent experiments (**a**, **c**) or representative of two independent experiments (**b**, **d**). Horizontal bars represent mean, error bars represent SD. *p*-values were determined using one-way ANOVA with two-tailed Tukey’s multiple comparisons test. Only statistically significant comparisons are indicated. **e**, **f** Cells annotated as cDC1 from 2-week-old and adult mice were isolated from a published dataset^40^. **e** RNA-based and ATAC-based UMAP with cells colored by age. **f** GSEApy was run with default parameters and enrichment scores of some gene sets differentially regulated between cDC1 from 2-week-old and adult mice are shown. Source data are provided as a Source Data file.

We first confirmed that cDC1 from spleens of young and adult mice show distinct responsiveness to stimulation with pathogen-associated molecular patterns (PAMPs)^38,39^. We sort-purified XCR1^+^ cDC1 from spleens of 2-week-old and adult mice and measured cytokine production after stimulation with different PRR agonists (Fig. 1a–d and Supplementary Fig. 1a, b). cDC1 from adult mice stimulated with the Toll-like-receptor (TLR) 2 and Dectin-1 ligand Zymosan, showed higher TNF production than those from 2-week-old mice (Fig. 1a). Similarly, depleted Zymosan, an exclusive Dectin-1 ligand, induced higher TNF, IL-23, and IL-1β production in cDC1 from adult compared to 2-week-old mice (Fig. 1b). In contrast, cDC1 from 2-week-old mice showed higher production of IL-10 and IL-12p70, the active form of IL-12, in response to CpG-B stimulation (Fig. 1c). Similarly, poly I:C stimulation induced stronger production of IL-12p40 and IL-6 in cDC1 from 2-week-old compared to adult mice (Fig. 1d). TNF production in response to CpG-B and poly I:C was similar across age (Fig. 1c, d). Thus, cDC1 from neonates and adult mice show qualitative differences in cytokine production after PRR stimulation as expected^39^.

Analysis of a single cell RNA and ATAC sequencing dataset of splenic cDCs from 2-week-old and adult mice^40^ revealed cDC1 as transcriptionally similar across age, with low but similar expression of *Tlr2, Tlr3*, and *Tlr9* transcripts in young and adult mice (Fig. 1e and Supplementary Fig. 1c). Gene set enrichment analysis (GSEA), however, identified several pathways in XCR1^+^ cDC1 differentially regulated by age (Fig. 1f). cDC1 from adult mice were enriched for genes downstream of IFN-I and IFNγ (Fig. 1f), which is in line with our prior observations that spleen cDC2 from adult versus 2-week-old mice show enrichment of IFN-stimulated genes^30,40^.

### Spleen cDC1 transcriptionally change during weaning

**Fig. 2:**
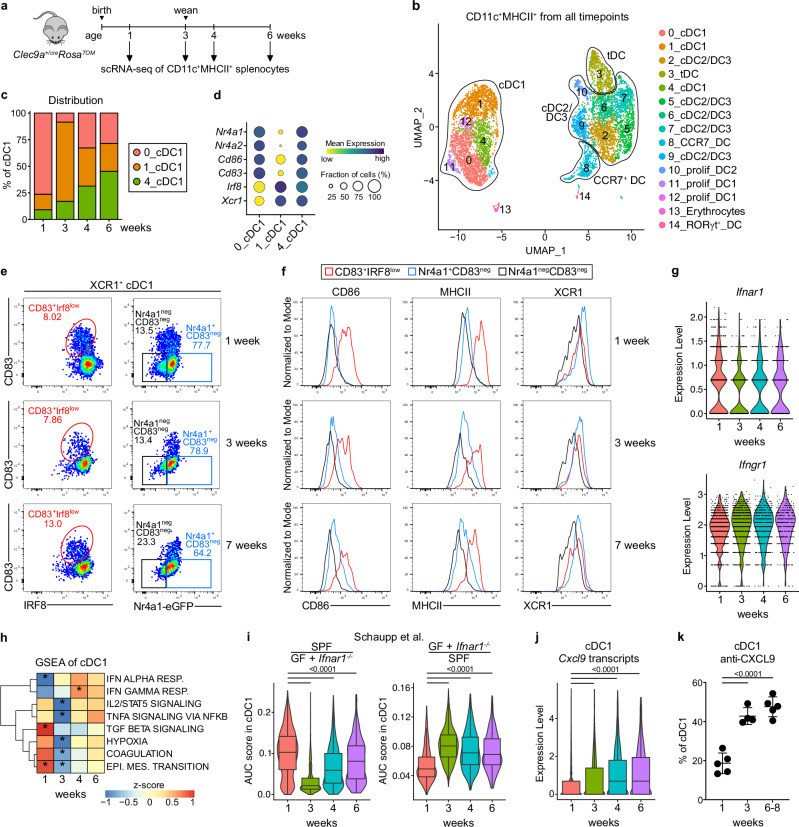
Spleen cDC1 transcriptionally change around weaning. **a** CD11c^+^MHCII^+^ cells were sorted from spleens of mice at the indicated ages and subjected to scRNA-seq. **b** UMAP display of 7689 CD11c^+^MHCII^+^ cells grouped from all ages and annotated by cell type. **c** Relative frequency of cDC1 clusters 0, 1, and 4 at the different time points. **d** Bubble plot displaying expression of indicated genes across cDC1 clusters 0, 1, and 4. **e** Flow cytometry of splenic XCR1^+^ cDC1 from *Nr4a1*^*eGFP*^ mice of indicated ages (gating strategy in Supplementary Fig. 2h). XCR1^+^ cDC1 were divided into CD83^+^IRF8^low^ (red), GFP^+^CD83^neg^ (blue) and GFP^neg^CD83^neg^ (black) populations. **f** Histograms of the indicated surface markers on CD83^+^IRF8^low^ (red), GFP^+^CD83^neg^ (blue) and GFP^neg^CD83^neg^ (black) cDC1. **g–j** cDC1 clusters 0, 1, and 4 were grouped for analyses. **g** Expression levels of *Ifnar1* and *Ifngr1* in cDC1 across age. **h** Gene set enrichment analysis (GSEA) of cDC1 across age. Only gene sets with *p* < 0.01(*) for at least one time point are shown. **i**, **j** AUC scores for genes described to be regulated by IFNAR in cDCs^34^ (**i**) and *Cxcl9* (**j**) expression in cDC1 clusters (0, 1, 4) across age. Boxes represent interquartile range, horizontal bars represent median, whiskers represent minimum and maximum (**i**, **j**). **k** CXCL9 expression in splenic XCR1^+^ cDC1 quantified by flow cytometry at the indicated ages (1 and 6–8 weeks (*n* = 5); 3 weeks (*n* = 4)). Dots represent biological replicates from two independent experiments. Horizontal bars represent mean, error bars represent SD. Statistical analysis was performed using two-tailed variance-inflated Wilcoxon rank sum test with adjustment for multiple comparisons via the FDR method (**h**), two-tailed Wilcoxon ranked sum test with Benjamini–Hochberg adjustment for multiple comparisons (**i**, **j**) or one-way ANOVA with two-tailed Tukey’s multiple comparisons test (**k**). Only statistically significant comparisons are indicated. Source data are provided as a Source Data file.

Because commensal-induced IFNs transcriptionally shape spleen cDCs^34,41^ and weaning the transition from breast milk to chow correlates with a strong increase in microbial diversity^4^, we hypothesized that weaning might be a critical period in the functional regulation of splenic cDC1. To address this hypothesis, we performed single-cell RNA-sequencing (scRNA-seq) of CD11c^+^MHCII^+^ splenocytes from mice aged 1, 3, 4, and 6 weeks (Fig. 2a and Supplementary Fig. 2a). All mice were separated from their mothers at exactly three weeks of age and time points were chosen to cover the perinatal period around weaning until shortly before the onset of sexual maturity. After quality control, we retained gene expression profiles from 7689 cells with similar contribution from each time point (week 1: 2131 cells, week 3: 2050 cells, week 4: 1752 cells, week 6: 1756 cells). Unsupervised graph-based clustering of cells from all time points resulted in 15 clusters that segregated into two main metaclusters: cDC1 (clusters 0, 1, 4, 11, 12)^42^ and a cluster containing cDC2/DC3 (clusters 2, 5, 6, 7, 9, 10)^42–44^, transitional DCs (tDCs; cluster 3)^45^ and CCR7^+^ migratory cDC (cluster 8)^46^ (Fig. 2b, Supplementary Fig. 2b–f, and Supplementary Data 1). Cluster 14 could be identified as RORγt^+^ DCs^30,40^ (Supplementary Fig. 2d, f), whereas cluster 13 was likely a contamination with *Gypa* expressing erythrocytes^47^ (Supplementary Fig. 2d). Although we regressed cell cycle genes as cDCs proliferate in the developing spleen before weaning^30^, we found that clusters 10, 11, and 12 mainly consisted of proliferating cells (Supplementary Fig. 2b, c). Homeostatically matured *Ccr7*^+^ cDC1 downregulate XCR1^24,25,48^, and in line with this, we observed low expression of *Xcr1* in *Ccr7*^+^ cDCs (Supplementary Fig. 2d). Of note, all cDC clusters contained cells from each time point, demonstrating that cDCs preserve their overall identity independent of developmental age (Supplementary Fig. 2g).

Since we had shown that XCR1^+^ cDC1s exhibit age-dependent differences in cytokine production after PRR simulation (Fig. 1), we focused on *Xcr1*^+^ cDC1 clusters 0, 1, and 4 for further analyses. The abundance of clusters 0, 1, and 4 within cDC1 changed with age and stabilized by 4 weeks of age (Fig. 2c). Cluster 0 showed lower expression of *Irf8* and *Xcr1* compared to clusters 1 and 4, while cluster 1 expressed lowest levels of *Nr4a1, Nr4a2, Cd86*, and *Cd83* (Fig. 2d and Supplementary Data 2–7), suggesting the clusters could be distinct maturation states. To assess if this transcriptional heterogeneity could be confirmed by protein staining, we performed flow cytometry in the spleens of *Nr4a1*^*eGFP*^ reporter mice. Indeed, XCR1^+^ cDC1 could be divided into CD83^+^GFP^+^, CD83^neg^GFP^+^, and CD83^neg^GFP^neg^ cells (Fig. 2e and Supplementary Fig. 2h). Transcriptionally CD83^+^ cells appeared more abundant than detected by flow cytometry, possibly owing to post-transcriptional regulation^49^. CD83^neg^GFP^neg^ cells showed lowest levels of CD86 and MHCII and most closely resembled cluster 1, while CD83^neg^GFP^+^ cells expressed intermediate levels of CD86 and MHCII and most closely resembled cluster 4 (Fig. 2d–f). The CD83^+^XCR1^+^ population most closely resembled cluster 0 with relatively low levels of IRF8 and highest levels of CD86 and MHCII at all ages (Fig. 2d–f). Thus, cDC1 exist as distinct maturation states, with CD83^+^ cells representing a more mature state of cDC1 than CD83^neg^ cells.

To gain insights into specific pathways that could regulate cDC1 maturation around weaning, we performed GSEA (Fig. 2h). *Xcr1*^+^ cDC1 from 1-week-old mice showed lower expression of genes downstream of IFNα and IFNγ compared to *Xcr1*^+^ cDC1 from all other time points, although expression of *Ifnar1* and *Ifngr1* was similar across age (Fig. 2g, h). Accordingly, we found lower expression of genes induced by microbiota in an interferon-alpha/beta receptor (IFNAR) dependent manner^34^ and conversely higher expression of genes suppressed by IFNAR signaling^34^ in cDC1 from one compared to 3-week-old mice (Fig. 2i). Additionally, we found that the expression of the IFN-stimulated gene *Cxcl9*^50,51^ increased in cDC1 between one and 3 weeks of age, which was confirmed by protein staining (Fig. 2j, k). Together, these results identify IFN signaling as a potential driver of age-dependent functional programs in splenic cDC1 around weaning.

### IFNγ production from spleen lymphocytes rises with age

**Fig. 3:**
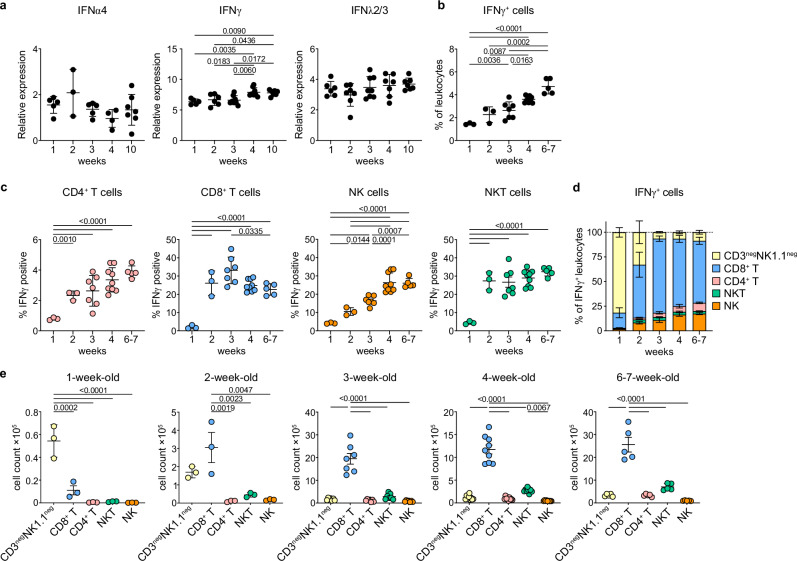
Splenic IFNγ levels rise with age, while IFN-I and IFN-III remain constant. **a** Quantification of *Ifna4*, *Ifng* and *Ifnl2/3* expression in total spleen tissue by quantitative real-time PCR (qRT-PCR). Expression relative to *Actb* is shown (*Ifna4*: 1 or 3 weeks (*n* = 5); 2 weeks (*n* = 3); 4 weeks (*n* = 4); 10 weeks (*n* = 7); *Ifng*: 1 week (*n* = 5); 2 weeks (*n* = 6); 3 or 4 weeks (*n* = 8); 10 weeks (*n* = 7); *Ifnl2/3*: 1 week (*n* = 6); 2, 4, or 10 weeks (*n* = 7); 3 weeks (*n* = 8)). **b–e** Splenocytes from mice at the indicated ages were cultured for 5 h with phorbol 12-myristate 13-acetate (PMA) and ionomycin. Brefeldin A was added for the last 3 h. Cells were then stained for surface markers and IFNγ and analyzed by flow cytometry. **b** Frequency of IFNγ-producing leukocytes across age. **c** Frequency of IFNγ-producing CD4^+^, CD8^+^, NK, and NKT cells at the indicated ages. **d** Contribution of different lymphocyte subsets to total IFNγ^+^ cells. **e** Cell numbers of IFNγ-producing leukocytes across age. **b–e** 1 or 2 weeks (*n* = 3); 3 weeks (*n* = 7); 4 weeks (*n* = 9); 6–7-weeks (*n* = 5). Dots represent biological replicates pooled from at least two independent experiments, horizontal bars represent mean, error bars represent SD. Numbers indicate *p*-values determined by one-way ANOVA with two-tailed Tukey’s multiple comparisons test. Only statistically significant comparisons are indicated. Source data are provided as a Source Data file.

IFN-I, IFN-II, and IFN-III stimulate the expression of similar genes. To start to address which type of IFN could be imprinting cDC1 with age, we profiled the expression of IFN-I (IFNα, IFNβ), IFN-II (IFNγ), and IFN-III (IFNλ2/3) in the spleen using quantitative real time PCR (qRT-PCR; Fig. 3a). We observed that the expression of *Ifna4* and *Ifnl2/3* was constant in spleens of mice across age (1, 2, 3, 4, and 10 weeks; Fig. 3a). In contrast, expression of *Ifng* increased steadily from one to 4 weeks of age and somewhat declined thereafter (Fig. 3a). Accordingly, we observed that the frequency of IFNγ producing lymphocytes in spleen increased steadily from one week to 7 weeks of age (Fig. 3b and Supplementary Fig. 3a). IFNγ production increased with age in NK and NKT cells, as well as in CD4^+^ T cells (Fig. 3c) but spiked at week three in CD8^+^ T cells (Fig. 3c). Notably, CD8^+^ T cells constituted the biggest fraction of IFNγ-producing cells at all time points except one week of age, when most IFNγ was produced by CD3^neg^NK1.1^neg^ cells (Fig. 3d, e).

### IFNγ mediates STAT1-dependent CXCL9 production in cDC1 independent of the microbiota

**Fig. 4:**
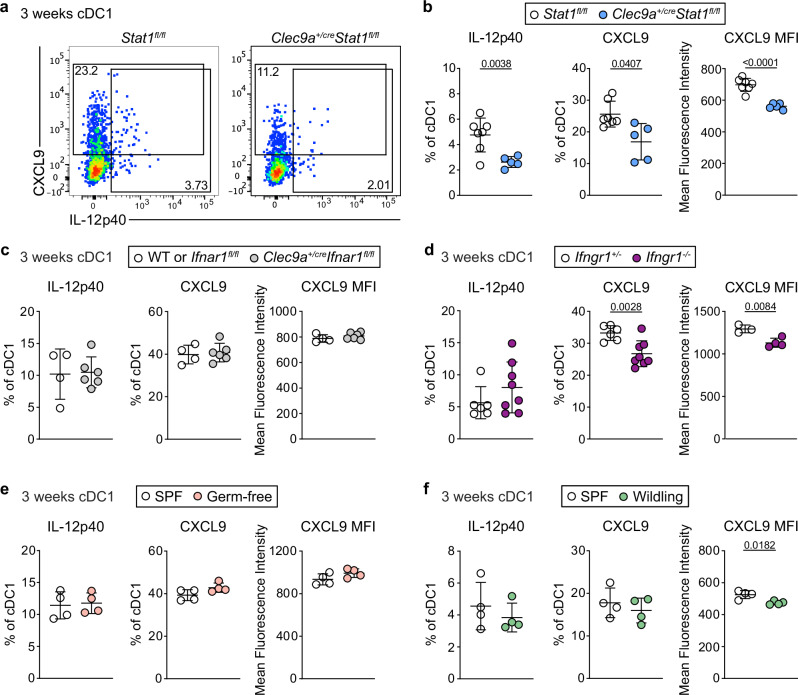
IFNγ mediates STAT1-dependent CXCL9 production in cDC1 independent of the microbiota. **a–f** Splenocytes from 3-week-old mice of the indicated genotypes were cultured for 4 h with Brefeldin A and Monensin. XCR1^+^ cDC1 were then analyzed for cytokine production by flow cytometry. **a** CXCL9 and IL-12p40 staining profiles and **b** quantification in cDC1 from *Clec9a*^*+/cre*^*Stat1*^*fl/fl*^ (*n* = 5) and *Stat1*^*fl/fl*^ (*n* = 7) littermates. **c** Quantification of IL-12p40^+^ and CXCL9^+^ cDC1 from 3-week-old *Clec9a*^*+/cre*^*Ifnar1*^*fl/fl*^ (*n* = 6) and *Ifnar1*^*fl/fl*^ littermates or age-matched wild type controls (*n* = 4). **d** Quantification of IL-12p40^+^ and CXCL9^+^ cDC1 from 3-week-old and *Ifngr1*^*+/−*^ (*n* = 6; for MFI *n* = 3) or *Ifngr1*^*−/−*^ mice (*n* = 8; for MFI *n* = 4). **e**, **f** cDC1 from 3-week-old germ-free (GF) and age-matched specific pathogen-free (SPF) mice (*n* = 4) (**e**) or 3-week-old wildling and age-matched SPF mice (*n* = 4) (**f**) were analyzed as above and IL-12p40^+^ and CXCL9^+^ cDC1 were quantified. Each dot represents one biological replicate pooled from two independent experiments (**a–d**). Data in (**e**, **f**) are from one experiment. Horizontal bars represent mean, error bars represent SD. Statistical analysis was performed and *p*-values were determined using two-tailed Welch’s *t*-test. Only statistically significant comparisons are indicated. Source data are provided as a Source Data file.

Signaling downstream of all types of IFN is mediated by the transcription factor signal transducer and activator of transcription 1 (STAT1)^52,53^. We therefore crossed *Stat1*^*flox*^ mice to *Itgax*^*cre*^ and *Clec9a*^*cre*^ mice^54,55^ to abrogate IFN-responsiveness specifically in cDCs. At 3 weeks of age, when we had first observed IFN-induced gene expression (Fig. 2h), we profiled cDC1 from both *Itgax*^*cre*^*Stat1*^*fl/fl*^ and *Clec9a*^*+/cre*^*Stat1*^*fl/fl*^ mice for CXCL9 production. CXCL9 was reduced in both the frequency and the amount of CXCL9 produced per cell in cDC1 from *Itgax*^*cre*^*Stat1*^*fl/fl*^ and *Clec9a*^*+/cre*^*Stat1*^*fl/fl*^ compared to control mice; however, CXCL9 production was not completely abolished (Fig. 4a, b and Supplementary Fig. 4a, b). Of note, STAT1-deficient cDC1 also showed reduced production of IL-12p40 (Fig. 4a, b and Supplementary Fig. 4a, b), a marker of homeostatic cDC maturation^56^. Interestingly, we observed no differences in CXCL9 or IL-12p40 production from cDC1 from 3-week-old mice with constitutive deletion of *Ifnar1* (Supplementary Fig. 4c) or with deletion of *Ifnar1* specifically in cDCs (*Clec9a*^*+/cre*^*Ifnar1*^*fl/fl*^ mice, Fig. 4c). In contrast, cDC1 from 3-week-old mice lacking IFNγ receptor (*Ifngr1*^*−/−*^) showed reduced CXCL9 production compared to age-matched *Ifngr1*^*+/−*^ controls, whereas IL-12p40 production was similar between genotypes (Fig. 4d). Of note, among cDC subsets, cDC1 were the dominant producers of CXCL9, while production from cDC2 or pDCs was negligible (Supplementary Fig. 4d). Thus, CXCL9 production in cDC1 from weanling mice is regulated by IFNγ in a STAT1-dependent manner.

Weaning demarcates the change from breastfeeding to grain-based chow diet in mice, which correlates with a strong increase in commensal microbial diversity^4^. Since commensal microbiota have been linked to IFN-I and IFNγ-mediated signaling pathways in spleen immune cells^34,41^, we assessed CXCL9 production from cDC1 in germ-free (GF) and wildling mice at weaning. GF mice are raised sterile and are not colonized by commensals^57^, while “wildling” mice harbor a highly diverse microbiome resembling that of mice in their natural habitat^58^. Notably, we observed no differences in CXCL9 or IL-12p40 production between cDC1 from age-matched mice housed under specific pathogen-free (SPF) and GF conditions (Fig. 4e) or between wildling and SPF mice (Fig. 4f).

### STAT1-signaling in cDC1 affects the homeostasis of CD8^+^ T cells in murine spleen

**Fig. 5:**
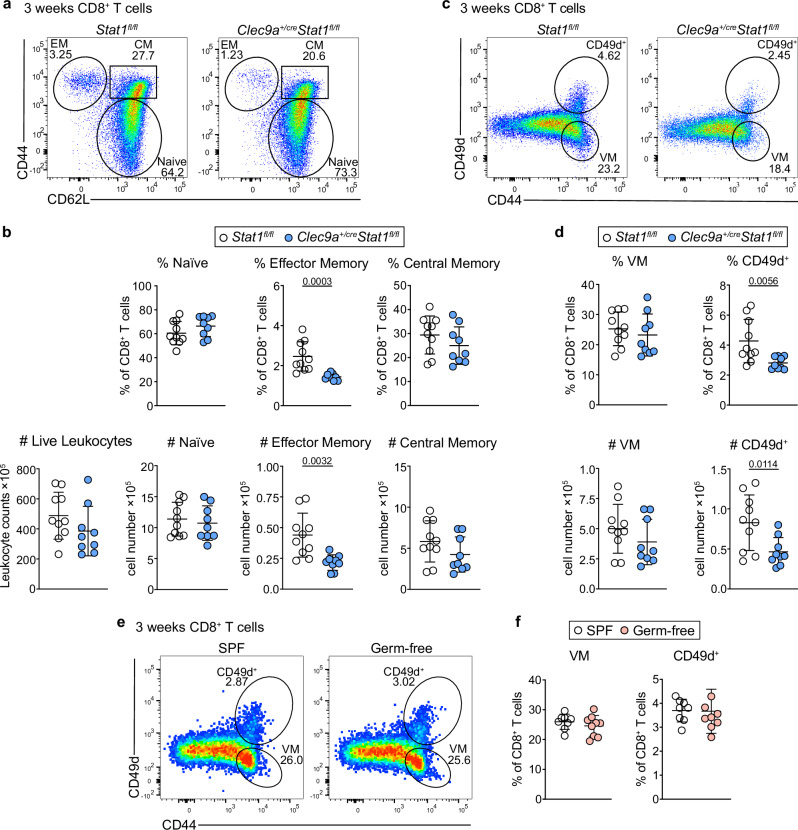
STAT1-signaling in cDC1 affects the homeostasis of CD8^+^ T cells in murine spleen. **a**, **b** CD8^+^ T cells in the spleen were divided into effector memory (CD44^+^CD62L^neg^), central memory (CD44^+^CD62L^+^) and naïve (CD44^neg^CD62L^+^) populations. **a** Representative gating and **b** quantification of CD8^+^ T cell subsets in spleens of 3-week-old *Clec9a*^*+/cre*^*Stat1*^*fl/fl*^ (*n* = 9) and *Stat1*^*fl/fl*^ (*n* = 10) littermates. **c**, **d** CD8^+^ T cells in the spleen were divided into CD49d^+^CD44^+^ antigen-experienced and CD49d^neg^CD44^+^ virtual memory (VM) populations. Gating (**c**) and quantification (**d**) of CD49d^+^ and VM populations in 3-week-old *Clec9a*^*+/cre*^*Stat1*^*fl/fl*^ (*n* = 9) and *Stat1*^*fl/fl*^ littermates (*n* = 10). **e**, **f** Representative gating (**e**) and quantification (**f**) of CD49d^+^ and VM populations in 3-week-old germ-free (GF, *n* = 9) and age-matched specific pathogen-free (SPF) mice (*n* = 8). Each dot represents one biological replicate pooled from two independent experiments, horizontal bars represent mean, error bars represent SD. *p*-values determined using two-tailed Welch’s *t*-test are indicated for statistically significant comparisons. Source data are provided as a Source Data file.

The above data show that IFNγ induces CXCL9 production in a fraction of cDC1 independent of microbial stimuli, indicating that this constitutes a homeostatic maturation process. Since homeostatic cDC maturation promotes T cell tolerance^21,24–26^, we profiled splenic T cells in *Itgax*^*cre*^*Stat1*^*fl/fl*^ and *Clec9a*^*+/cre*^*Stat1*^*fl/fl*^ mice at 3 weeks of age. CD4^+^ and CD8^+^ T cells were distinguished into cells with a naïve (CD44^neg^CD62L^+^), central memory (T_CM_; CD44^+^CD62L^+^) and effector memory (T_EM_; CD44^+^CD62L^neg^) phenotype (Fig. 5a, b and Supplementary Fig. 5a–c). Additionally, we profiled Foxp3^+^ regulatory T cells. We found no consistent differences between genotypes in CD4^+^ T_EM_ or T_CM_ cells, as well as FOXP3^+^ Tregs (Supplementary Fig. 5a–f). In contrast, CD8^+^ T cells in *Itgax*^*cre*^*Stat1*^*fl/fl*^ and *Clec9a*^*+/cre*^*Stat1*^*fl/fl*^ mice showed a specific reduction of T_EM_ phenotype cells compared to age-matched controls (Fig. 5a, b and Supplementary Fig. 5g, h). CD8^+^ T cells with a T_CM_ phenotype were similar between genotypes (Fig. 5a, b and Supplementary Fig. 5g, h).

Although memory CD8^+^ T cells typically arise in an antigen-dependent manner in response to infection, memory-phenotype CD8^+^ T cells also exist in substantial numbers within hosts that have not been exposed to pathogens^59–61^. These memory-phenotype T cells entail so called “innate memory” or “virtual memory” T cells that are antigen-inexperienced and are propagated in the periphery by IL-15 and IL-4 produced by cDC1s^61–63^. They can be distinguished from antigen-experienced T cells by lack of integrin α4 (CD49d)^60,61,64,65^. How these antigen-experienced memory phenotype T cells arise in hosts that have not been exposed to pathogens and what antigens they recognize is unclear. We found a specific reduction of CD49d^+^CD44^+^CD8^+^ T cells in 3-week-old *Itgax*^*cre*^*Stat1*^*fl/fl*^ and *Clec9a*^*+/cre*^*Stat1*^*fl/fl*^ compared to control mice, whereas CD49d^neg^CD44^+^CD8^+^ virtual memory T cells were similar between genotypes (Fig. 5c, d and Supplementary Fig. 5i). Of note, CD49d^+^ CD8^+^ T cells included cells with a T_EM_ and T_CM_ phenotype, however, only cells with a T_EM_ phenotype were reduced in *Clec9a*^*+/cre*^*Stat1*^*fl/fl*^ compared to control mice (Supplementary Fig. 5j, k). At 3 weeks of age these CD49d^+^CD44^+^ T_EM_ cells contained only few MR1-5-OP-RU tetramer-positive circulating Mucosal-Associated Invariant T (MAIT) cells, which also express CD8α, CD44 and CD49d and can be found in the mouse spleen^66^ (Supplementary Fig. 5l, m). Furthermore, the frequency of CD49d^+^CD44^+^CD8^+^ T_EM_ cells was similar between SPF and GF mice (Fig. 5e, f), further supporting that these cells are not MAIT cells, which are absent in GF mice^67^. Thus, antigen-experienced CD8^+^ T cells with T_EM_ phenotype that arise independent of microbial exposure are reduced in the spleen during weaning in mice with cDC-intrinsic loss of *Stat1*.

### STAT1-signaling induces a specific immunostimulatory state of cDC1

**Fig. 6:**
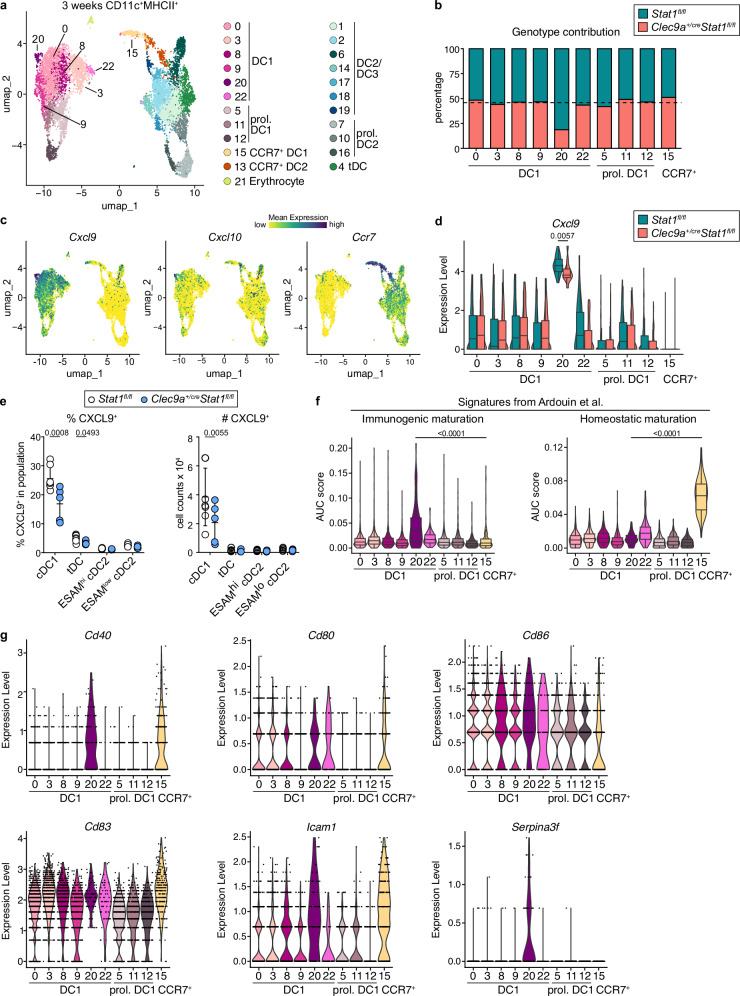
STAT1-signaling is required for an immunostimulatory state of cDC1. **a–d** scRNA-seq of CD11c^+^MHCII^+^ cells from spleens of 3-week-old *Clec9a*^*+/cre*^*Stat1*^*fl/fl*^ and *Stat1*^*fl/fl*^ mice. **a** UMAP display of 9906 CD11c^+^MHCII^+^ cells from both genotypes annotated by cell type (*Clec9a*^*+/cre*^*Stat1*^*fl/fl*^: 4935 cells, *Stat1*^*fl/fl*^: 4971 cells). **b** Contribution of cells from each genotype to individual cDC1 clusters. Dotted line is the contribution of cDC1 of *Clec9a*^*+/cre*^*Stat1*^*fl/fl*^ genotype to all cDC1 clusters combined. **c** Expression of *Cxcl9, Cxcl10*, and *Ccr7* projected on the UMAP display independent of genotype. **d** Expression levels of *Cxcl9* in cDC1 clusters split by genotype. **e** cDC subsets from 3-week-old *Stat1*^*fl/fl*^ (*n* = 7) and *Clec9a*^*+/cre*^*Stat1*^*fl/fl*^ (*n* = 5) mice were analyzed for CXCL9 production by flow cytometry. Dots represent biological replicates pooled from two independent experiments, horizontal bars represent mean, error bars represent SD. **f** AUC scores for immunogenic or tolerogenic maturation signatures^24^ within cDC1 clusters. **g** Expression of indicated genes in cDC1 clusters, irrespective of genotype. Box represents interquartile range, horizontal bar represents median, whiskers represent minimum and maximum enrichment scores (**d**, **f**). *p*-values were determined using two-tailed Wilcoxon ranked sum test with Benjamini–Hochberg adjustment for multiple comparisons (**d**, **f**), two-way ANOVA with two-tailed Šídák’s multiple comparisons test (**e**). For (**f**), only comparisons performed between clusters 15 and 20 are indicated. Source data are provided as a Source Data file.

To gain further mechanistic insights into the dysregulation of cDC1 and CD8^+^ T cell homeostasis in *Clec9a*^*+/cre*^*Stat1*^*fl/fl*^ mice we performed scRNA-seq of CD90.2^+^ cells (including T cells, NKT cells and innate lymphocytes) and CD11c^+^MHCII^+^ cDCs from spleens of 3-week-old *Clec9a*^*+/cre*^*Stat1*^*fl/fl*^ mice and *Stat1*^*fl/fl*^ littermates (Supplementary Fig. 6a). Unsupervised clustering of CD11c^+^MHCII^+^ cells from both genotypes resulted in 23 clusters, which were assigned as cDC1 (clusters 0, 3, 5, 8, 9, 11, 12, 20, 22), cDC2/DC3 (clusters 1, 2, 6, 7, 10, 14, 16, 17, 18, 19) and tDC (cluster 4) based on published gene signatures (Fig. 6a, Supplementary Fig. 6b–d, and Supplementary Data. 1). The resolution for unsupervised clustering was chosen to reveal *Sirpa-*expressing *Ccr7*^+^ cDC2 (cluster 13) and *Irf8*-expressing *Ccr7*^+^ cDC1^25^ (cluster 15, Supplementary Fig. 6b, c). Again, we identified a small contaminating cluster of *Gypa*-expressing erythrocytes (cluster 21)^47^, which was excluded from further analyses (Supplementary Fig. 6b).

Cells from both genotypes contributed equally to individual clusters assigned as cDC2/DC3 and tDC (Supplementary Fig. 6e), while within cDC1, cluster 20 was dominated by cells from *Stat1*^*fl/fl*^ controls (Fig. 6b). As expected, *Stat1* transcripts were reduced across cDC clusters from *Clec9a*^*+/cre*^*Stat1*^*fl/fl*^ mice, confirming efficient *Stat1* deletion (Supplementary Fig. 6g). cDC1 cluster 20 expressed highest levels of *Cxcl9* and *Cxcl10* compared to other cDC1 clusters and lacked *Ccr7* (Fig. 6c and Supplementary Data. 8). Cluster 20 transcriptionally differed from the homeostatically matured *Ccr7*^+^ cDC1 cluster 15, which highly expressed *Il12b* and *Cd200* (Fig. 6c, d, Supplementary Fig. 6c, and Supplementary Data 8). In an intravenous labeling approach CCR7^+^ DCs poorly labeled (Supplementary Fig. 6f), consistent with their localization in the white pulp^25^. In contrast, CXCL9^+^ DCs strongly labeled, indicating localization in blood exposed spleen regions, like red pulp or marginal zone (Supplementary Fig. 6f). *Cxcl9* expression in cluster 20 was lower in cells from *Clec9a*^*+/cre*^*Stat1*^*fl/fl*^ mice compared to control mice (Fig. 6d). Thus, not only fewer of the *Stat1*-deficient cDC1 acquire this transcriptional state but the ones that do express lower levels of *Cxcl9* (Fig. 6d). Of note, specifically cDC1, but not cDC2 or tDCs, showed reduced CXCL9 production in the absence of STAT1 (Fig. 6e). cDC1 cluster 20 showed high *Stat1* expression and exhibited an enrichment of IFNα and IFNγ response genes (Supplementary Fig. 6g, h), indicating it represents a distinct transcriptional state of cDC1 shaped by IFN signaling. To assess if a similar state was contained within a larger cluster in the scRNA-seq dataset across age (Fig. 2), we isolated and reclustered the cDC1 and migratory DC clusters from this dataset at higher resolution. When comparing the original annotation with the new cluster annotations and their dynamics over time, we confirmed that cells within the original cluster 0 declined with age, while cells contained within original cluster 1 were most prominent at week 3 (Supplementary Fig. 7a–e). Notably, we found a distinct state marked by high levels of *Cxcl9* and *Cxcl10* (new cluster 9) that had previously been included in cluster 1 (Fig. 2c and Supplementary Fig. 7a–d). This new cluster 9 increased in frequency between one and three weeks of age (Supplementary Fig. 7e, f).

PRR-induced immunogenic cDC1 maturation is distinguished from homeostatic cDC1 maturation by expression of specific genes^24^. Among cDC1 clusters, *Ccr7*^+^ cDC1 (cluster 15) from *Clec9a*^*+/cre*^*Stat1*^*fl/fl*^ and *Stat1*^*fl/fl*^ mice showed highest expression of genes associated with homeostatic cDC maturation^24^, whereas cDC1 cluster 20 expressed genes associated with immunogenic cDC maturation (Fig. 6f). Further, cluster 20 expressed a variety of co-stimulatory molecules indicative of mature antigen presenting cells, including *Cd80*, *Cd86*, *Cd83*, and *Cd40*, as well as *Icam1*, which stabilizes the immunological synapse^68^ (Fig. 6g). Notably, Cluster 20 also expressed highest levels of the IFN-responsive cytosolic serpin *Serpina3f* (Fig. 6g) that is part of a family of proteins that protects from cell death, including cytotoxic T cell-induced killing^69,70^. Taken together, these data show that STAT1-signaling in cDC1 controls the emergence of a unique transcriptional maturation state of cDC1.

### Loss of *Stat1* in cDCs impairs the communication between cDC1 and cytotoxic CD8^+^ T cells

**Fig. 7:**
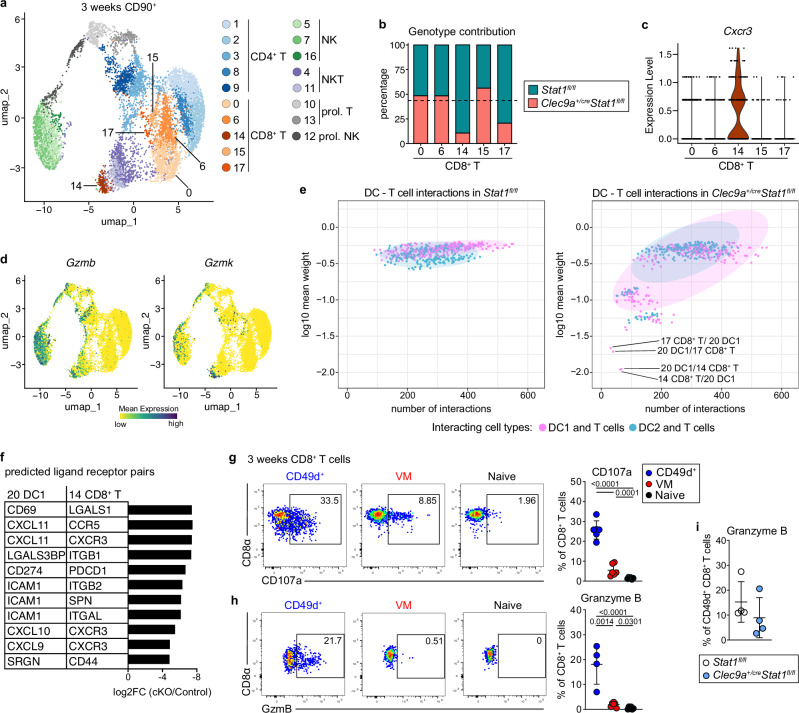
Loss of STAT1-signaling in cDCs impairs the communication between cDC1 and cytotoxic T cells. **a–d** scRNA-seq of CD90^+^ cells from spleens of 3-week-old *Clec9a*^*+/cre*^*Stat1*^*fl/fl*^ and *Stat1*^*fl/fl*^ mice. **a** UMAP display of 8995 CD90^+^ cells from both genotypes annotated by cell type (*Clec9a*^*+/cre*^*Stat1*^*fl/fl*^: 4393, *Stat1*^*fl/f*^: 4602). **b** Contribution of cells from each genotype to CD8^+^ T cell clusters. Dotted line is the contribution of *Clec9a*^*+/cre*^*Stat1*^*fl/fl*^ genotype CD8^+^ T cells to all CD8^+^ T cell clusters combined. **c** *Cxcr3* expression in CD8^+^ T cell clusters. **d** Expression of *Gzmb, Gzmk* projected on the UMAP independent of genotype. **e**, **f** *Community* interactome analysis between cDC and T cell clusters. **e** Number of interactions and average interaction intensity between of DC–T cell clusters from *Stat1*^*fl/fl*^ and *Clec9a*^*+/cre*^*Stat1*^*fl/fl*^ mice is shown. **f** Predicted ligand-receptor pairs between cluster 20 cDC1 and cluster 14 CD8^+^ T and respective log2FC of interaction intensity in *Clec9a*^*+/cre*^*Stat1*^*fl/fl*^ over *Stat1*^*fl/fl*^ control are shown. Pairs were filtered for interactions including ligands or receptors that showed differential gene expression in cluster 20 cDC1 vs. other cDC1 clusters or in cluster 14 CD8^+^ T vs. other CD8^+^ T cell clusters. **g**, **h** The indicated CD8^+^ T cell subsets sorted from spleens of 3-week-old wild type mice were stimulated with anti-CD3/anti-CD28 followed by staining for CD107a (*n* = 6) (**g**) or with PMA/ionomycin and stained for Granzyme B (CD49d^+^ (*n* = 4); virtual memory (VM) and naïve (*n* = 6)) (**h**). **i** CD49d^+^ CD8^+^ T cells were sorted from the spleens of 3-week-old *Clec9a*^*+/cre*^*Stat1*^*fl/fl*^ and *Stat1*^*fl/fl*^ mice and stimulated with PMA/ionomycin as above. The frequency of Granzyme B expression is shown (*n* = 4). Each dot represents one biological replicate pooled from two independent experiments, horizontal bars represent mean, error bars represent SD. *p*-values were determined using one-way ANOVA with two-tailed Tukey’s multiple comparisons and are indicated for statistically significant comparisons. Source data are provided as a Source Data file.

We next performed unsupervised clustering of the CD90.2^+^ cells (Fig. 7a). This resulted in 20 clusters (Fig. 7a), two of which were excluded as monocyte contamination because they lacked *Cd3e* and *Thy1* and expressed typical monocyte markers (*Lyz2*, *Adgre4*) and genes associated with antigen presentation (Supplementary Data 9). As expected, the remaining 18 clusters could be identified as *Klrb1c*^*+*^ NK cells (clusters 5, 7, 16), *Klra1*^+^, *Klra7*^+^, *Cd3e*^+^ NKT cells (Clusters 4, 11), and *Cd3e* expressing CD4^+^ and CD8^+^ T cells (Fig. 7a and Supplementary Fig. 8a). Of these, clusters 10, 13, and 12 contained mostly proliferating cells (Supplementary Fig. 8b). Cells from both genotypes contributed equally to individual clusters except for CD8^+^ T cell clusters 14 and 17, which were predominated by cells from *Stat1*^*fl/fl*^ control mice (Fig. 7b and Supplementary Fig. 8c). Importantly, *Stat1* expression was similar between cells from *Clec9a*^*+/cre*^*Stat1*^*fl/fl*^ and *Stat1*^*fl/fl*^ control mice, confirming that *Clec9a*^*cre*^ does not delete *Stat1* in CD90^+^ lymphocytes and any effects observed in T cells from *Clec9a*^*+/cre*^*Stat1*^*fl/fl*^ mice are secondary to deletion of *Stat1* in cDCs (Supplementary Fig. 8d). Clusters 0 and 6 most closely resembled naïve T cells (Supplementary Fig. 8e). Cluster 15 showed highest expression of *Eomes* suggesting these cells constituted virtual memory T cells^61^ (Supplementary Fig. 8e). Notably, cluster 17 expressed highest levels of *Stat1* and (Supplementary Fig. 8d) and showed evidence of IFN-regulated genes (Supplementary Data 9). Expression of *Sell* (encoding CD62L), *Cd44*, *Itga4* (encoding CD49d), and *S100a4*, identified cluster 14 as CD49d^+^ CD8^+^ T_EM_ cells, which, among CD8^+^ T cell clusters, showed highest expression of *Cxcr3*, the receptor for CXCL9, −10, and −11 (Fig. 7c and Supplementary Fig. 8e). Cell–cell communication analysis between cDC and T cell clusters using *Community*^71^ revealed that the sender-receiver pairs 20 cDC1/14 CD8^+^ T (and reverse) as well as 20 cDC1/17 CD8^+^ T showed strongest reduction in the number and weight of predicated interactions in *Clec9a*^*+/cre*^*Stat1*^*fl/fl*^ mice compared to control mice (Fig. 7e). The expression of *Ccr7* on CD8^+^ T cell cluster 17, but not on cDC1 cluster 20, suggested that these cells reside in distinct regions of the spleen, leading us to focus on the interactions between cluster 20 cDC1 and cluster 14 CD8^+^ T cells. Community predicted CXCR3 and ICAM1-mediated interactions between these clusters (Fig. 7f). Cluster 14 further expressed high levels of *Gzmb*, *Gzmk*, *Ccl5*, and *Cx3cr1*, markers for cytotoxic CD8^+^ T_EM_ cells^72,73^ (Fig. 7d and Supplementary Fig. 8e). Indeed, after stimulation, sorted CD49d^+^CD44^+^ CD8^+^ T cells stained strongest for Granzyme B and the degranulation marker CD107a compared to virtual memory and naïve CD8^+^ T cells, confirming their effector phenotype (Fig. 7g, h). Of note, CD49d^+^CD44^+^ T_EM_ cells from *Clec9a*^*+/cre*^*Stat1*^*fl/fl*^ mice showed comparable or slightly lower expression of Granzyme B compared to T_EM_ cells from *Clec9a*^*+/+*^*Stat1*^*fl/fl*^ control mice (Fig. 7i), indicating that the few CD49d^+^CD44^+^ T_EM_ cells that remain in *Clec9a*^*+/cre*^*Stat1*^*fl/fl*^ mice possess effector function. Thus, loss of *Stat1* in cDCs specifically impairs the communication of a CXCL9-expressing state of cDC1 with CD8^+^ effector memory T cells in the spleen.

### Chow diet boosts CXCL9 production from splenic cDC1 and the effector differentiation of food antigen-specific CD8^+^ T cells

**Fig. 8:**
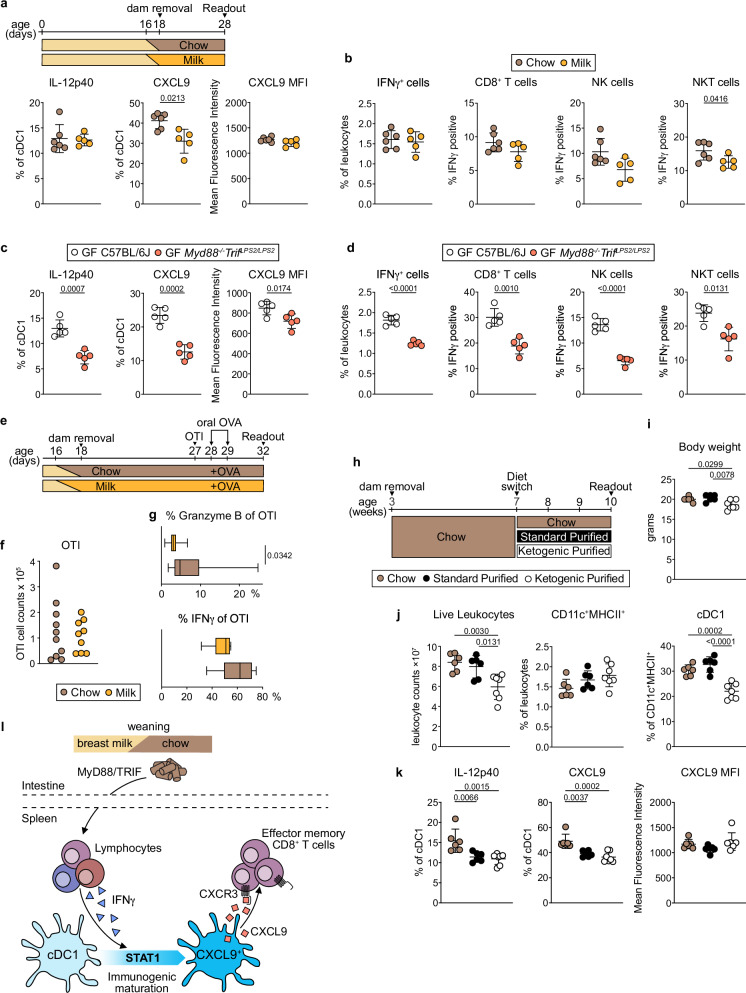
Chow diet boosts CXCL9 production from splenic cDC1 and the effector differentiation of food antigen specific CD8^+^ T cells. **a**, **b** SPF mice were either conventionally weaned and separated from dams on day 18 (chow, *n* = 6) or weaning was delayed by placing mice on formula milk from day 16 while restricting access to chow (milk, *n* = 5). Mice were separated from dams at day 18 and milk diet was continued until day 28 after birth. **a** Frequency of IL-12p40^+^ and CXCL9^+^ cDC1. **b** Frequency of IFNγ-positive leukocytes, CD8^+^ T cells, NK and NKT cells. **c**, **d** Splenocytes from 3-week-old germ-free (GF) *Myd88*^*−/−*^*Trif*^*LPS2/LPS2*^ or age-matched GF wild type controls were analyzed (*n* = 5 from one experiment). **c** Frequency of IL-12p40^+^ and CXCL9^+^ cDC1. **d** Frequency of IFNγ^+^ leukocytes, CD8^+^ T cells, NK and NKT cells is shown. **e–g** Weaning was delayed in SPF mice as in (**a**). At 27 days old mice received 1 × 10^5^ naïve OTI cells. On the next two consecutive days, mice were gavaged with OVA and three days after the last gavage, OTI T cells in spleens were analyzed. **e** Experimental scheme. **f** Number of OTI cells (chow (*n* = 10); milk (*n* = 9). **g** Frequency of IFNγ- and Granzyme B- producing OTI T cells in spleen. (GzmB: chow, *n* = 10; milk, *n* = 9), IFNγ (chow, *n* = 7; milk, *n* = 6). **h–k** Female mice were weaned and maintained on chow (*n* = 6) or switched to a standard purified (*n* = 6) or ketogenic purified (*n* = 7) diet at 6–7 weeks-old for three weeks. **i** Body weight at readout. **j** Spleen leukocyte counts, frequency of CD11c^+^MHCII^+^ cells and frequency of cDC1 within CD11c^+^MHCII^+^ cells. **k** Quantification of IL-12p40^+^ and CXCL9^+^ splenic cDC1. Dots represent biological replicates representative of two independent experiments (**a**, **b**) or pooled from two (**g**, **h–k**, **f**, IFNγ) or three (**f**, GzmB) independent experiments. Horizontal bars represent mean, error bars represent SD. Statistical analysis: two-tailed Welch’s *t*-test (**a–d**), linear mixed-effects models (two-tailed) (**g**), or one-way ANOVA with two-tailed Tukey’s multiple comparisons (**i–k**). *p*-values for statistically significant comparisons are shown. **l** Model of the diet-driven regulatory circuit. Source data are provided as a Source Data file.

In infants, increased dietary complexity correlates with systemic immune alterations, including higher plasma IFNγ levels^74,75^. To directly assess if weaning associated dietary changes influence CXCL9 production from splenic cDC1, we first induced a delay in weaning by restricting access to chow and prolonging milk diet after pups were separated from the mothers^4^ (Fig. 8a). Indeed, one week after separation from the mothers, cDC1 from mice weaned onto milk showed lower CXCL9 production than cDC1 from mice weaned normally onto chow (Fig. 8a). Similarly, IFNγ production in CD8^+^ T, NK and NKT cells was lower in mice weaned onto milk than in mice weaned onto chow (Fig. 8b). Chow contains heterogeneous plant-derived fibers and other ill-defined contents, such as lipopolysaccharide (LPS)^76,77^. These are not found in breast milk or purified mouse diets and can trigger TLR-mediated immune responses independent of the microbiota^76,77^. Indeed, CXCL9 production from spleen cDC1 and IFNγ production from CD8^+^ T, NK, and NKT cells was lower in 3-week-old GF *Myd88*^*−/−*^*Trif*^*LPS2/LPS2*^ compared to wild type mice (Fig. 8c, d and Supplementary Fig. 9a). These data suggest that during weaning dietary components in chow trigger a *Myd88/Trif*-dependent immune response independent of the microbiota that, via IFNγ production from lymphocytes, is relayed to splenic cDC1 and boosts their CXCL9 production.

Antigen-experienced CD8^+^ T_EM_ cells in steady state SPF and GF mice are thought to arise in response to environmental antigens, such as food. Indeed, we observed an increase in the percentage and number of CD49d^+^CD44^+^ CD8^+^ T cells in spleens between two and 3 weeks of age, which correlates to the onset of weaning (Supplementary Fig. 9b). To test if weaning influences the effector phenotype of food-antigen specific CD8^+^ T cell in spleens, we either weaned mice conventionally or onto a milk diet as above. Nine days after separating pups from the mothers, we transferred naïve OTI T cells into these mice, and subsequently provided OVA as food antigen or PBS as control (Fig. 8e and Supplementary Fig. 9c). Three days after the last OVA feeding, we analyzed OTI T cells in spleens. We first confirmed antigen specificity of the response in both diets, observing robust recovery of OTI T cells only in spleens of mice that had received OVA, but not PBS as control (Supplementary Fig. 9d, e). Similar numbers of OTI T cells were recovered when comparing milk and chow groups (Fig. 8f and Supplementary Fig. 9e). However, we observed higher Granzyme B and IFNγ production in OTI T cells recovered from spleens of mice on chow compared to milk diet, indicating more robust effector differentiation (Fig. 8g).

Finally, we asked if dietary intervention in adulthood could alter the maturation of cDC1 into CXCL9-producing cells. We weaned mice conventionally onto standard chow and at 6–7 weeks of age, we either kept mice on chow or fed them with a ketogenic purified or standard purified diet for three weeks ad libitum (Fig. 8h). Both purified diets are similarly formulated and lack complex plant-derived fibers and other potentially bioactive compounds. A standard purified diet mirrors chow in macronutrient ratio, while a ketogenic purified diet shares significant metabolic similarities with breastfeeding—both are high in fat, low in carbohydrates and protein^78,79^. As expected^80^, mice fed ketogenic diet exhibited lower body weight compared to mice fed standard chow or purified control diet (Fig. 8i). We also observed reduced spleen cellularity in mice fed ketogenic diet (Fig. 8j). The frequency of CD11c^+^MHCII^+^ cells within splenocytes was similar across diets but we observed a relative reduction of XCR1^+^ cDC1 specifically in mice fed ketogenic diet compared to standard purified or chow diet (Fig. 8j), suggesting that cDC1 abundance is sensitive to fat metabolism. However, CXCL9 and IL-12p40 production from cDC1 were reduced in mice fed with either of the purified diets compared to standard chow diet (Fig. 8k). Taken together, we show that dietary components in chow trigger a *Myd88/Trif*-dependent immune response independent of the microbiota. This response is relayed to the spleen via IFNγ production from lymphocytes, which equips cDC1 immunostimulatory functions that shape the effector differentiation of antigen-experienced cytotoxic CD8⁺ effector T cells during weaning (Fig. 8l).

## Discussion

While the regulatory circuits that promote T cell tolerance in early life are well studied, the signals that determine immune stimulation remain elusive. Here, we identify a diet-driven, IFNγ-mediated regulatory circuit that relays dietary information to cDC1 in the spleen. IFNγ-stimulated cDC1 tune cytotoxic effector-like CD8^+^ T cells independent of the microbiota. During weaning, this circuit regulates food-antigen specific CD8^+^ T cells in a feed-forward fashion. cDC1 maturation remained responsive to dietary intervention in adult mice, highlighting the potential to harness diet to modulate cDC function therapeutically or in vaccination beyond the weaning period.

We identified steady-state IFNγ−mediated STAT1 activation as a driving factor for a specific CXCL9^+^ maturation state of cDC1 transcriptionally distinct from CCR7^+^ cDC1. Although IFNγ signaling regulates transcriptional targets beyond *Cxcl9*, this chemokine serves as a representative marker allowing detection by flow cytometry. This cDC1 state expresses genes associated with immunogenic DC maturation after PAMP stimulation^24^, but it arises in the absence of pathogens and even under GF conditions, showing it is the result of homeostatic maturation. cDC-intrinsic loss of *Stat1* reduces cytotoxic CD8^+^ T_EM_ cells in spleen, suggesting that the homeostatic maturation of cDC1 is not exclusively tolerogenic. Prior work had identified a similar cDC1 state by scRNA-seq in adult mouse spleen and trajectory analysis suggested these cells represent a transient “early mature” stage of tolerogenic cDC1 maturation into CCR7^+^ cells^25^. Whether STAT1-regulated *Cxcl9*^*+*^ cDC1 are part of a single linear maturation trajectory or represent a distinct maturation state of cDC1 remains to be determined. CD8^+^ T cell responses in infections and tumors are subject to exact spatiotemporal regulation involving IFNγ and CXCL9 cross-talk^81–86^. Tumor-resident CXCL9-expressing cDC1 that lack CCR7 induce the local activation of protective anti-cancer CD8^+^ T cell responses^17,87^. Similarly, our i.v. labeling and transcriptional data indicate that the interaction of CD8^+^ T cells and homeostatically matured CXCL9^+^ cDC1 takes place outside the T cell zone, likely in blood exposed regions, such as the marginal zone or red pulp, known sites for the entry, regulation and bystander activation of circulating memory CD8^+^ T cells^81–84^.

*Stat1* deletion did not abrogate CXCL9 production in all cDC1, indicating that additional signals regulate CXCL9 expression in cDC1. Indeed, loss of STING causes a reduction of CXCL9 production from cDC1 in steady state SPF mice^25^. STING-mediated CXCL9 production could be linked to recognition of self-DNA from apoptotic cells or recognition of bacterial DNA that can be delivered into host cells from microbiota-derived membrane vesicles^25,88,89^. Both signals would trigger the cytosolic cGAS/STING pathway, which can induce production of IFN-I and subsequent CXCL9 production from cDC1^25,89^. Signals driving CXCL9 in cDC1 could differ by age and CXCL9 production in cDC1 may even be regulated in a tissue-specific manner.

Dietary supplementation and nutritional interventions are increasingly used to modulate immunity to reduce inflammation or alter immune responses, yet nutrition remains a poorly understood regulator of immunity, in part because altering diet also changes host-microbial cross-talk^90–93^. Chow contains various micronutrients and other poorly defined bioactive components, including traces of microbial components, such as LPS, or beta-glucan, a soluble fiber found in the cell walls of grains^94^, that can trigger PRR signaling and cytokine production from immune cells^77,95–97^. Dietary LPS, for instance activates mucosal immunity and shapes the intestinal IgA repertoire in germ-free mice^76^. Our data suggest that Myd88-TRIF activating bioactive dietary components initiate the here identified IFNγ-mediated regulatory circuit, although we cannot exclude that mechanical forces related to dietary consistency trigger Myd88-TRIF dependent immune activation. Further work is needed to define the exact dietary components sensed, if they are sensed locally in the intestine or elsewhere, and the exact cell types involved. Vitamin C has been shown to directly modify lysine residues in STAT1, which prolongs STAT1 phosphorylation and enhances anti-tumor immunity in mice^98^. STAT1 induces IFNγ production^52^, raising the possibility that dietary supplementation of Vitamin C could be used to promote the maturation of cDC1 into an immunostimulatory state by inducing IFNγ production from lymphocytes or by promoting STAT1 phosphorylation in cDC1 themselves. Mice can synthesize Vitamin C from glucose, making this pathway irrelevant in the murine model; however, humans require Vitamin C from food and dietary supplementation with Vitamin C is commonly used to boost anti-viral immunity^99^. If a specific IFNγ-producing lymphocyte population drives CXCL9 production from cDC1 remains to be determined, as well as whether these IFNγ-producing lymphocytes, specifically CD8^+^ T cells, originate from the intestine. Since diet can modulate the CXCL9^+^ cDC1 state in adult mice, it will be interesting to determine if diet-induced changes in IFNγ production regulate cDC1 even after periods of malnutrition or if chow diet is introduced at later stages in life.

Steady state CD49d^+^CD8^+^ T_EM_ cells likely recognize environmental or food-derived antigens^100,101^. At three weeks of age, these cells showed transcriptional and functional similarity with cytotoxic CD8^+^ T_EM_ cells, including ability to rapidly degranulate and high expression of Granzymes and *Cx3cr1*^72^. The few CD8^+^ T_EM_ cells that are found in the spleens of *Clec9a*^*+/cre*^*Stat1*^*fl/fl*^ mice still produce Granzyme B upon stimulation, suggesting intact priming. CD49d^+^CD8^+^ T_EM_ cells are likely primed in the periphery against environmental antigens, including food. Antigens encountered via the diet normally induce T cell tolerance, although such tolerance mechanisms are responsive to the context in which dietary antigens are sampled^102–104^. Initial priming and effector differentiation of food antigen-specific T cells in gut-associated lymphoid tissues will depend on the nature of the antigen (soluble vs. cell-associated), dietary context and the type of antigen-presenting cell involved (cDC1 or other antigen-presenting cells)^103–106^. Following priming, antigen-experienced CD8^+^ T cells enter the circulation akin to T cells primed against pathogens and continuously recirculate through the spleen—the primary site of blood filtration—where they enter via blood-exposed regions such as the red pulp and marginal zone^107^.

Dietary context changes during the transition from milk to solid food and our data show that the immune system systemically relays dietary cues during this transition via IFNγ to spleen cDC1. IFNγ-stimulated CXCL9⁺ cDC1 lacked CCR7 and had access to blood, suggesting their localization in blood-exposed regions of the spleen. This positioning would place CXCL9⁺ cDC1 in a strategic location to interact with recirculating antigen-experienced CD8^+^ T cells after their entry into the spleen. We propose that IFNγ-stimulated CXCL9⁺ cDC1 shape these antigen-experienced CD8^+^ T cells by providing additional tissue-specific cues that reinforce or modify the functional state of these CD8^+^ T cells after their initial priming in the intestine or elsewhere. Thus, rather than determining whether dietary or other environmental antigens elicit tolerance or immunity, this regulatory circuit may allow the immune system to systemically tune the effector state of antigen-experienced CD8^+^ T cells according to dietary context in a feed-forward manner.

Food-antigen specific CD8^+^ T cells were more cytotoxic in the spleens of weanling mice on chow compared to milk diet, demonstrating that dietary context influences the acquisition of effector function by these cells. Whether this heightened effector state contributes to host protection when dietary antigens are encountered in an inflammatory setting, facilitates tissue surveillance, or supports mechanisms underlying oral tolerance, for instance by eliminating cells presenting food allergens, remains to be determined. To experimentally address these possibilities, model antigens that elicit CD8^+^ T cell responses, such as SIINFEKL, could be fed under different dietary conditions prior to allergic challenge or infection with epitope-containing pathogens. It is interesting to note that effective and sustained desensitization against food allergens following oral immunotherapy has been linked to an increased frequency of cytotoxic CD8^+^ T_EM_ cells^108^. Our data provide a mechanistic framework for this observation and suggest that diet may influence oral immunotherapy not only by influencing T cell priming in the gastrointestinal tract but also by shaping the functions of antigen-presenting cells at distant sites in a feed-forward manner.

Together, our study identifies a previously unrecognized, microbiota-independent regulatory circuit by which dietary cues are relayed through IFNγ to imprint spleen-resident cDC1 with immunostimulatory functions. This IFNγ signal enables cDC1 to shape the effector differentiation of antigen-experienced CD8⁺ T cells in early life to tailor the T cell pool to developmental stage. These findings redefine the landscape of steady-state cDC1 maturation, challenging the prevailing view that homeostatic cDC1 maturation primarily promotes T cell tolerance. By uncovering a critical link between nutrition and cDC function that remains responsive to dietary interventions in adulthood, our work opens new avenues for leveraging dietary interventions to modulate cDC-driven immunity in vaccination or disease.

## Methods

### Mice

Clec9a^tm2.1(icre)Crs^ (*Clec9a*^*Cre*^) (Jackson Laboratory Stock No: 025523), Gt(ROSA)26Sor^tm9(CAG-tdTomato)Hze^ (*Rosa26*^*lox-STOP-lox-tdtomato*^) (Jackson Laboratory Stock No: 007909), Ifnar1^tm1Agt^ (*Ifnar*^*−/−*^) (MMRRC Stock No: 32045-JAX), B6(Cg)-Ifnar1tm^1floxUka^ (*Ifnar1*^*fl/fl*^, generated by Ulrich Kalinke), Tg(Itgax-cre)1-1Reiz (*Itgax*^*cre*^) (Jackson Laboratory Stock No: 018967), Stat1^tm1.1Mmul^ (*Stat1*^*fl/fl*^)^109^, C57BL/6-Tg(Nr4a1-EGFP/cre)820Khog-Ptprca (*Nr4a1*^*eGFP*^) (Jackson Laboratory Stock No: 016617), OTI mice (C57BL/6-Tg(TcraTcrb)1100Mjb/J, Jackson Laboratory stock no: 003831) and C57BL/6JRccHsd mice (RRID:IMSR_ENV:HSD-043) were bred and maintained at the Biomedical Center, LMU Munich. B6.129S7-Ifngr1^tm1Agt^/J mice were maintained at the VIB Center for Inflammation Research Ghent (Jackson Laboratory Stock No: 003288). C57BL/6J (Jackson Laboratory stock no: 000664) wildlings were created through inverse germ-free rederivation^58^. SPF mice were maintained in individually vented cages with a 12 h dark/light cycle. Cage manipulations took place in laminar flow hoods. Air temperature was 22 ± 2 °C and humidity 55 ± 10% with daily control and record. Wildling mice were bred at the Medical Center, University of Freiburg, Germany, germ-free animals were bred at the ZIEL institute for Food & Health TUM, Freising, Germany in isolators and germ-free status was routinely monitored. Germ-free *Myd88*^*−/−*^*Trif*^*LPS2/LPS2*^ mice^110^ were bred and maintained in flexible-film isolators at the Clean Mouse Facility, University of Bern, Switzerland. Food and water were provided ad libitum. Unless otherwise specified mice were housed on chow as a grain-based diet. Chow diets may exhibit considerable batch-to-batch variation, but are used in most experimental facilities worldwide, so that we did not normalize chow diets across facilities. Mice were fed on of the following standard chow diets ad libitum: Ssniff (V1534) for germ-free or ketogenic diet experiments; Kliba Nafag (3307) for *Myd88*^*−/−*^*Trif*^  *LPS2/LPS2*^ experiments; Kliba Nafag (3807) for wildling experiments, or Altromin (1314) for all other experiments. Male and female mice were used in this study. Female mice were used for ketogenic diet experiments to allow for stable group-housing. Littermates were used in experiments unless otherwise stated and data from male and female mice has been pooled as no sex-specific differences have been observed. All animal procedures were performed in accordance with national and institutional guidelines for animal welfare and approved by Regierung von Oberbayern, Regierungspräsidium Freiburg, the Cantonal Commissions for Animal Experimentation of the Cantone of Berne and VIB site Ghent–Ghent University Faculty of Science.

### Delayed weaning

We delayed weaning following an established protocol^4^. Litter sizes between the formula-fed (milk) and chow-fed groups were equalized one week after birth. Between postnatal days 16 and 18, solid food (chow) was removed from the cage of the milk-fed group and the pups received 15 µL of Optima Pet Milk (TVM) by oral gavage 8 times per day. The dam was also fed with formula milk provided in sterile bottles during this time. The chow group had continuous access to standard chow diet and served as the control. Eighteen days after birth, the dams were removed from both cages and the pups in the formula-fed group continued to receive formula milk only via bottle. Bottles were replaced three times a day to prevent bacterial contamination, cages were changed twice a day to control for coprophagy. Animals from both groups were analyzed at 4 weeks of age.

### Food antigen response of OTI T cells

Adoptive transfer of OTI T cells and oral OVA administration was performed within the delayed-weaning experimental setting described above. Nine days after being separated from the mothers, CD45.2 congenic pups were injected intravenously with 1 × 10⁵ naïve OTI T cells isolated from adult CD45.1/2 congenic OTI mice using the MojoSort™ Mouse CD8 Naïve T Cell Isolation Kit (BioLegend, 480044). 1 and 2 days after adoptive OTI T cell transfer mice were gavaged with 50 mg OVA (Grade III, Sigma-Aldrich, A5378) in 100 µL PBS or with 100 µL PBS as control. 3 days after final OVA administration mice were analyzed. A CD45.2⁺ mouse that did not receive OTI T cells served as a control to set the gate for injected OTI T cells.

### Ketogenic diet

Six to seven-week-old female mice were fed standard chow diet (Ssniff, V1534), standard purified diet (AIN-93M, powder, Bio-Serv, F3198) or ketogenic purified diet (AIN-76A Modified, High Fat, Paste, Bio-Serv, F3666) for 3 weeks ad libitum (Supplementary Data 12). All diets used in this study were sterilized either by autoclaving or irradiation.

### Cell isolation

Mice were euthanized by cervical dislocation. Mice younger than 3 weeks were euthanized by decapitation. Spleens were isolated, minced and enzymatically digested in 1 mL RPMI 1640 Medium (Thermo Fisher Scientific, 31870-074) containing 200 U/mL Collagenase IV (Worthington, LS004189) and 0.2 mg/mL DNaseI (Roche, 11284932001) for 30 min at 37 °C while shaking (180 rpm), then passed through a 70 μm strainer for all experiments, except when wildling mice were used. For wildling experiments, splenocytes were isolated by mechanical disruption; spleens were mashed through a 70 μm cell using a syringe plunger. The tubes were then filled with ice-cold FACS buffer [PBS 1% fetal calf serum (FCS, Sigma-Aldrich, F7524), 2.5 mM EDTA (Thermo Fisher Scientific, 15575020) with 0.02% sodium azide (Sigma-Aldrich, 71289)] and centrifuged. Subsequently, erythrocytes were osmotically lysed in 1× Ammonium-chloride-potassium buffer [prepared from a 10× stock solution: MilliQ water with 1 mM EDTA (Thermo Fisher Scientific, 15575020), 1.55 M NH_4_Cl (Sigma-Aldrich, A9434) and 100 mM KHCO_3_ (Sigma-Aldrich, 237205)] for two minutes at 4 °C, and washed with FACS buffer. Cell pellets were resuspended in FACS buffer and passed again through a 70 μm strainer (pluriSelect, 43-50070-50/51) before further analysis. For functional analyses and scRNA-seq experiments PBS containing 1% FCS and 2.5 mM EDTA was used for cell isolation.

### Flow cytometry

4 × 10^6^ cells were stained in 50 µL FACS buffer with anti-mouse CD16/32 (Fc-block) for 10 min at 4 °C. 50 µL of a 2× mastermix containing antibodies against surface epitopes and fixable viability dye eFluor™ 780 (Thermo Fisher Scientific, 65-0865-14) was added and cells were incubated at 4 °C for 25 min. Cells were then washed twice and resuspended in FACS buffer for analysis. After surface staining, intracellular cytokine staining was performed using the Intracellular Fixation & Permeabilization Buffer Set (Thermo Fisher Scientific, 88-8824-00); intranuclear markers and Granzyme B were stained using the Foxp3 Transcription Factor Staining Set (Thermo Fisher Scientific, 00-5523-00) according to the manufacturer’s instructions. CountBright™ Absolute Counting Beads (Thermo Fisher Scientific, C36950) were added to obtain cell counts as previously described in ref. ^111^. For intracellular cytokine staining of lymphocytes, splenocytes were stimulated with 10 ng/mL phorbol 12-myristate 13-acetate (PMA, Calbiochem, 524400) and ionomycin (1 µg/mL, Sigma-Aldrich, I9657) for 5 h at 37 °C. Brefeldin A (5 µg/mL, Biolegend, 420601) was added for the last 3 h. Prior to IL-12p40 and CXCL9 staining, splenocytes were incubated for 4 h with Brefeldin A (5 µg/mL, Biolegend, 420601) and Monensin (2 µM, Sigma-Aldrich, M5273) at 37 °C. MAIT cells were stained with BV421-conjugated mouse MR1-5-OP-RU tetramers at room temperature for 30 min. 6-FP-loaded MR1 tetramers were used as a negative control^112^. Antibodies used for flow cytometry are provided in the Supplementary Data 10. Data was collected on a LSR Fortessa (BD Biosciences) using BD FACSDiva Software (BD BioSciences, version 8) and analyzed using FlowJo software (Tree Star, Inc.). Mean fluorescence intensity (MFI) of CXCL9 was calculated as the geometric mean of the indicated fluorescent parameter within CXCL9^+^ cells. Cell sorting was performed on a FACSAria Fusion (BD Biosciences).

### In vitro stimulation with PAMPs

For in vitro stimulation of cDC1 with PAMPs, splenocytes from spleens of 2-week-old or adult mice were enriched using CD11c MicroBeads (Miltenyi, 130-125-835) according to the manufacturer’s recommendation. Two to four spleens from young mice of the same sex were pooled to obtain sufficient cell numbers. Cells were then stained as above in PBS containing 1% FCS and 2.5 mM EDTA and cDC1 were sorted into the PBS containing 10% FCS according to the gating strategy in Supplementary Fig. 1a, b). 35,000 XCR1^+^ cDC1 were stimulated with 0.5 µg/mL CpG-B ODN 1826 (InvivoGen, tlrl-1826-1), 10 µg/mL Zymosan A (Sigma-Aldrich, Z4250), 100 µg/mL Zymosan Depleted (InvivoGen, tlrl-zyd), or 10 µg/mL poly(I:C) LMW (InvivoGen, tlrl-picw) in a total volume of 50 µL RPMI containing 10% FCS, 1% Penicillin/Streptomycin (Thermo Fisher Scientific, 15140-122), 1% non-essential amino acids (Sigma-Aldrich, M7145), 1% sodium pyruvate (Sigma-Aldrich, S8636), 1% L-Glutamine (Sigma-Aldrich, G7513), 0.05 mM β-mercaptoethanol (Thermo Fisher Scientific, 31350-010). After 20 h, cells were pelleted and cytokines in supernatants were quantified using LEGENDplex Mouse Inflammation Panel (Biolegend, 740446) and LEGENDplex Mouse Cytokine Panel 2 for IL-12p40 (Biolegend, 740138).

### T cell degranulation assay

Splenocytes from 3-week-old mice were stained with FITC-conjugated antibodies against CD19, Ly6G, Ter119 and CD4. Splenocytes were then depleted of FITC-labeled cells using anti-FITC magnetic beads (Miltenyi, 130-048-701) and LS columns (Miltenyi, 130-042-401) according to manufacturer’s instructions. Subsequently, cells were washed and surface-stained for surface epitopes as described above and then sorted into PBS containing 10% FCS. 9000 naïve CD8^+^ T cells (CD62L^+^CD44^neg^), CD44^+^CD49d^+^ and CD44^+^CD49d^neg^ CD8^+^ T cells were sort-purified and incubated in 96-well v-bottom plates coated with 1 µg/ml LEAF-purified anti-CD3e at 4 °C overnight, in the presence of soluble anti-CD28 (1 µg/ml) for a total of 5 h at 37 °C. Anti-CD107a was added to the culture along with Brefeldin A and Monensin for the last three hours of culture. The cells were then re-stained with viability dye. For granzyme B staining, 9000 sorted cells were incubated with PMA and ionomycin for a total of 5 h. Brefeldin A was added for the last three hours.

### Quantitative real-time PCR

RNA was isolated from spleens using RNeasy Midi Kit (Qiagen, 75144) and subsequently treated with DNase I to remove any residual genomic DNA, followed by enzymatic inactivation using the TURBO DNA-free Kit (Thermo Fisher Scientific, AM1907). Complementary DNA (cDNA) was synthesized using Superscript III reverse transcriptase (Thermo Fisher Scientific, 18080-044) according to manufacturer’s instructions. 2 µg RNA was used for reverse transcriptase reaction. In parallel, a “no RT control” reaction was performed with the same 2 µg RNA input, but no-reverse transcriptase was added. Quantitative real-time PCR performed using SYBR Green Fast master mix (Thermo Fisher Scientific, 4385612) according to the manufacturer’s instructions, on a Real-Time PCR system (Applied Biosystems) using primers listed in Supplementary Data 11. For primers that are not exon spanning, qRT-PCR was performed on cDNA samples and respective no-RT controls. Only samples showing specific amplification in the cDNA reaction with no detectable product in the no-RT control were quantified. Quantification was performed by relative standard curve method and target gene expression was normalized to *Actb*. For visualization purposes a normalization factor (×1000) was applied to the relative expression values.

### Single-cell RNA sequencing

Splenocytes from 1-week (*n* = 5), 3-week (*n* = 4), 4-week (*n* = 3) and 6-week (*n* = 3) old *Clec9a*^*+/cre*^*Rosa*^*TOM*^ mice were isolated and stained with FITC-conjugated antibodies against CD19, CD3e and Ter119. Cells were then depleted of FITC-labeled cells using anti-FITC magnetic beads (Miltenyi, 130-048-701) and LS columns (Miltenyi, 130-042-401) according to manufacturer’s instructions. Depleted samples were labeled with barcoded antibodies (anti-MHCI, anti-CD45, TotalSeq-B0305/B0310 anti-mouse Hashtag Antibody, Biolegend) according to time point, and CD11c^+^MHCII^+^ cells were sorted into PBS containing 10% FCS (Supplementary Fig. 2b). Equal numbers of sorted CD11c^+^MHCII^+^ cells from two time points, labeled with different barcoded antibodies, were pooled, pelleted, and then resuspended to 700 cells/µL in PBS containing 0.04% bovine serum albumin (BSA, Sigma-Aldrich, A2153). Each pool was then loaded onto a separate reaction of a Chromium chip (10× Genomics, 1000127). Gene expression and cell surface protein libraries were prepared using the Chromium Next GEM Single Cell 3’ Reagent kit (10× Genomics, 1000268, 1000262, 1000215, 1000242). Concentration and purity of the libraries was assessed with a TapeStation (Agilent). Libraries were multiplexed and sequenced using the recommended sequencing depth on a NextSeq1000 (Illumina).

For scRNA-seq of splenic CD11c^+^MHCII^+^ DCs and CD90^+^ cells from 3-week-old *Clec9a*^*+/cre*^*Stat1*^*fl/fl*^ (*n* = 2) and *Stat1*^*fl/fl*^ littermates (*n* = 2), splenocytes were isolated and depleted of CD19, Ly6G and Ter119 positive cells as above using magnetic beads. Depleted samples were then labeled with barcoded antibodies according to genotype, as described above. After sorting, equal numbers of purified DCs from both genotypes were pooled and resuspended to 1000 cells/µL in PBS containing 0.04% BSA. In the same manner, equal numbers of CD90^+^ cells from both genotypes were pooled and resuspended. Each of the two pools was then loaded onto a separate reaction well of the Chromium Chip (10× Genomics, 1000127). Library generation, quality control and sequencing were performed as described above.

### scRNA-seq analysis

Sequencing data were processed using 10× Genomics Cell Ranger v6.0.0 pipeline and mapped to the mouse genome (mm10) customized to include the sequence of iCRE (GenBank ID: AY056050.1), Tomato (GenBank ID: AY678269.1) and the predicted transcript of the unrecombined Rosa locus. For the dataset across age, raw count matrices and cell surface protein information were aggregated with the Cell Ranger aggregate pipeline. The resulting gene-barcode matrix was loaded into R (v4.3.2) using the *Seurat* (v4.3.0) package. Genes detected in <3 cells and cells expressing <1500 genes or >5% mitochondrial genes were excluded from further analysis. Cells were scored based on the expression of cell cycle associated genes and cell cycle regression was performed according to Seurat’s “Cell-Cycle Scoring and Regression” vignette. Cells of different time points were identified by cell surface protein information of barcoded antibodies. Doublets were identified by dual labeling with both barcoded antibodies and also excluded, along with cells not exhibiting sufficient barcode labeling levels to allow for a clear distinction. The *sctransform* package was used to normalize, scale and find variable features of the dataset. Dimensional reduction by UMAP was based on the first 60 principal components. Louvain clustering was used to cluster the data in an unsupervised manner. The resolution was chosen such that RORγt^+^ DC clustered separately. Differentially expressed genes between clusters were identified using the FindAllMarkers and FindMarkers commands of the *Seurat* package. *AUCell* (v.3.18) package was used to score cells for enrichment of published gene signatures (Supplementary Data 1). Gene set enrichment analysis of hallmark gene sets was performed using the *singleseqgset* package.

For the dataset containing CD11c^+^MHCII^+^ cDCs and CD90^+^ cells from *Clec9a*^*+/cre*^*Stat1*^*fl/fl*^ mice and *Stat1*^*fl/fl*^ littermates, DC and lymphocyte libraries were analyzed separately with *Seurat* in *R*. The *SoupX* package was used to decontaminate cells from ambient RNA and the *scDblFinder* package was used to identify and exclude putative doublets. HTO-based doublet exclusion was performed as described above. For the cDC library, genes detected in <3 cells and cells expressing <1000 genes or >5% mitochondrial genes were excluded from further analysis. For the CD90^+^ lymphocyte library, genes detected in <3 cells and cells expressing <1000 genes or >10% mitochondrial genes were excluded from further analysis. Louvain clustering was used to cluster both DC and CD90^+^ lymphocyte datasets in an unsupervised manner. Identification of differentially expressed genes, scoring of published gene signatures and GSEA was performed as described above. Interactome analysis was performed using *Community*^71^ using aggregated gene expression data of DC and T cell clusters.

### Multiome computational analysis

Cells annotated as cDC1 in the multiomic dataset of splenic CD11c^+^MHCII^+^ cells and MHCII^+^ ILC3s^40^ were computationally isolated, followed by dimensional reduction and unsupervised clustering according to the standard scanpy work flow (https://scanpy-tutorials.readthedocs.io/en/latest/pbmc3k.html). Gene set enrichment analysis between cDC1 from 2-week-old and adult mice was performed using *gseapy* package with default parameters^113^.

### Intravenous labeling

3 μg Pacific Blue-conjugated anti-CD45.2 was injected intravenously into 3-week-old mice. Two minutes post-injection, the mice were euthanized by cervical dislocation, and flow cytometry of splenocytes was performed^25,114^.

### Statistical analysis

Statistical analyses were performed in Prism 10 software (GraphPad) using two-tailed *t*-test with Welch’s correction (unless otherwise stated). Percentage data were logit-transformed prior to statistical testing. For multiple comparisons one-way analysis of variance (ANOVA) with Tukey’s test (unless otherwise stated) was performed. For comparing expression levels and AUC scores in scRNA-seq datasets statistical analysis was performed using two-tailed Wilcoxon ranked sum test, with Benjamini–Hochberg adjustment for multiple comparisons. For analyses of food antigen-specific T cells, linear mixed-effects models (R/lme4 version 2.0-1) were used to account for batch effects, with experiment included as a random intercept and condition as a fixed effect. This approach estimates the contribution of inter-experimental variability and tests condition effects within experiments rather than across pooled data.

### Reporting summary

Further information on research design is available in the Nature Portfolio Reporting Summary linked to this article.

## Supplementary information

Supplementary Information

Description of Additional Supplementary Files

Supplementary Data 1

Supplementary Data 2

Supplementary Data 3

Supplementary Data 4

Supplementary Data 5

Supplementary Data 6

Supplementary Data 7

Supplementary Data 8

Supplementary Data 9

Supplementary Data 10

Supplementary Data 11

Supplementary Data 12

Reporting Summary

Transparent Peer Review file

## Source data

Source Data

## Data Availability

Single-cell RNA sequencing data have been deposited in the GEO repository under the accession number GSE337917. All data are included in the Supplementary Information or available from the authors, as are unique reagents used in this Article. The raw numbers for charts and graphs are available in the Source Data file whenever possible. Source data are provided with this paper.

## References

[1] Hong, J. Y. & Medzhitov, R. On developmental programming of the immune system. *Trends Immunol.* **44**, 877–889 (2023). 37852863 10.1016/j.it.2023.09.004

[2] Kollmann, T. R., Kampmann, B., Mazmanian, S. K., Marchant, A. & Levy, O. Protecting the newborn and young infant from infectious diseases: lessons from immune ontogeny. *Immunity* **46**, 350–363 (2017). 28329702 10.1016/j.immuni.2017.03.009

[3] Torow, N., Hand, T. W. & Hornef, M. W. Programmed and environmental determinants driving neonatal mucosal immune development. *Immunity* **56**, 485–499 (2023). 36921575 10.1016/j.immuni.2023.02.013 PMC10079302

[4] Al Nabhani, Z. et al. A weaning reaction to microbiota is required for resistance to immunopathologies in the adult. *Immunity* **50**, 1276–1288.e1275 (2019). 30902637 10.1016/j.immuni.2019.02.014

[5] Scharschmidt, T. C. et al. A wave of regulatory T cells into neonatal skin mediates tolerance to commensal microbes. *Immunity* **43**, 1011–1021 (2015). 26588783 10.1016/j.immuni.2015.10.016 PMC4654993

[6] Weckel, A. et al. Long-term tolerance to skin commensals is established neonatally through a specialized dendritic cell subgroup. *Immunity* **56**, 1239–1254.e1237 (2023). 37028427 10.1016/j.immuni.2023.03.008 PMC10330031

[7] Sikder, M. A. A. et al. Maternal diet modulates the infant microbiome and intestinal Flt3L necessary for dendritic cell development and immunity to respiratory infection. *Immunity* **56**, 1098–1114.e1010 (2023). 37003256 10.1016/j.immuni.2023.03.002

[8] Smith, N. L. et al. Developmental origin governs CD8+ T cell fate decisions during infection. *Cell* **174**, 117–130.e114 (2018). 29909981 10.1016/j.cell.2018.05.029

[9] Cabeza-Cabrerizo, M., Cardoso, A., Minutti, C. M., Pereira da Costa, M. & Reis e Sousa, C. Dendritic cells revisited. *Annu. Rev. Immunol.* **39**, 131–166 (2021). 33481643 10.1146/annurev-immunol-061020-053707

[10] Guilliams, M. et al. Dendritic cells, monocytes and macrophages: a unified nomenclature based on ontogeny. *Nat. Rev. Immunol.* **14**, 571–578 (2014). 25033907 10.1038/nri3712 PMC4638219

[11] Hildner, K. et al. Batf3 deficiency reveals a critical role for CD8alpha+ dendritic cells in cytotoxic T cell immunity. *Science* **322**, 1097–1100 (2008). 19008445 10.1126/science.1164206 PMC2756611

[12] Mashayekhi, M. et al. CD8α(+) dendritic cells are the critical source of interleukin-12 that controls acute infection by Toxoplasma gondii tachyzoites. *Immunity* **35**, 249–259 (2011). 21867928 10.1016/j.immuni.2011.08.008 PMC3171793

[13] Lewis, K. L. et al. Notch2 receptor signaling controls functional differentiation of dendritic cells in the spleen and intestine. *Immunity* **35**, 780–791 (2011). 22018469 10.1016/j.immuni.2011.08.013 PMC3225703

[14] Liu, T. T. et al. Ablation of cDC2 development by triple mutations within the Zeb2 enhancer. *Nature* **607**, 142–148 (2022). 35732734 10.1038/s41586-022-04866-z PMC10358283

[15] Tussiwand, R. et al. Klf4 expression in conventional dendritic cells is required for T helper 2 cell responses. *Immunity* **42**, 916–928 (2015). 25992862 10.1016/j.immuni.2015.04.017 PMC4447135

[16] Briseño, C. G. et al. Notch2-dependent DC2s mediate splenic germinal center responses. *Proc. Natl. Acad. Sci. USA* **115**, 10726–10731 (2018). 30279176 10.1073/pnas.1809925115 PMC6196531

[17] Meiser, P. et al. A distinct stimulatory cDC1 subpopulation amplifies CD8(+) T cell responses in tumors for protective anti-cancer immunity. *Cancer Cell* **41**, 1498–1515.e1410 (2023). 37451271 10.1016/j.ccell.2023.06.008

[18] Bayerl, F. et al. Tumor-derived prostaglandin E2 programs cDC1 dysfunction to impair intratumoral orchestration of anti-cancer T cell responses. *Immunity* **56**, 1341–1358.e1311 (2023). 37315536 10.1016/j.immuni.2023.05.011

[19] Rivera, C. A. et al. Epithelial colonization by gut dendritic cells promotes their functional diversification. *Immunity* **55**, 129–144.e128 (2022). 34910930 10.1016/j.immuni.2021.11.008 PMC8751639

[20] Gargaro, M. et al. Indoleamine 2,3-dioxygenase 1 activation in mature cDC1 promotes tolerogenic education of inflammatory cDC2 via metabolic communication. *Immunity* **55**, 1032–1050.e1014 (2022). 35704993 10.1016/j.immuni.2022.05.013 PMC9220322

[21] Bosteels, V. & Janssens, S. Striking a balance: new perspectives on homeostatic dendritic cell maturation. *Nat. Rev. Immunol.* **25**, 125–140 (2024). 10.1038/s41577-024-01079-5 39289483

[22] Moon, C. Y. et al. Dendritic cell maturation in cancer. *Nat. Rev. Cancer* **25**, 225–248 (2025). 39920276 10.1038/s41568-024-00787-3 PMC11954679

[23] Eisenbarth, S. C. Dendritic cell subsets in T cell programming: location dictates function. *Nat. Rev. Immunol.* **19**, 89–103 (2019). 30464294 10.1038/s41577-018-0088-1 PMC7755085

[24] Ardouin, L. et al. Broad and largely concordant molecular changes characterize tolerogenic and immunogenic dendritic cell maturation in thymus and periphery. *Immunity* **45**, 305–318 (2016). 27533013 10.1016/j.immuni.2016.07.019

[25] Bosteels, V. et al. LXR signaling controls homeostatic dendritic cell maturation. *Sci. Immunol.* **8**, eadd3955 (2023). 37172103 10.1126/sciimmunol.add3955

[26] Ashby, K. M. et al. Sterile production of interferons in the thymus affects T cell repertoire selection. *Sci. Immunol.* **9**, eadp1139 (2024). 39058762 10.1126/sciimmunol.adp1139 PMC12052003

[27] Belkaid, Y. & Hand, T. W. Role of the microbiota in immunity and inflammation. *Cell* **157**, 121–141 (2014). 24679531 10.1016/j.cell.2014.03.011 PMC4056765

[28] Hornef, M. W. & Torow, N. Layered immunity’ and the ‘neonatal window of opportunity’ - timed succession of non-redundant phases to establish mucosal host-microbial homeostasis after birth. *Immunology* **159**, 15–25 (2020). 31777069 10.1111/imm.13149 PMC6904599

[29] Gollwitzer, E. S. et al. Lung microbiota promotes tolerance to allergens in neonates via PD-L1. *Nat. Med.* **20**, 642–647 (2014). 24813249 10.1038/nm.3568

[30] Papaioannou, N. E. et al. Environmental signals rather than layered ontogeny imprint the function of type 2 conventional dendritic cells in young and adult mice. *Nat. Commun.* **12**, 464 (2021). 33469015 10.1038/s41467-020-20659-2 PMC7815729

[31] Silva-Sanchez, A. et al. Activation of regulatory dendritic cells by Mertk coincides with a temporal wave of apoptosis in neonatal lungs. *Sci. Immunol.* **8**, eadc9081 (2023). 37327322 10.1126/sciimmunol.adc9081 PMC10351240

[32] Köhler, A., Delbauve, S., Smout, J., Torres, D. & Flamand, V. Very early-life exposure to microbiota-induced TNF drives the maturation of neonatal pre-cDC1. *Gut* **70**, 511–521 (2021). 32546472 10.1136/gutjnl-2019-319700

[33] Smout, J. et al. Maternal *Lactobacillus rhamnosus* administration impacts neonatal CD4 T-cell activation and prevents murine T helper 2-type allergic airways disease. *Front Immunol.* **13**, 1082648 (2022). 36685549 10.3389/fimmu.2022.1082648 PMC9847498

[34] Schaupp, L. et al. Microbiota-induced type I interferons instruct a poised basal state of dendritic cells. *Cell* **181**, 1080–1096.e1019 (2020). 32380006 10.1016/j.cell.2020.04.022

[35] de Kleer, I. M. et al. Perinatal activation of the interleukin-33 pathway promotes type 2 immunity in the developing lung. *Immunity* **45**, 1285–1298 (2016). 27939673 10.1016/j.immuni.2016.10.031

[36] Torres, D. et al. IL-12p40/IL-10 producing preCD8alpha/Clec9A+ dendritic cells are induced in neonates upon Listeria monocytogenes infection. *PLoS Pathog.* **12**, e1005561 (2016). 27074026 10.1371/journal.ppat.1005561 PMC4830566

[37] Torow, N. et al. M cell maturation and cDC activation determine the onset of adaptive immune priming in the neonatal Peyer’s patch. *Immunity* **56**, 1220–1238.e1227 (2023). 37130522 10.1016/j.immuni.2023.04.002 PMC10262694

[38] Sharma, P., Levy, O. & Dowling, D. J. The TLR5 agonist flagellin shapes phenotypical and functional activation of lung mucosal antigen presenting cells in neonatal mice. *Front. Immunol.* **11**, 171 (2020). 32132997 10.3389/fimmu.2020.00171 PMC7039933

[39] Sun, C. M., Fiette, L., Tanguy, M., Leclerc, C. & Lo-Man, R. Ontogeny and innate properties of neonatal dendritic cells. *Blood* **102**, 585–591 (2003). 12663436 10.1182/blood-2002-09-2966

[40] Narasimhan, H. et al. RORγt-expressing dendritic cells are functionally versatile and evolutionarily conserved antigen-presenting cells. *Proc. Natl. Acad. Sci. USA* **122**, e2417308122 (2025). 39993193 10.1073/pnas.2417308122 PMC11892598

[41] Zhang, Y. et al. Microbiota-mediated shaping of mouse spleen structure and immune function characterized by scRNA-seq and Stereo-seq. *J. Genet. Genomics* **50**, 688–701 (2023). 37156441 10.1016/j.jgg.2023.04.012

[42] Miller, J. C. et al. Deciphering the transcriptional network of the dendritic cell lineage. *Nat. Immunol.* **13**, 888–899 (2012). 22797772 10.1038/ni.2370 PMC3985403

[43] Liu, Z. et al. Dendritic cell type 3 arises from Ly6C(+) monocyte-dendritic cell progenitors. *Immunity* **56**, 1761–1777.e1766 (2023). 37506694 10.1016/j.immuni.2023.07.001

[44] Brown, C. C. et al. Transcriptional basis of mouse and human dendritic cell heterogeneity. *Cell* **179**, 846–863.e824 (2019). 31668803 10.1016/j.cell.2019.09.035 PMC6838684

[45] Sulczewski, F. B. et al. Transitional dendritic cells are distinct from conventional DC2 precursors and mediate proinflammatory antiviral responses. *Nat. Immunol.* **24**, 1265–1280 (2023). 37414907 10.1038/s41590-023-01545-7 PMC10683792

[46] Wang, J. et al. Single-cell multiomics defines tolerogenic extrathymic Aire-expressing populations with unique homology to thymic epithelium. *Sci. Immunol.* **6**, eabl5053 (2021). 34767455 10.1126/sciimmunol.abl5053 PMC8855935

[47] Hollox, E. J. & Louzada, S. Genetic variation of glycophorins and infectious disease. *Immunogenetics* **75**, 201–206 (2023). 36224278 10.1007/s00251-022-01280-7 PMC10205821

[48] Maier, B. et al. A conserved dendritic-cell regulatory program limits antitumour immunity. *Nature* **580**, 257–262 (2020). 32269339 10.1038/s41586-020-2134-y PMC7787191

[49] Ehlers, C., Schirmer, S., Kehlenbach, R. H., Hauber, J. & Chemnitz, J. Post-transcriptional regulation of CD83 expression by AUF1 proteins. *Nucleic Acids Res.* **41**, 206–219 (2013). 23161671 10.1093/nar/gks1069 PMC3592417

[50] Barrat, F. J., Crow, M. K. & Ivashkiv, L. B. Interferon target-gene expression and epigenomic signatures in health and disease. *Nat. Immunol.* **20**, 1574–1583 (2019). 31745335 10.1038/s41590-019-0466-2 PMC7024546

[51] de Mingo Pulido, A. et al. The inhibitory receptor TIM-3 limits activation of the cGAS-STING pathway in intra-tumoral dendritic cells by suppressing extracellular DNA uptake. *Immunity* **54**, 1154–1167.e1157 (2021). 33979578 10.1016/j.immuni.2021.04.019 PMC8192496

[52] Alspach, E., Lussier, D. M. & Schreiber, R. D. Interferon γ and its important roles in promoting and inhibiting spontaneous and therapeutic cancer immunity. *Cold Spring Harb. Perspect. Biol.* **11**, a028480 (2019). 29661791 10.1101/cshperspect.a028480 PMC6396335

[53] Lazear, H. M., Schoggins, J. W. & Diamond, M. S. Shared and distinct functions of type I and type III interferons. *Immunity* **50**, 907–923 (2019). 30995506 10.1016/j.immuni.2019.03.025 PMC6839410

[54] Caton, M. L., Smith-Raska, M. R. & Reizis, B. Notch-RBP-J signaling controls the homeostasis of CD8- dendritic cells in the spleen. *J. Exp. Med.* **204**, 1653–1664 (2007). 17591855 10.1084/jem.20062648 PMC2118632

[55] Schraml, B. U. et al. Genetic tracing via DNGR-1 expression history defines dendritic cells as a hematopoietic lineage. *Cell* **154**, 843–858 (2013). 23953115 10.1016/j.cell.2013.07.014

[56] Lutz, M. B., Backer, R. A. & Clausen, B. E. Revisiting current concepts on the tolerogenicity of steady-state dendritic cell subsets and their maturation stages. *J. Immunol.* **206**, 1681–1689 (2021). 33820829 10.4049/jimmunol.2001315

[57] Krajmalnik-Brown, R., Ilhan, Z. E., Kang, D. W. & DiBaise, J. K. Effects of gut microbes on nutrient absorption and energy regulation. *Nutr. Clin. Pr.* **27**, 201–214 (2012). 10.1177/0884533611436116 PMC3601187 22367888

[58] Rosshart, S. P. et al. Laboratory mice born to wild mice have natural microbiota and model human immune responses. *Science* **365**, eaaw4361 (2019). 31371577 10.1126/science.aaw4361 PMC7377314

[59] Lauvau, G. & Goriely, S. Memory CD8+ T cells: orchestrators and key players of innate immunity? *PLoS Pathog.* **12**, e1005722 (2016). 27584152 10.1371/journal.ppat.1005722 PMC5008753

[60] Jameson, S. C., Lee, Y. J. & Hogquist, K. A. Innate memory T cells. *Adv. Immunol.* **126**, 173–213 (2015). 25727290 10.1016/bs.ai.2014.12.001 PMC4670568

[61] White, J. T., Cross, E. W. & Kedl, R. M. Antigen-inexperienced memory CD8(+) T cells: where they come from and why we need them. *Nat. Rev. Immunol.* **17**, 391–400 (2017). 28480897 10.1038/nri.2017.34 PMC5569888

[62] Sosinowski, T. et al. CD8alpha+ dendritic cell trans presentation of IL-15 to naive CD8+ T cells produces antigen-inexperienced T cells in the periphery with memory phenotype and function. *J. Immunol.* **190**, 1936–1947 (2013). 23355737 10.4049/jimmunol.1203149 PMC3578102

[63] White, J. T. et al. Virtual memory T cells develop and mediate bystander protective immunity in an IL-15-dependent manner. *Nat. Commun.* **7**, 11291 (2016). 27097762 10.1038/ncomms11291 PMC4844673

[64] Kedl, R. M. & Mescher, M. F. Migration and activation of antigen-specific CD8+ T cells upon in vivo stimulation with allogeneic tumor. *J. Immunol.* **159**, 650–663 (1997). 9218580

[65] Martinet, V. et al. Type I interferons regulate eomesodermin expression and the development of unconventional memory CD8(+) T cells. *Nat. Commun.* **6**, 7089 (2015). 25953241 10.1038/ncomms8089 PMC4432629

[66] Salou, M. et al. A common transcriptomic program acquired in the thymus defines tissue residency of MAIT and NKT subsets. *J. Exp. Med.* **216**, 133–151 (2019). 30518599 10.1084/jem.20181483 PMC6314520

[67] Constantinides, M. G. et al. MAIT cells are imprinted by the microbiota in early life and promote tissue repair. *Science* **366**, eaax6624 (2019). 31649166 10.1126/science.aax6624 PMC7603427

[68] Sapoznikov, A. et al. Dendritic cell ICAM-1 strengthens synapses with CD8 T cells but is not required for their early differentiation. *Cell Rep.* **42**, 112864 (2023). 37494182 10.1016/j.celrep.2023.112864

[69] Nobs, E. et al. Cytosolic serpins act in a cytoprotective feedback loop that limits ESX-1-dependent death of *Mycobacterium marinum*-infected macrophages. *mBio* **15**, e0038424 (2024). 39087767 10.1128/mbio.00384-24 PMC11389378

[70] Medema, J. P. et al. Expression of the serpin serine protease inhibitor 6 protects dendritic cells from cytotoxic T lymphocyte-induced apoptosis: differential modulation by T helper type 1 and type 2 cells. *J. Exp. Med.* **194**, 657–667 (2001). 11535633 10.1084/jem.194.5.657 PMC2195949

[71] Solovey, M. et al. *Community* assesses differential cell communication using large multi-sample case-control scRNAseq datasets. Preprint at https://www.biorxiv.org/content/10.1101/2024.03.01.582941v1 (2024). 10.1016/j.isci.2026.117314 PMC13544351 42699773

[72] Bottcher, J. P. et al. Functional classification of memory CD8(+) T cells by CX3CR1 expression. *Nat. Commun.* **6**, 8306 (2015). 26404698 10.1038/ncomms9306 PMC4667439

[73] Hinrichs, A. C. et al. CCL5 release by CCR9+ CD8 T cells: a potential contributor to immunopathology of primary Sjögren’s syndrome. *Front. Immunol.* **13**, 887972 (2022). 35720379 10.3389/fimmu.2022.887972 PMC9198220

[74] Vatanen, T. et al. Mobile genetic elements from the maternal microbiome shape infant gut microbial assembly and metabolism. *Cell* **185**, 4921–4936.e4915 (2022). 36563663 10.1016/j.cell.2022.11.023 PMC9869402

[75] Goehring, K. C. et al. Similar to those who are breastfed, infants fed a formula containing 2’-fucosyllactose have lower inflammatory cytokines in a randomized controlled trial. *J. Nutr.* **146**, 2559–2566 (2016). 27798337 10.3945/jn.116.236919

[76] Mooser, C. et al. Diet-derived LPS determines intestinal IgA induction and repertoire characteristics independently of the microbiota. *Immunity* **58**, 1778–1793.e1777 (2025). 40578363 10.1016/j.immuni.2025.05.024

[77] Price, T. R. et al. Whole and isolated protein fractions differentially affect gastrointestinal integrity markers in C57Bl/6 mice fed diets with a moderate-fat content. *Nutrients* **13**, 1251 (2021). 33920187 10.3390/nu13041251 PMC8069602

[78] Chong, D. et al. Neonatal ketone body elevation regulates postnatal heart development by promoting cardiomyocyte mitochondrial maturation and metabolic reprogramming. *Cell Discov.* **8**, 106 (2022). 36220812 10.1038/s41421-022-00447-6 PMC9553951

[79] Görs, S., Kucia, M., Langhammer, M., Junghans, P. & Metges, C. C. Technical note: milk composition in mice-methodological aspects and effects of mouse strain and lactation day. *J. Dairy Sci.* **92**, 632–637 (2009). 19164675 10.3168/jds.2008-1563

[80] Garbow, J. R. et al. Hepatic steatosis, inflammation, and ER stress in mice maintained long term on a very low-carbohydrate ketogenic diet. *Am. J. Physiol. Gastrointest. Liver Physiol.* **300**, G956–G967 (2011). 21454445 10.1152/ajpgi.00539.2010 PMC3119109

[81] Alexandre, Y. O. et al. XCR1+ dendritic cells promote memory CD8+ T cell recall upon secondary infections with Listeria monocytogenes or certain viruses. *J. Exp. Med.* **213**, 75–92 (2016). 26694969 10.1084/jem.20142350 PMC4710197

[82] Bajénoff, M., Narni-Mancinelli, E., Brau, F. & Lauvau, G. Visualizing early splenic memory CD8+ T cells reactivation against intracellular bacteria in the mouse. *PLoS ONE* **5**, e11524 (2010). 20634957 10.1371/journal.pone.0011524 PMC2902518

[83] Bangs, D. J. et al. CXCR3 regulates stem and proliferative CD8+ T cells during chronic infection by promoting interactions with DCs in splenic bridging channels. *Cell Rep.* **38**, 110266 (2022). 35045305 10.1016/j.celrep.2021.110266 PMC8896093

[84] Dähling, S. et al. Type 1 conventional dendritic cells maintain and guide the differentiation of precursors of exhausted T cells in distinct cellular niches. *Immunity* **55**, 656–670.e658 (2022). 35366396 10.1016/j.immuni.2022.03.006

[85] Duckworth, B. C. et al. Effector and stem-like memory cell fates are imprinted in distinct lymph node niches directed by CXCR3 ligands. *Nat. Immunol.* **22**, 434–448 (2021). 33649580 10.1038/s41590-021-00878-5

[86] Ghilas, S. et al. Natural killer cells and dendritic epidermal gammadelta T cells orchestrate type 1 conventional DC spatiotemporal repositioning toward CD8(+) T cells. *iScience* **24**, 103059 (2021). 34568787 10.1016/j.isci.2021.103059 PMC8449251

[87] Piot, C. et al. Spatial organisation of tumour cDC1 states correlates with effector and stem-like CD8+ T cells location. *Eur. J. Immunol.* **55**, e70011 (2025). 40745918 10.1002/eji.70011 PMC12314343

[88] Erttmann, S. F. et al. The gut microbiota prime systemic antiviral immunity via the cGAS-STING-IFN-I axis. *Immunity* **55**, 847–861.e810 (2022). 35545033 10.1016/j.immuni.2022.04.006

[89] Woo, S. R. et al. STING-dependent cytosolic DNA sensing mediates innate immune recognition of immunogenic tumors. *Immunity* **41**, 830–842 (2014). 25517615 10.1016/j.immuni.2014.10.017 PMC4384884

[90] Ang, Q. Y. et al. Ketogenic diets alter the gut microbiome resulting in decreased intestinal Th17 cells. *Cell* **181**, 1263–1275.e1216 (2020). 32437658 10.1016/j.cell.2020.04.027 PMC7293577

[91] Collins, N. & Belkaid, Y. Control of immunity via nutritional interventions. *Immunity* **55**, 210–223 (2022). 35139351 10.1016/j.immuni.2022.01.004

[92] Dunbar, C. L. et al. Nutrition and immunity: perspectives on key issues and next steps. *Appl. Physiol. Nutr. Metab.* **48**, 484–497 (2023). 36888970 10.1139/apnm-2022-0276

[93] Hou, K. et al. Microbiota in health and diseases. *Signal Transduct. Target. Ther.* **7**, 135 (2022). 35461318 10.1038/s41392-022-00974-4 PMC9034083

[94] Lante, A., Canazza, E. & Tessari, P. Beta-glucans of cereals: functional and technological properties. *Nutrients* **15**, 2124 (2023). 37432266 10.3390/nu15092124 PMC10181044

[95] Ghoshal, S., Witta, J., Zhong, J., de Villiers, W. & Eckhardt, E. Chylomicrons promote intestinal absorption of lipopolysaccharides. *J. Lipid Res.* **50**, 90–97 (2009). 18815435 10.1194/jlr.M800156-JLR200

[96] Hrncir, T., Stepankova, R., Kozakova, H., Hudcovic, T. & Tlaskalova-Hogenova, H. Gut microbiota and lipopolysaccharide content of the diet influence development of regulatory T cells: studies in germ-free mice. *BMC Immunol.* **9**, 65 (2008). 18990206 10.1186/1471-2172-9-65 PMC2588440

[97] Yoshino, S., Sasatomi, E., Mori, Y. & Sagai, M. Oral administration of lipopolysaccharide exacerbates collagen-induced arthritis in mice1. *J. Immunol.* **163**, 3417–3422 (1999). 10477613

[98] He, X. et al. Lysine vitcylation is a vitamin C-derived protein modification that enhances STAT1-mediated immune response. *Cell* **188**, 1858–1877.e1821 (2025). 40023152 10.1016/j.cell.2025.01.043 PMC12308713

[99] Cerullo, G. et al. The long history of vitamin C: from prevention of the common cold to potential aid in the treatment of COVID-19. *Front. Immunol.* **11**, 574029 (2020). 33193359 10.3389/fimmu.2020.574029 PMC7655735

[100] Beura, L. K. et al. Normalizing the environment recapitulates adult human immune traits in laboratory mice. *Nature* **532**, 512–516 (2016). 27096360 10.1038/nature17655 PMC4871315

[101] Turner, S. J., Bennett, T. J. & La Gruta, N. L. CD8(+) T-cell memory: the why, the when, and the how. *Cold Spring Harb. Perspect. Biol.* **13**, a038661 (2021). 33648987 10.1101/cshperspect.a038661 PMC8091951

[102] Mowat, A. M. The regulation of immune responses to dietary protein antigens. *Immunol. Today* **8**, 93–98 (1987). 25289474 10.1016/0167-5699(87)90853-X

[103] Hoelting, T. L. B., Park, T. & Brown, C. C. Antigen-presenting cells as arbiters of mucosal tolerance and immunity. *Nat. Immunol.* **26**, 1890–1902 (2025). 10.1038/s41590-025-02320-6 41107526

[104] Rudnitsky, A. et al. A coordinated cellular network regulates tolerance to food. *Nature* **644**, 231–240 (2025). 10.1038/s41586-025-09173-x PMC12328219 40425043

[105] Zhou, Y. D. et al. T cell fate is dictated by different antigen presenting cells in response to dietary versus gut epithelial self-antigen. Preprint at https://www.biorxiv.org/content/10.1101/2025.11.06.687030v3 (2025). 10.1016/j.immuni.2026.07.023 PMC13519990 42648284

[106] This, S. et al. Type 1 and type 2 dendritic cell subsets cooperate to maintain intestinal immune tolerance via integrin αvβ8-mediated TGF-β activation. Preprint at https://www.biorxiv.org/content/10.64898/2026.02.26.707908v1v (2026).

[107] Chauveau, A. et al. Visualization of T cell migration in the spleen reveals a network of perivascular pathways that guide entry into T zones. *Immunity* **52**, 794–807.e797 (2020). 32298648 10.1016/j.immuni.2020.03.010 PMC7237890

[108] Kaushik, A. et al. CD8+ T cell differentiation status correlates with the feasibility of sustained unresponsiveness following oral immunotherapy. *Nat. Commun.* **13**, 6646 (2022). 36333296 10.1038/s41467-022-34222-8 PMC9636180

[109] Wallner, B. et al. Generation of mice with a conditional Stat1 null allele. *Transgenic Res.* **21**, 217–224 (2012). 21553074 10.1007/s11248-011-9519-5

[110] Yamamoto, M. et al. Role of adaptor TRIF in the MyD88-independent toll-like receptor signaling pathway. *Science* **301**, 640–643 (2003). 12855817 10.1126/science.1087262

[111] Probst, H. C. et al. Guidelines for DC preparation and flow cytometry analysis of mouse nonlymphoid tissues. *Eur. J. Immunol.* **53**, 2249819 (2023). 10.1002/eji.202249819 36512638

[112] Corbett, A. J. et al. T-cell activation by transitory neo-antigens derived from distinct microbial pathways. *Nature* **509**, 361–365 (2014). 24695216 10.1038/nature13160

[113] Fang, Z., Liu, X. & Peltz, G. GSEApy: a comprehensive package for performing gene set enrichment analysis in Python. *Bioinformatics* **39**, btac757 (2022). 10.1093/bioinformatics/btac757 PMC9805564 36426870

[114] Liu, D., Wu, J., An, J. & Cyster, J. G. Requirements for cDC2 positioning in blood-exposed regions of the neonatal and adult spleen. *J. Exp. Med.* **217**, e20192300 (2020). 32808016 10.1084/jem.20192300 PMC7596818

