# Supplementary material for: Peri-weaning, diet-induced activation of an IFNγ-mediated regulatory circuit promotes cDC1 maturation and CD8+ T cell differentiation
Source: Nat Commun. 2026 Aug 4;17:7631. doi: 10.1038/s41467-026-75853-5 (PMC13439377; doi:10.1038/s41467-026-75853-5)
Supplement: Supplementary file 1 [file 41467_2026_75853_MOESM15_ESM.pdf]

Corresponding author(s): Barbara U. Schraml
Last updated by author(s): 19.06.2026

Reporting Summary

Nature Portfolio wishes to improve the reproducibility of the work that we publish. This form provides structure for consistency and transparency in reporting. For further information on Nature Portfolio policies, see our Editorial Policies and the Editorial Policy Checklist.

Statistics

For all statistical analyses, confirm that the following items are present in the figure legend, table legend, main text, or Methods section.

n/a	Confirmed	
☐	☒	The exact sample size (*n*) for each experimental group/condition, given as a discrete number and unit of measurement
☐	☒	A statement on whether measurements were taken from distinct samples or whether the same sample was measured repeatedly
☐	☒	The statistical test(s) used AND whether they are one- or two-sided<br>*Only common tests should be described solely by name; describe more complex techniques in the Methods section.*
☒	☐	A description of all covariates tested
☐	☒	A description of any assumptions or corrections, such as tests of normality and adjustment for multiple comparisons
☐	☒	A full description of the statistical parameters including central tendency (e.g. means) or other basic estimates (e.g. regression coefficient) AND variation (e.g. standard deviation) or associated estimates of uncertainty (e.g. confidence intervals)
☐	☒	For null hypothesis testing, the test statistic (e.g. *F*, *t*, *r*) with confidence intervals, effect sizes, degrees of freedom and *P* value noted<br>*Give P values as exact values whenever suitable.*
☒	☐	For Bayesian analysis, information on the choice of priors and Markov chain Monte Carlo settings
☒	☐	For hierarchical and complex designs, identification of the appropriate level for tests and full reporting of outcomes
☒	☐	Estimates of effect sizes (e.g. Cohen's *d*, Pearson's *r*), indicating how they were calculated

Our web collection on statistics for biologists contains articles on many of the points above.

Software and code

Policy information about availability of computer code

Data collection	Flow cytometry data were collected on BD LSRFortessa with BD FACSDiva Software version 8.0.2. Cell sorting was performed using BD FACSAria Fusion.<br><br>10X Genomics Chromium Next GEM Single Cell 3’ kit v3 with Feature Barcode technology for Cell Surface Protein was used according to manufacturer's protocol was used to generate the scRNA-seq libraries of CD11c+MHCII+ cells from murine spleen across age. Agilent TapeStation was used for quantification and quality control steps when indicated in the protocol. Libraries were sequenced using NextSeq 1000.<br><br>10X Genomics Chromium Next GEM Single Cell 3’ Kit v3.1 (Dual Index) with Feature Barcode technology for Cell Surface Protein was used according to manufacturer’s protocol to generate the scRNA-seq libraries of CD11c+MHCII+ and CD90.2+ cells from murine spleen of Clec9a-cre Stat1-flox mice. Agilent TapeStation was used for quantification and quality control steps when indicated in the protocol. Libraries were sequenced using NextSeq 1000.

Data analysis	Flow cytometry data analysis was performed in FlowJo 10.8.1.<br><br>Flow cytometry data was compiled and analysed in GraphPad Prism 10.<br><br>scRNA-seq data from murine spleen was mapped using Cell Ranger v6.0.0, and analyzed using R v4.3.2 and Seurat versions 4.3.0 through 5.0.1.

For manuscripts utilizing custom algorithms or software that are central to the research but not yet described in published literature, software must be made available to editors and reviewers. We strongly encourage code deposition in a community repository (e.g. GitHub). See the Nature Portfolio guidelines for submitting code & software for further information.

Data

Policy information about availability of data

All manuscripts must include a data availability statement. This statement should provide the following information, where applicable:

- Accession codes, unique identifiers, or web links for publicly available datasets
- A description of any restrictions on data availability
- For clinical datasets or third party data, please ensure that the statement adheres to our policy

Single-cell RNA sequencing data have been deposited in the GEO repository under the accession number GSE337917. Data can be accessed via this link: https://www.ncbi.nlm.nih.gov/geo/query/acc.cgi?acc=GSE337917

Research involving human participants, their data, or biological material

Policy information about studies with human participants or human data. See also policy information about sex, gender (identity/presentation), and sexual orientation and race, ethnicity and racism.

Reporting on sex and gender	N/A
Reporting on race, ethnicity, or other socially relevant groupings	N/A
Population characteristics	N/A
Recruitment	N/A
Ethics oversight	N/A

Note that full information on the approval of the study protocol must also be provided in the manuscript.

Field-specific reporting

Please select the one below that is the best fit for your research. If you are not sure, read the appropriate sections before making your selection.

[x] Life sciences [ ] Behavioural & social sciences [ ] Ecological, evolutionary & environmental sciences

For a reference copy of the document with all sections, see nature.com/documents/nr-reporting-summary-flat.pdf

Life sciences study design

All studies must disclose on these points even when the disclosure is negative.

Sample size	No statistical tests were performed to predetermine sample size. Sample sizes for flow cytometry and functional assays were based on previous experiments, accounting for feasibility and availability of mice. Experiments where tissues from neonatal mice were pooled are indicated in the methods section or relevant figure legends. Sample sizes for single cell multiome and scRNAseq were chosen to have two or more biological replicates as recommended for high-throughput sequencing approaches. Sample size for each experiment is indicated in the legend.
Data exclusions	Low quality cells, doublets and untagged cells were filtered out where applicable from scRNAseq analyses as described in the methods section.
Replication	All experiments were performed with multiple biologically independent samples. Number of independent samples (n) and number of independent repetitions for each experiment are indicated in the legend. All experiments, except scRNAseq were repeated at least two times to ensure findings are reproducible.
Randomization	Where relevant, co-housed mice were age and sex matched and allocated to experimental groups based on genotyping results. Littermates were used in experiments, wherever possible.
Blinding	Investigators were not blinded as experiments were planned to ensure age or genotypes of mice as required.

Reporting for specific materials, systems and methods

We require information from authors about some types of materials, experimental systems and methods used in many studies. Here, indicate whether each material, system or method listed is relevant to your study. If you are not sure if a list item applies to your research, read the appropriate section before selecting a response.

Materials & experimental systems

n/a	Involved in the study
☐	☒ Antibodies
☒	☐ Eukaryotic cell lines
☒	☐ Palaeontology and archaeology
☐	☒ Animals and other organisms
☒	☐ Clinical data
☒	☐ Dual use research of concern
☒	☐ Plants

Methods

n/a	Involved in the study
☒	☐ ChIP-seq
☐	☒ Flow cytometry
☒	☐ MRI-based neuroimaging

Antibodies

Antibodies used

All antibody details are listed in Supplementary Table 4 and also listed below:
Antibody used for flow cytometry:
anti-CD3ε-FITC 145-2C11 Biolegend 100306
anti-CD3ε-PE-Cy5 145-2C11 Biolegend 100310
anti-CD3ε-AF647 145-2C11 Biolegend 100322
anti-CD4 APC GK1.5 BioLegend 100412
anti-CD4-BUV395 RM4-5 BD Biosciences 740208
anti-CD4-BUV737 GK1.5 BD Biosciences 612761
anti-CD8a-BV605 53-6.7 Biolegend 100744
anti-CD8a-FITC 53-6.7 BD Biosciences 553031
anti-CD11b-BV421 M1/70 Biolegend 101236
anti-CD11b-BV605 M1/70 Biolegend 101237
anti-CD11b-BUV737 M1/70 BD Biosciences 612800
anti-CD11c-BV785 N418 Biolegend 117336
anti-CD11c-PE N418 Biolegend 117308
anti-CD16/32 purified 2.4G2 BD Biosciences 553142
anti-CD19-BV650 6D5 Biolegend 115541
anti-CD19-FITC 6D5 Biolegend 115506
anti-CD24-FITC M1/69 Biolegend 101806
anti-CD24-BUV395 M1/69 BD Biosciences 744471
anti-CD25-BV785 PC61 Biolegend 102051
anti-CD44-BV650 IM7 BD Biosciences 740455
anti-CD44-PB IM7 BioLegend 103019
anti-CD45.1-FITC A20 BioLegend 110706
anti-CD45.2-AF700 104 BioLegend 109822
anti-CD45.2-FITC 104 BioLegend 109806
anti-CD45.2-PB 104 BioLegend 109820
anti-CD45R/B220-FITC RA3-6B2 Biolegend 103206
anti-CD45R/B220-PE RA3-6B2 Biolegend 103208
anti-CD45R/B220-PB RA3-6B2 Biolegend 103227
anti-CD45R/B220-AF647 RA3-6B2 Biolegend 103226
anti-CD49d-APC R1-2 Biolegend 103621
anti-CD49d-FITC R1-2 Biolegend 103605
anti-CD62L PE-Cy7 MEL-14 Tonbo Bioscience 60-0621-U100
anti-CD83-PE Michel-19 Biolegend 121508
anti-CD83-APC Michel-19 Biolegend 121509
anti-CD86-BV605 GL-1 Biolegend 105037
anti-CD90.2-AF700 30-H12 Biolegend 105320
anti-CD90.2-PE-Cy7 30-H12 Biolegend 105326
anti-CD107a (LAMP1)-BV421 1D4B Biolegend 121617
anti-CD161c/NK1.1-PE-Cy5 PK136 Biolegend 108716
anti-CD183 (CXCR3)-APC Cxcr3-173 Biolegend 126511
anti-F4/80-BV605 BM8 Biolegend 123133
anti-F4/80-BV785 BM8 Biolegend 123141
anti-F4/80-AF647 BM8 Biolegend 123122
anti-MHCII I-A/I-E-AF700 M5/114.15.2 Biolegend 107622

anti-MHCII I-A/I-E-PB M5/114.15.2 Biolegend 107620
anti-Ly6G-FITC 1A8 Biolegend 127606
anti-XCR1-BV421 ZET Biolegend 148216
anti-XCR1-BV650 ZET Biolegend 148220
anti-V-alpha2 TCR-BUV395 B20.1 BD Biosciences 743834
anti-TCRβ chain-BV785 H57-597 Biolegend 109249
anti-γδ T-Cell Receptor-BUV395 GL3 BD Biosciences 744118
anti-TER-119-FITC TER-119 Biolegend 116206
anti-ESAM PE-Cy7 1G8/ESAM Biolegend 136211
anti-ESAM APC 1G8/ESAM Biolegend 136207
anti-Siglec H eFluor660 eBio440c eBiosience 50-0333-82
anti-Foxp3-AF647 150D Biolegend 320014
anti-RORγt-BV421 Q31-378 BD Biosciences 562894
anti-IFN-γ-APC XMG1.2 Biolegend 505810
anti-IFN-γ-BV650 XMG1.2 Biolegend 505831
anti-IL17A-FITC TC11-18H10.1 Biolegend 506907
anti-IL-4-PE 11B11 Biolegend 504104
anti-TNFa-PECy7 MP6-XT22 Biolegend 506324
anti-IL-2-BV421 JES6-5H4 Biolegend 503825
anti-IRF8-PerCP-eFluor710 V3GYWCH eBioscience 12-9852-82
anti-EOMES-PE W17001A Biolegend 157705
anti-CXCL9 (MIG)-PE MIG-2F5.5 Biolegend 515603
anti-Granzyme B-FITC QA16A02 Biolegend 372206
anti-Granzyme B-PE QA16A02 Biolegend 372207
anti-IL-12/IL-23 p40-PE-Cy7 C15.6 Biolegend 505210

Antibodies used for scRNAseq experiments:
TotalSeq-B0305 anti-mouse Hashtag 5 (anti-MHCI, anti-CD45) M1/42; 30-F11 Biolegend 155839
TotalSeq-B0310 anti-mouse Hashtag 10 (anti-MHCI, anti-CD45) M1/42; 30-F11 Biolegend 155849

Antibodies used for in vitro stimulation
anti-CD3ε Ultra-LEAF™ Purified 145-2C11 Biolegend 100340
anti-CD28 Ultra-LEAF™ Purified 37.51 Biolegend 102116

Following tetramers (Corbett et al., 2014, Nature) were used to identify MAIT cells in the spleen: Tetramers were gifts from Katrin Böttcher.
MR1-5-OP-RU-BV421
MR1-6-FP-BV421

Validation

All antibodies used here were validated by the manufacturer and/or in previously published studies. All antibodies were titrated on murine splenocytes to determine optimal dilution for flow cytometry analyses in our laboratory. For MAIT cell tetramer staining, the protocol from NIH Tetramer Core Facility was used.

Animals and other research organisms

Policy information about studies involving animals; ARRIVE guidelines recommended for reporting animal research, and Sex and Gender in Research

Laboratory animals

Clec9atm2.1(icre)Crs (Clec9aCre) (Jackson Laboratory Stock No: 025523), Gt(ROSA)26Sortm9(CAG-tdTomato)Hze (Rosa26lox-STOP-lox-tdtomato) (Jackson Laboratory Stock No: 007909), Ifnar1tm1Agt (Ifnar−/−) (MMRRC Stock No: 32045-JAX), B6(Cg)-Ifnar1tm1(flox)Uka (Ifnar1flox), Tg(Itgax-cre)1-1Reiz (CD11c-cre) (Jackson Laboratory Stock No: 018967), Stat1tm1.1Mmul (Stat1flox) , C57BL/6-Tg(Nr4a1-EGFP/cre)820Khog-Ptprca (Nr4a1eGFP) (Jackson Laboratory Stock No: 016617) , OTI mice (C57BL/6-Tg(TcraTcrb)1100Mjb/J, Jackson Laboratory stock no: 003831), C57BL/6JRccHsd (RRID:IMSR_ENV:HSD-043) mice were bred and maintained at the Biomedical Center, LMU, Munich. B6.129S7-Ifngr1tm1Agt/J (Ifngr1-/-) (Jackson Laboratory Stock No: 003288) mice were a gift from Sophie Janssens and bred and maintained at the Ghent University, Belgium. C57BL/6J (Jackson Laboratory stock no: 000664) wildlings were created through inverse germ-free rederivation as described (Rosshart, et al., 2019, Science).The wildling mouse colony was housed and animals were bred at the Medical Center – University of Freiburg, Germany. Germ free animals were provided by Dirk Haller, bred at the ZIEL institute for Food & Health – TUM, Freising, Germany in isolators, and germ-free status was routinely monitored. Germ-free Myd88-/-TrifLPS2/LPS2 (Yamamato et al., 2003) mice were bred and maintained in flexible-film isolators at the Clean Mouse Facility, University of Bern, Switzerland. Mice of various ages were used in this study as indicated in the figures and legends. Mice over the age of 8 weeks were considered adults. Unless otherwise stated, both male and female mice were used in this study.

Wild animals

No wild animals were used in this study.

Reporting on sex

Both male and female mice were used in this study. For ketogenic diet experiment with adult mice, only female mice were used to allow for stable group-housing.

Field-collected samples	No field-collected samples were used in this study.
Ethics oversight	All animal procedures were performed in accordance with national and institutional guidelines for animal welfare and approved by Regierung von Oberbayern, Regierungspräsidium Freiburg, the Cantonal Commissions for Animal Experimentation of the Cantone of Berne and VIB site Ghent–Ghent University Faculty of Science.

Note that full information on the approval of the study protocol must also be provided in the manuscript.

Plants

Seed stocks	N/A
Novel plant genotypes	N/A
Authentication	N/A

Flow Cytometry

Plots

Confirm that:

- [ ] The axis labels state the marker and fluorochrome used (e.g. CD4-FITC).
- [ ] The axis scales are clearly visible. Include numbers along axes only for bottom left plot of group (a 'group' is an analysis of identical markers).
- [x] All plots are contour plots with outliers or pseudocolor plots.
- [x] A numerical value for number of cells or percentage (with statistics) is provided.

Methodology

Sample preparation	Detailed protocols used to prepare single cell suspensions of different tissues, as well as protocols for enrichment and staining of cells for sorting are provided in the Methods section.
Instrument	BD LSRFortessa and BD FACSAria Fusion.
Software	Data was collected with BD FACSDiva Software version 8.0.2. Data was analyzed with FlowJo software version 10.8.1 (Tree Star, Inc.).
Cell population abundance	Frequencies of analyzed cell populations are indicated in representative plots.<br>Sequential gating strategy and purity of sorted samples are shown in representative plots in the Supplementary Figure 1a,b.<br>Cells were quantified by using CountBright™ Absolute Counting Beads (Thermo Fisher Scientific).
Gating strategy	All samples were initially gated on FSC-A vs SSC-A plot, doublet exclusion was performed using FSC-A vs FSC-H and FSC-A vs SSCW plots. Live cells were gated based on selecting cells negative for Fixable Viability Dye eFluor 780 (Thermo Fisher Scientific).<br>For DCs: F4/80^{hi}Autofluorescent-high red pulp macrophages (RPMs) were excluded, and CD11c+ MHCII+ cells were identified. Within this population, CD24+XCR1+ cDC1, CD24+XCR1neg (tDC) and CD11b+ cDC2 were identified. Within the CD11b+ cDC2 population, ESAM expression was used to further distinguish ESAM-high DC2 and ESAM-low cDC2.<br>For T cells and NK cells: After excluding dead cells and Autofulorescence-high cells, NK cells (NK1.1+CD3eneg), NKT cells (NK1.1+CD3e+), T cells (NK1.1negCD3e+) were identified. T cells were further divided into CD8+ and CD4+ T cells.<br><br>Cell populations were identified with gating strategies shown in Supplementary Figure 1a,b, Supplementary Figure 2h, Supplementary Figure 3a, and Supplementary Figure 6a.<br>Sequential gating strategy is shown Supplementary Figure 1a.<br>Representative sort purity is shown in Supplementary Figure 1b.

- [x] Tick this box to confirm that a figure exemplifying the gating strategy is provided in the Supplementary Information.
