# Supplementary material for: Peri-weaning, diet-induced activation of an IFNγ-mediated regulatory circuit promotes cDC1 maturation and CD8+ T cell differentiation
Source: Nat Commun. 2026 Aug 4;17:7631. doi: 10.1038/s41467-026-75853-5 (PMC13439377; doi:10.1038/s41467-026-75853-5)
Supplement: Supplementary file 2 [file 41467_2026_75853_MOESM16_ESM.pdf]

nature portfolio

Peer Review File

Peri-weaning, diet-induced activation of an IFNγ-mediated regulatory circuit promotes cDC1 maturation and CD8⁺ T cell differentiation

Corresponding Author: Professor Barbara Schraml

This manuscript has been previously reviewed at another journal. This document only contains information relating to versions considered at Nature Communications.

This file contains all reviewer reports in order by version, followed by all author rebuttals in order by version.

Version 1:

Reviewer comments:

Reviewer #1

(Remarks to the Author)
I thank the authors for their detailed replies to the original comments and for providing additional material. These have addressed and clarified several points, although some important gaps remain, including what component of the diet is driving the initial production of gIFN, where this occurs, where these lymphocytes and the CXCL9 producing cDC1 interact and whether the weaning-induced phenomenon is of relevance to eg protective immunity later in life. For me, the absence of definitive answers to these aspects, together with the rather mild phenotype of several of the experimental manipulations continue to detract from the overall impact of the paper. Specific comments:
1) The fact that cDC1 do not appear to express TLR2, 3 or 9, despite the overall phenomenon being dependent on TLR signalling brings the focus back to which cell is responsible for producing gIFN in response to the diet. While the data presented suggest that CD8+ T cells are a major source, their role is not proved directly and it remains unclear if this involves specific antigen, or some kind of innate-like response to dietary contaminants. Without conditional deletion of the potential source(s), it is not appropriate to conclude that the study "shows that IFNg produced from lymphocytes in response to dietary components present in chow drives cDC1 into an immunogenic maturation" as the authors do.
2) I continue to find the analyses based on expression of IRF8, CD83 and NR4A1 very confusing and unsure of how these add to the study. In particular, it seems that the protein expression patterns do not follow those suggested by the transcriptional data and it is difficult to understand how eg the IRF8lo/IRFneg populations fit with the RNA expression patterns. It is also notable that although it is stated that CD83+ cells represent a more mature state of cDC1, the most abundant population at 1 week of age appears to be CD83+.
3) Why are the baseline proportions of cDC1 expressing CXCL9 so different in the control groups shown for the various experiments?
4) The new information showing that CXCL9+ cDC1 appear to be in a bloodstream accessible compartment in the spleen, whereas CD3+ T cells are extravascular, makes it even more difficult to understand how these cells can be interacting with each other in a biologically relevant manner, as suggested by the Community analysis. A concern that this approach needs to be confirmed anatomically is underlined by the fact that it also predicted interaction between the CCR7+ cDC1 and CD8+ T cells which the authors do not follow up on anatomical grounds.
5) Despite the authors' response to my original comments, I wonder if these issues might have been simplified if the target tissue of the intestine or its lymphoid tissues had been examined. The potential relevance of this is highlighted by the authors' statement that they believe the Stat1 dependent CD8+ Tem are not primed initially in the spleen, but are attracted there by the CXCL9+ cDC1. Identification of this site and how the T cells arrive in the spleen would be important additions to the study.
5) What are clusters 0 and 3 revealed by the reclusterng shown in Supplementary Figure 7f, but not discussed? These appear to be of some significance, as cluster 0 seems to be the one which expands most at 3 weeks, while cluster 3 decreases markedly at this point.
6) While of some interest, the experiments using OT1 cells do not really address the issue raised by both reviewers about the functional consequences of the weaning effect on cDC1 for eg infections. As it is not at all clear what the relevance of CD8+ T cell responses to food proteins is, interpreting these experiments is difficult. It would also be important to know if the

chow used contains ovalbumin.
7) The lack of identification of the dietary component driving the initial gIFN response remains an important defect. For instance, it would be important to know the levels of endotoxin contamination in the two diets.For these reasons, it is not clear how to interpret the experiments comparing the ketogenic and standard purified diets, as it seems the main difference between them is their metabolic composition, which does not seem to be directly relevant to the other properties of the diet that are discussed. Interpretation is also clouded by the discordance in their effects, with only the ketogenic diet influencing the number of XCR1+ DC, while both diets affect the expression of CXCL9 and IL12p40.

Reviewer #2

(Remarks to the Author)
To the best of my appreciation, the authors have satisfactorily answered most of the points and concerns from all reviewers.

They have performed many new bench experiments, with the generation of additional novel high-quality data complementing the initial results present in the first version of the manuscript.

They have also edited some of their interpretations or conclusions, to make them more precise or to nuance them, accordingly to the recommendations from the reviewers.

Altogether, these changes have further improved the quality of the manuscript, in a manner that convincingly demonstrates the originality and potential importance of the discovery of the specific activation state imprinted on splenic cDC1s by the diet change at the time of weaning of the mice.

I have only identified a minor point left, which pertains to the functions of the specific activation state of cDC1s identified by the authors.

To the best of my understanding, the authors have at least partly changed their hypothesis between the initial manuscript and its revised version from (i) an immunogenic cDC1 activation state poised for promoting protective responses to infectious agents, to (ii) a possible role in tolerance induction to microbial or food antigens. Yet, this is not entirely clear and I am confused by the discussion on this issue.

In particular, the specific hypothesis is not clear to me behind the sentence “our data suggest that the immune system systemically relays dietary cues during weaning, allowing it to tailor the T cell response against environmental antigens to the need of the developing organism, striking a balance between effector immunity and T cell tolerance”.

On the one hand, the authors state that the cDC1 activation state they identify is immunogenic. “cDC intrinsic loss of Stat1 reduces cytotoxic CD8+ TEM cells in spleen, suggesting that the homeostatic maturation of cDC1 is not exclusively tolerogenic”, “our study identifies a previously unrecognized, microbiota-independent regulatory circuit by which dietary cues are relayed through IFNγ to imprint spleen-resident cDC1 with an immunogenic maturation program” and “these findings redefine the landscape of steady-state cDC1 maturation, challenging the prevailing view that homeostatic cDC1 maturation primarily promotes tolerance”.

On the other hand, they write that the CD8+ T cell response promoted by these cDC1s is associated to tolerance to food or microbial antigens. “Steady state CD49d+CD8+ TEM cells likely recognize environmental or food-derived antigens. […] Antigens encountered via the diet normally induce T cell tolerance, although such tolerance mechanisms can be suspended depending on the context in which dietary antigens are sampled. […] food-antigen specific CD8+ T cells were more cytotoxic in spleens of weanling mice on chow compared to milk diet. Effective desensitization against food allergens by oral immunotherapy has been linked to an increased frequency of cytotoxic CD8+ TEM cells, specifically in peripheral blood of patients with sustained unresponsiveness over those with recurring”.

I do not understand what hypothesis the authors privilege in the end about the function of the cDC1 activation state imprinted on splenic cDC1s by the diet change at the time of weaning of the mice. Is-it contributing to tolerance to food or microbial antigens, or poised to promote protective responses to infectious agents?

I understand that addressing it experimentally is beyond the scope of the present study and not necessary for publication, considering the originality and mechanistic dissection of the reported observation.

However, I think that, in the discussion of the manuscript, the perspectives open from the current work require further clarifications, including regarding the hypotheses proposed on the function of the cDC1 activation state discovered and on how to test them experimentally in future studies.
In this regard, the authors should cite and discuss the following preprint about the role of cDC1s in intestinal tolerance (This S … Paidassi H. Type 1 and type 2 dendritic cell subsets cooperate to maintain intestinal immune tolerance via integrin αvβ8-mediated TGF-β activation. bioRxiv 2026. doi: https://doi.org/10.64898/2026.02.26.707908).

Open Access This Peer Review File is licensed under a Creative Commons Attribution 4.0 International License, which permits use, sharing, adaptation, distribution and reproduction in any medium or format, as long as you give appropriate credit to the original author(s) and the source, provide a link to the Creative Commons license, and indicate if changes were made.
In cases where reviewers are anonymous, credit should be given to 'Anonymous Referee' and the source.
The images or other third party material in this Peer Review File are included in the article's Creative Commons license, unless indicated otherwise in a credit line to the material. If material is not included in the article's Creative Commons license and your intended use is not permitted by statutory regulation or exceeds the permitted use, you will need to obtain permission directly from the copyright holder.
To view a copy of this license, visit https://creativecommons.org/licenses/by/4.0/

Reviewer #1 (Remarks to the Author):

I thank the authors for their detailed replies to the original comments and for providing additional material. These have addressed and clarified several points, although some important gaps remain, including what component of the diet is driving the initial production of gIFN, where this occurs, where these lymphocytes and the CXCL9 producing cDC1 interact and whether the weaning-induced phenomenon is of relevance to eg protective immunity later in life. For me, the absence of definitive answers to these aspects, together with the rather mild phenotype of several of the experimental manipulations continue to detract from the overall impact of the paper. Specific comments:

1) The fact that cDC1 do not appear to express TLR2, 3 or 9, despite the overall phenomenon being dependent on TLR signalling brings the focus back to which cell is responsible for producing gIFN in response to the diet. While the data presented suggest that CD8+ T cells are a major source, their role is not proved directly and it remains unclear if this involves specific antigen, or some kind of innate-like response to dietary contaminants. Without conditional deletion of the potential source(s), it is not appropriate to conclude that the study "shows that IFNg produced from lymphocytes in response to dietary components present in chow drives cDC1 into an immunogenic maturation" as the authors do.

We show that IFNγ in spleen is predominately produced from lymphocytes: already at two weeks of age about 70% of all IFNγ-positive cells are lymphocytes and from 3 weeks of age onwards this percentage exceeds 90% (Fig. 3b-d). These data support the conclusion that IFNγ produced by lymphocytes drives the cDC1 into the here described CXCL9^{+} activation state. Identifying the specific cellular source would be beyond the scope of the current study, which focuses on establishing a link between diet and the CXCL9 producing cDC1 in spleen that is independent of the microbiota. Since we do not know if the intial trigger for IFNγ production involves specific antigens or and an innate-like response, we have thus reworded the above statement to more accurately reflect our findings: “We show that dietary components in chow trigger a Myd88/TRIF-dependent immune response independent of the microbiota. This response is relayed to the spleen via IFNγ production from lymphocytes, which equips cDC1 immunostimulatory functions that shape the effector differentiation of antigen-experienced cytotoxic CD8^{+} effector T cells during weaning”. (See page 14 of the revised manuscript).

2) I continue to find the analyses based on expression of IRF8, CD83 and NR4A1 very confusing and unsure of how these add to the study. In particular, it seems that the protein expression patterns do not follow those suggested by the transcriptional data and it is difficult to understand how eg the IRF8lo/IRFneg populations fit with the RNA expression patterns. It is also notable that although it is stated that CD83+ cells represent a more mature state of cDC1, the most abundant population at 1 week of age appears to be CD83+.

These experiments serve as an important validation step. They show that the identified transcriptional heterogeneity can be confirmed using protein staining and is related to maturation states based on differential expression of co-stimulatory molecules. We have explicitly stated this on page 7 of the revised manuscript.

We believe that the reviewer is referring to cluster 0 (CD83^{+}IRF8^{low}) of the scRNA-seq data in Fig. 2 being more abundant at week 1 transcriptionally. Changes in the relative abundance of scRNA-seq clusters across age must be interpreted with caution: The scRNA-seq cluster analyses in Fig. 2c excludes clusters comprising of proliferating cells, well as migratory DC clusters. In flow cytometry, however, markers for proliferating cells and CCR7^{+} migratory cDC1 were not used, providing one possible explanation why transcriptionally cluster 0 appears more abundant at week 1 compared to later time points, while in flow cytometry the frequency of CD83 expressing cDC1 is relatively constant over time or increases slightly with age. Additionally, CD83 can be post-transcriptionally regulated (PMID: 23161671), which could also contribute to CD83^{+} cells being more frequent in transcriptional analyses, which is now stated on page 7 of the revised manuscript.

3) Why are the baseline proportions of cDC1 expressing CXCL9 so different in the control groups shown for the various experiments?

The fact that the baseline frequency of CXCL9^{+} cDC1 varies somewhat between experiments is likely due to a combination of technical and biological factors. i) Different experimental cohorts were processed independently. ii) In some cases distinct sample preparation protocols or reagents of different lots were used when experiments were performed in collaborating labs. For instance, in wildling experiments cells were isolated by mechanical disruption, rather than enzymatic digestion (this has been specified in the revised methods section). iii) Animals were derived from different facilities and experimental cohorts, which can contribute to variation in baseline immune activation states. iv) Importantly, we occasionally observe modest differences in baseline CXCL9 expression between independent repeat experiments even when using the same protocol, suggesting that some degree of technical and/or biological variability is expected. Importantly, despite these variations, our interpretation was always guided based on comparisons made within individual experiments, where samples were processed in parallel and

analyzed using identical conditions and experimental controls. Under these controlled conditions, the observed differences between experimental groups were reproducible and consistent across independent experiments.

4) The new information showing that CXCL9+ cDC1 appear to be in a bloodstream accessible compartment in the spleen, whereas CD3+ T cells are extravascular, makes it even more difficult to understand how these cells can be interacting with each other in a biologically relevant manner, as suggested by the Community analysis. A concern that this approach needs to be confirmed anatomically is underlined by the fact that it also predicted interaction between the CCR7+ cDC1 and CD8+ T cells which the authors do not follow up on anatomical grounds.

We thank the reviewer for pointing this out. We would like to clarify that the here identified CXCL9$^+$ cDC1 state lacks CCR7 expression (Fig. 6c). In i.v. labelling experiments we chose total CD3$^+$ cells – including both CD4$^+$ and CD8$^+$ T cells - as control because the majority of all T cells in spleen are located in the white pulp, evident by their relatively low labeling in Supp. Fig. 6f. However, histologically, both CD4$^+$ and CD8$^+$ T cells are also found in the red pulp area and bridging channels: As the spleen lacks afferent lymphatics, all cells, including T cells, enter the spleen via the blood and T cells are released from arterioles into the red pulp sinuses in a CCR7-independent manner (PMID: 32298648). As such, localization of CXCL9$^+$ cDC1 in blood exposed regions conceivably enables their interaction with incoming CD8$^+$ T cells. We have now empirically addressed this point in the discussion on page 17 of the revised manuscript.

5) Despite the authors' response to my original comments, I wonder if these issues might have been simplified if the target tissue of the intestine or its lymphoid tissues had been examined. The potential relevance of this is highlighted by the authors' statement that they believe the Stat1 dependent CD8+ Tem are not primed initially in the spleen, but are attracted there by the CXCL9+ cDC1. Identification of this site and how the T cells arrive in the spleen would be important additions to the study.

As pointed out under point 4 above all T cells enter the spleen via the blood and are released from arterioles into the red pulp sinuses. This point of entry could facilitate the interaction with CXCL9$^+$ cDC1 in blood exposed regions of the spleen. We now realize that our wording "are attracted… to spleen niches" could imply that CXCL9$^+$ cDC1 actively recruit CD8$^+$ T cells from the periphery to the spleen. However, we favor the interpretation that CXCL9$^+$ cDC1 can interact with any memory CD8$^+$ T cell that reaches the red pulp, either directly from the circulation or after having been primed in the spleen. We have clarified this point in the discussion on page 17 of the revised manuscript as follows: We propose that IFNγ-stimulated CXCL9$^+$ cDC1 shape these antigen-experienced CD8$^+$ T cells by providing additional tissue-specific cues that reinforce or modify the functional state of these CD8$^+$ T cells after their initial priming in the intestine or elsewhere. Thus, rather than determining whether dietary or other environmental antigens elicit tolerance or immunity, this regulatory circuit may allow the immune system to systemically tune the effector state of antigen-experienced CD8$^+$ T cells according to dietary context in a feed-forward manner.

5) What are clusters 0 and 3 revealed by the reclusterng shown in Supplementary Figure 7f, but not discussed? These appear to be of some significance, as cluster 0 seems to be the one which expands most at 3 weeks, while cluster 3 decreases markedly at this point.

Suppl. Fig. 7a, b shows the reclustered UMAP using the original annotations from Fig. 2b in panel (a) and new cluster annotations in panel (b). Comparing these two UMAPs shows that new cluster 0 is contained within the original cluster 1_DC1, while new cluster 3 is largely contained within the original cluster 0_DC1. As such, these data are consistent with our analyses in Fig. 2b, in which cluster 1_DC1 expands most at 3 weeks, and cluster 0_DC1 is most abundant at week 1. We have now explained this in the manuscript and clarified that the reclustering analyses served the purpose to show that the CXCL9$^+$ cDC1 state was initially contained within a larger cluster in the scRNA-seq data across age but can be resolved when using a higher resolution.

6) While of some interest, the experiments using OT1 cells do not really address the issue raised by both reviewers about the functional consequences of the weaning effect on cDC1 for eg infections. As it is not at all clear what the relevance of CD8+ T cell responses to food proteins is, interpreting these experiments is difficult. It would also be important to know if the chow used contains ovalbumin.

We had previously outlined the limitations of infection experiments suggested by the reviewers to test the relevance of the here identified diet IFNγ-mediated regulatory circuit and would like to refer the reviewer to our first rebuttal.

We shared the concern of the reviewer regarding OVA contamination of the chow. Altromin chow – used in the OVA feeding experiments – is free of animal origin and should thus not contain ovalbumin. To confirm this, we gavaged mice with PBS as control. As shown in Supp. Fig. 9d OTI T cells expanded specifically when mice were gavaged with OVA, but not with PBS, supporting that chow or formula milk are a negligible source of OVA and do not affect our results.

7) The lack of identification of the dietary component driving the initial gIFN response remains an important defect. For instance, it would be important to know the levels of endotoxin contamination in the two diets.For these reasons, it is not clear how to interpret the experiments comparing the ketogenic and standard purified diets, as it seems the main difference between them is their metabolic composition, which does not seem to be directly relevant to the other properties of the diet that are discussed. Interpretation is also clouded by the discordance in their effects, with only the ketogenic diet influencing the number of XCR1+ DC, while both diets affect the expression of CXCL9 and IL12p40.

We agree with the reviewer that it would be interesting to know the endotoxin levels of the diets used in the context of prior work showing LPS contamination in diet can cause immune activation (PMID: 40578363). Purified diets are manufactured using refined, specific ingredients than whole agricultural products and their LPS levels are generally lower and much more controlled than standard, unrefined rodent chows (PMID: 18990206). Knowing the levels of LPS contamination would not alter our conclusion that Myd88-TRIF activating bioactive dietary components initiate the here identified IFNγ-mediated regulatory circuit, although we cannot exclude that mechanical forces related to dietary consistency trigger Myd88-TRIF dependent immune activation. We believe that under SPF conditions endotoxins derived from microbiota would outnumber minute traces present in chow and consider it more likely that plant derived products, such as beta glucans are involved.

Breast milk, formula diet or purified mouse diets (including ketogenic and standard purified diets) lack the above-mentioned heterogeneous plant-based fibers and other ill-defined bioactive contents. Therefore, we experimentally used formula to delay weaning and fed standard purified diets in adult mice. Because breast milk differs from chow in metabolic composition, we included a ketogenic purified diet, which is metabolically similar to breast or formula milk, i.e. higher fat content. Our comparisons were designed to reflect relevant nutritional contrasts (e.g., fiber content and metabolic composition), without aiming to pinpoint individual dietary components as causal agents—a task that would require substantial additional experimentation. As the reviewer correctly points out, metabolic composition of the diet also influences cDC1 in the spleen, however, it does not affect CXCL9 production, which is in line with our interpretation on page 13-14 of the revised manuscript. It will be interesting to further define the exact mechanisms by which metabolic composition and fiber context influence DC homeostasis.

Reviewer #2 (Remarks to the Author):

To the best of my appreciation, the authors have satisfactorily answered most of the points and concerns from all reviewers.

They have performed many new bench experiments, with the generation of additional novel high-quality data complementing the initial results present in the first version of the manuscript.

They have also edited some of their interpretations or conclusions, to make them more precise or to nuance them, accordingly to the recommendations from the reviewers.

Altogether, these changes have further improved the quality of the manuscript, in a manner that convincingly demonstrates the originality and potential importance of the discovery of the specific activation state imprinted on splenic cDC1s by the diet change at the time of weaning of the mice.

I have only identified a minor point left, which pertains to the functions of the specific activation state of cDC1s identified by the authors.

To the best of my understanding, the authors have at least partly changed their hypothesis between the initial manuscript and its revised version from (i) an immunogenic cDC1 activation state poised for promoting protective responses to infectious agents, to (ii) a possible role in tolerance induction to microbial or food antigens. Yet, this is not entirely clear and I am confused by the discussion on this issue.

In particular, the specific hypothesis is not clear to me behind the sentence “our data suggest that the immune system systemically relays dietary cues during weaning, allowing it to tailor the T cell response against environmental antigens to the need of the developing organism, striking a balance between effector immunity and T cell tolerance”.

On the one hand, the authors state that the cDC1 activation state they identify is immunogenic. “cDC intrinsic loss of Stat1 reduces cytotoxic CD8+ TEM cells in spleen, suggesting that the homeostatic maturation of cDC1 is not exclusively tolerogenic”, “our study identifies a previously unrecognized, microbiota-independent regulatory circuit by which dietary cues are relayed through IFNγ to imprint spleen-resident cDC1 with an immunogenic

maturation program" and "these findings redefine the landscape of steady-state cDC1 maturation, challenging the prevailing view that homeostatic cDC1 maturation primarily promotes tolerance".

On the other hand, they write that the CD8+ T cell response promoted by these cDC1s is associated to tolerance to food or microbial antigens. "Steady state CD49d+CD8+ TEM cells likely recognize environmental or food-derived antigens. […] Antigens encountered via the diet normally induce T cell tolerance, although such tolerance mechanisms can be suspended depending on the context in which dietary antigens are sampled. […] food-antigen specific CD8+ T cells were more cytotoxic in spleens of weanling mice on chow compared to milk diet. Effective desensitization against food allergens by oral immunotherapy has been linked to an increased frequency of cytotoxic CD8+ TEM cells, specifically in peripheral blood of patients with sustained unresponsiveness over those with recurring".

I do not understand what hypothesis the authors privilege in the end about the function of the cDC1 activation state imprinted on splenic cDC1s by the diet change at the time of weaning of the mice. Is-it contributing to tolerance to food or microbial antigens, or poised to promote protective responses to infectious agents?

I understand that addressing it experimentally is beyond the scope of the present study and not necessary for publication, considering the originality and mechanistic dissection of the reported observation.

However, I think that, in the discussion of the manuscript, the perspectives open from the current work require further clarifications, including regarding the hypotheses proposed on the function of the cDC1 activation state discovered and on how to test them experimentally in future studies.
In this regard, the authors should cite and discuss the following preprint about the role of cDC1s in intestinal tolerance (This S … Paidassi H. Type 1 and type 2 dendritic cell subsets cooperate to maintain intestinal immune tolerance via integrin αvβ8-mediated TGF-β activation. bioRxiv 2026. doi: https://doi.org/10.64898/2026.02.26.707908).

We thank the reviewer for this thoughtful assessment and the encouraging comments. We agree that our previous discussion did not clearly distinguish between our experimental findings and the hypotheses arising from them.

We have therefore substantially revised the discussion to clarify our interpretation. Our data demonstrate that dietary context systemically tunes the effector state of food antigen–specific $CD8^+$ T cells through IFNγ-dependent maturation of splenic cDC1. However, we do not conclude from our data whether this heightened effector state ultimately promotes host protection, tissue surveillance, or mechanisms underlying oral tolerance. Instead, we now present these as alternative, non-mutually exclusive hypotheses for future investigation.

To address the reviewer's comment, we have expanded the discussion (pages 17–18) to propose a model in which food antigen–specific $CD8^+$ T cells are primed in gut-associated lymphoid tissues and subsequently interact with $CXCL9^+$ cDC1 after recirculating through the spleen, where cDC1 may further shape their functional state. We also discuss experimental approaches that could distinguish between the possible physiological roles of this regulatory circuit, including infection and allergy models.

Finally, we have incorporated and discussed the recommended preprint describing the role of cDC1 in intestinal immune tolerance.
