# Supplementary material for: Peri-weaning, diet-induced activation of an IFNγ-mediated regulatory circuit promotes cDC1 maturation and CD8+ T cell differentiation
Source: Nat Commun. 2026 Aug 4;17:7631. doi: 10.1038/s41467-026-75853-5 (PMC13439377; doi:10.1038/s41467-026-75853-5)
Supplement: Supplementary file 3 [file 41467_2026_75853_MOESM1_ESM.pdf]

Supplementary information

Peri-weaning, diet-induced activation of an IFNγ-mediated regulatory circuit promotes cDC1 maturation and $CD8^{+}$ T cell differentiation

First authors: Doğuş Altunöz and Ramin Shakiba
Corresponding author: Barbara U. Schraml

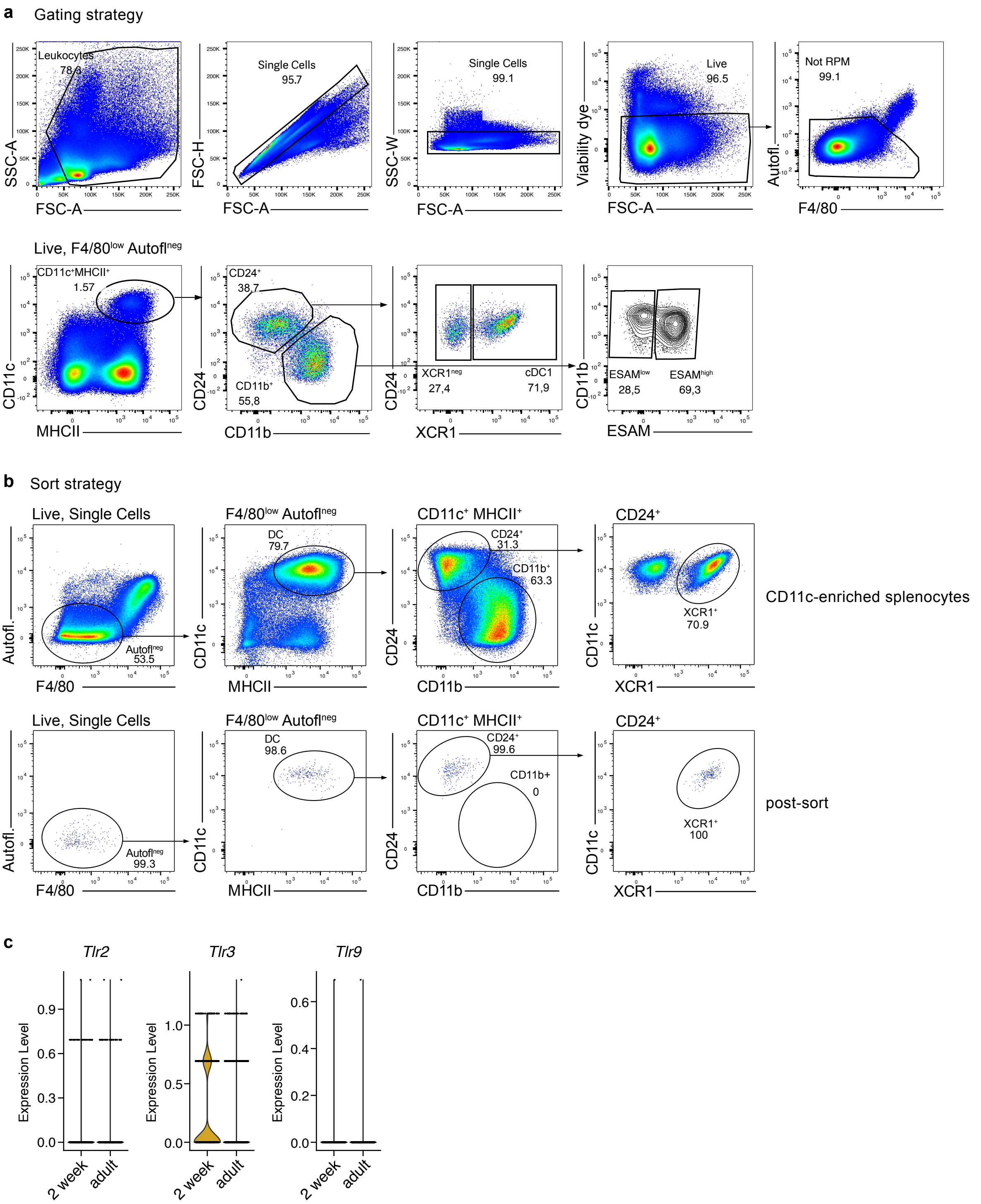

Supplemental Figure 1: Representative sort strategy and purity of splenic cDC1

a, Representative gating strategy for splenic DC subsets from a 9-week-old mouse. Live single cells were gated and F4/80^{high} autofluorescent (Autofl) red pulp macrophages (RPMs) excluded. CD11c^{+}MHCII^{+} DCs were then divided into CD24^{+}XCR1^{+} cDC1, CD24^{+}XCR1^{neg} tDC and CD24^{neg}CD11b^{+} cDC2. cDC2 were further divided into ESAM^{high} and ESAM^{low} cDC2. **b,** Representative strategy from a 10-week-old mouse to sort XCR1^{+} cDC1 from CD11c-enriched splenocytes. Within live single leukocytes autofluorescent F4/80^{high} RPMs were excluded and within CD11c^{+}MHCII^{+} cells CD24^{+}CD11b^{neg}XCR1^{+} cDC1 were gated. Top panel pre-sort, bottom panel sort purity. **c**, Expression of *Tlr2*, *Tlr3* and *Tlr9* in cDC1 from 2-week-old and adult mice in the scRNA/ATAC-seq data from Fig. 1e-f.

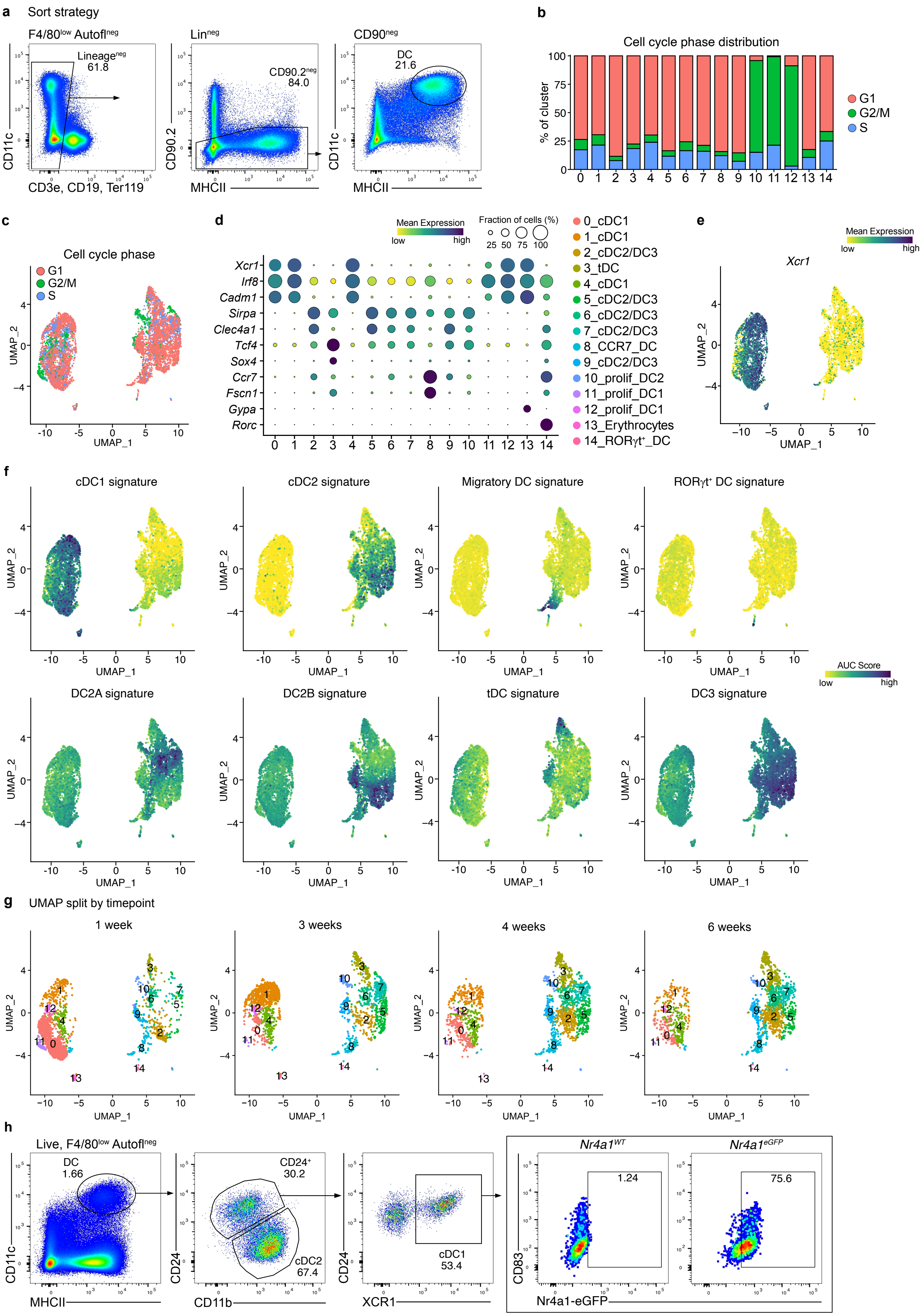

a
Sort strategy
F4/80low Autoflneg
Lineageneg 61.8
CD11c
CD3e, CD19, Ter119
Linneg
CD90.2neg 84.0
CD90.2
MHCII
CD90neg
DC 21.6
CD11c
MHCII
b
Cell cycle phase distribution
% of cluster
G1
G2/M
S
c
Cell cycle phase
G1
G2/M
S
UMAP_2
UMAP_1
d
Mean Expression
low
high
Fraction of cells (%)
25 50 75 100
Xcr1
Irf8
Cadm1
Sirpa
Clec4a1
Tcf4
Sox4
Ccr7
Fscn1
Gypa
Rorc
0_cDC1
1_cDC1
2_cDC2/DC3
3_tDC
4_cDC1
5_cDC2/DC3
6_cDC2/DC3
7_cDC2/DC3
8_CCR7_DC
9_cDC2/DC3
10_prolif_DC2
11_prolif_DC1
12_prolif_DC1
13_Erythrocytes
14_RORγt+_DC
e
Mean Expression
low
high
Xcr1
UMAP_2
UMAP_1
f
cDC1 signature
cDC2 signature
Migratory DC signature
RORγt+ DC signature
DC2A signature
DC2B signature
tDC signature
DC3 signature
AUC Score
low
high
UMAP_2
UMAP_1
g
UMAP split by timepoint
1 week
3 weeks
4 weeks
6 weeks
UMAP_2
UMAP_1
h
Live, F4/80low Autoflneg
DC 1.66
CD11c
MHCII
CD24+ 30.2
cDC2 67.4
CD24
CD11b
cDC1 53.4
CD24
XCR1
Nr4a1WT
1.24
Nr4a1eGFP
75.6
CD83
Nr4a1-eGFP

Supplemental Figure 2: scRNA-seq analysis of dendritic cells across age

a-g, CD11c$^+$MHCII$^+$ cells were sorted from spleens of mice at 1, 3, 4 and 6 weeks of age for scRNA-seq. **a,** Representative sort strategy from a 6-week-old mouse. CD19, CD3e and Ter119-negative cells were enriched using anti-FITC magnetic beads. From live leukocytes, F4/80^{low}Autofluorescence (Autofl)-negative cells were gated and remaining FITC-positive and CD90.2-positive cells were excluded before sorting CD11c$^+$MHCII$^+$ cells. **b, c,** Distribution of cell cycle phase across clusters from UMAP in Fig. 2b. **d,** Bubble plot of selected genes used for cell type identification. **e,** Expression levels of *Xcr1* on the UMAP display. **f,** UMAP display of AUC scores for the indicated gene signatures used for cluster identification. **g,** Cells for the individual time points were separately projected onto UMAPs. **h,** Representative gating strategy for the identification of cDC1 in spleen. Live, autofluorescence-negative cells from 7-week-old *Nr4a1*^{eGFP} and wild type mice were gated, followed by identification of CD24$^+$XCR1$^+$ cells as shown. GFP-positive cDC1 were identified based on a wild type control.

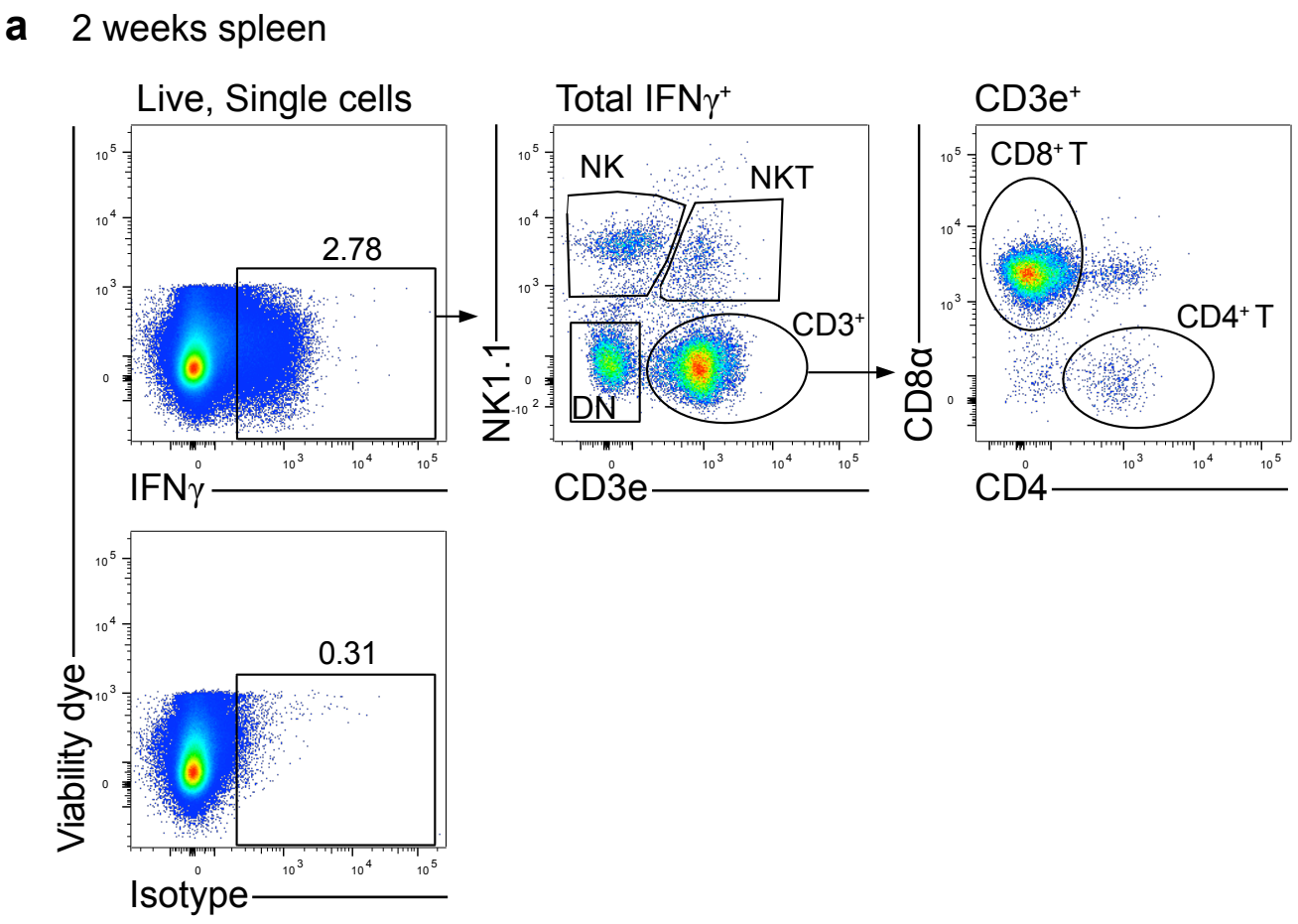

Supplemental Figure 3: Flow cytometry of IFNγ production by splenocytes

a, Splenocytes from mice of different ages were treated with PMA/Ionomycin for 5 hours, Brefeldin A was added for the last 3 hours. Then intracellular cytokine staining was performed. Within live leukocytes, IFNγ^{+} cells were gated based on an isotype-matched control antibody (bottom panel) and subdivided into NK cells (NK1.1^{+}CD3e^{neg}), NKT cells (NK1.1^{+}CD3e^{+}), T cells (NK1.1^{neg}CD3e^{+}) and NK1.1^{neg}CD3e^{neg} cells (DN: double negative cells). T cells were further divided into CD8^{+} and CD4^{+} T cells. Representative gating strategy from a 2-week-old mouse is shown.

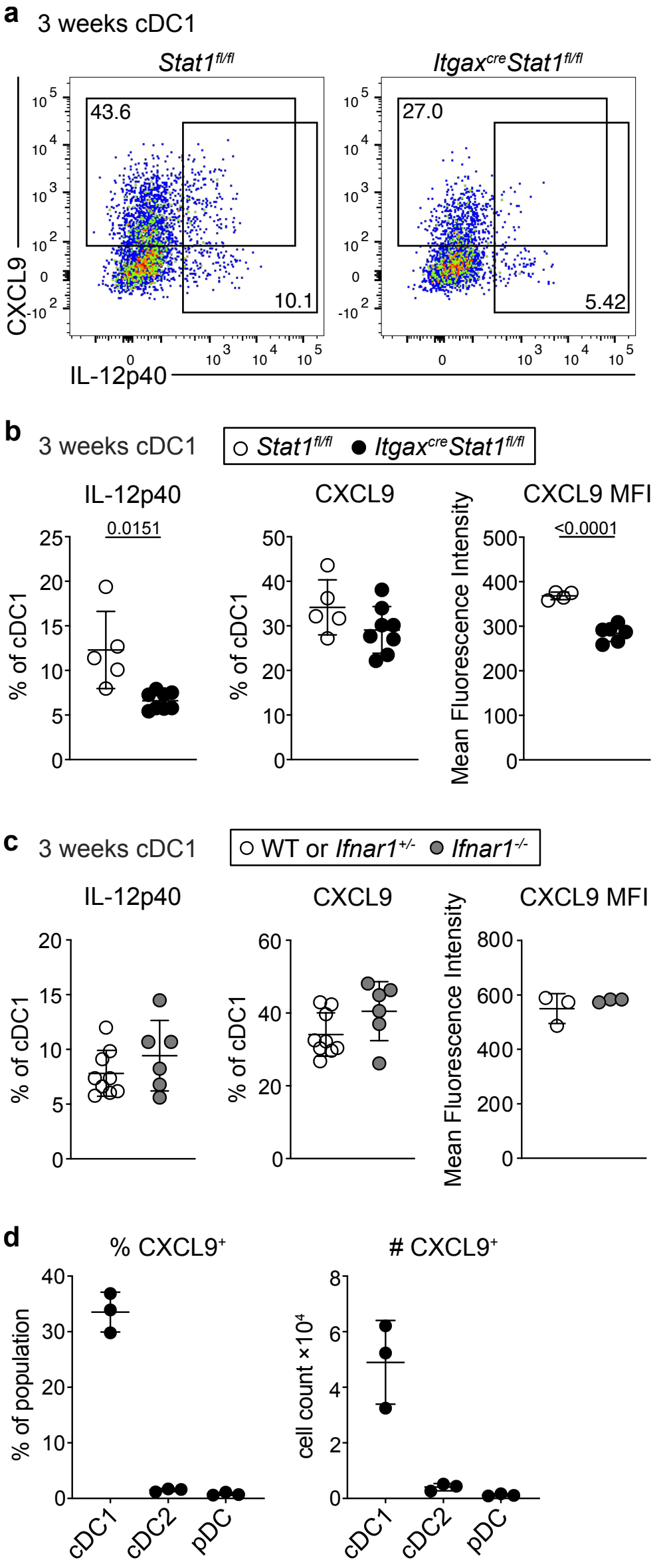

Supplemental Figure 4: CXCL9 production from cDC1 at 3-weeks-old is *Ifnar1*-independent

a-d Splenocytes from 3-week-old mice of the indicated genotypes were cultured for 4 hours with Brefeldin A and Monensin. XCR1^{+} cDC1 were then analyzed for cytokine expression by flow cytometry. **a,** Representative gating. **b,** Quantification of IL-12p40^{+} and CXCL9^{+} cDC1 from 3-week-old *Itgax^{cre}Stat1$^{fl/fl}$* (*n*=8; for MFI *n*=6) and *Stat1$^{fl/fl}$* (*n*=5; for MFI *n*=4) littermates. **c**, Frequency of IL-12p40^{+} and CXCL9^{+} cDC1 from 3-week-old and *Ifnar1$^{+/-}$* or wild type (WT) (*n*=9; for MFI *n*=3) or *Ifnar1$^{-/-}$* (*n*=6; for MFI *n*=3) mice. Each dot represents one biological replicate pooled from three independent experiments. **d**, CXCL9 production in cDC1, cDC2 and pDC (CD19^{neg}CD11c^{+}SiglecH^{+}B220^{+}) from 3-week-old wild type mice after 4 hours of culture with Brefeldin A and Monensin (*n*=3 biological replicates from one experiment). Horizontal bars represent mean, error bars represent SD. **b, c,** Statistical analysis was performed using two-tailed Welch's *t*-test. *p*-values for statistically significant comparisons are shown. Source data are provided as a Source Data file.

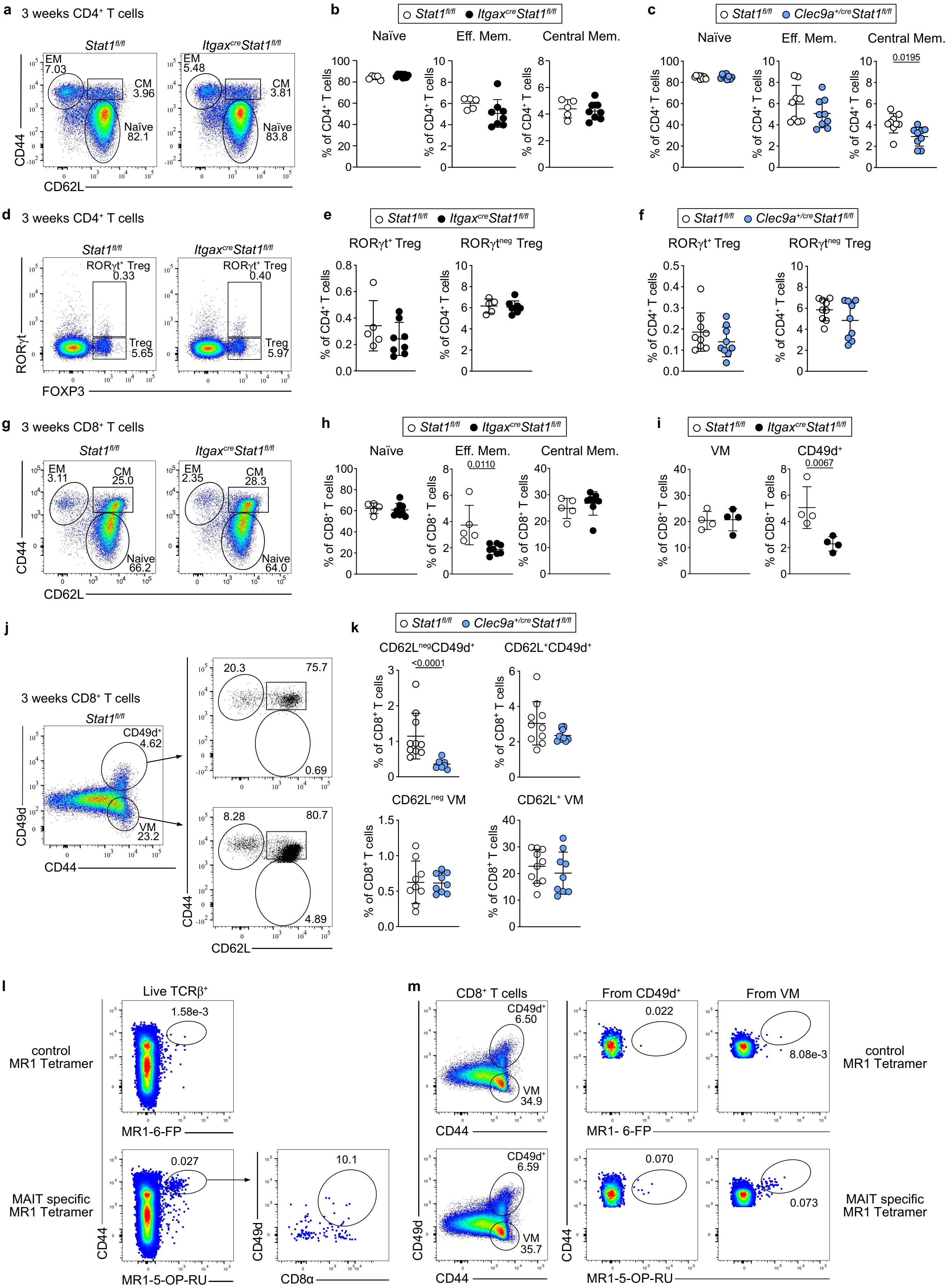
a
3 weeks CD4+ T cells
Stat1fl/fl
ItgaxcreStat1fl/fl
EM 7.03
CM 3.96
Naïve 82.1
EM 5.48
CM 3.81
Naïve 83.8
CD44
CD62L
b
Stat1fl/fl
ItgaxcreStat1fl/fl
Naïve
Eff. Mem.
Central Mem.
% of CD4+ T cells
c
Stat1fl/fl
Clec9a+/creStat1fl/fl
Naïve
Eff. Mem.
Central Mem.
0.0195
% of CD4+ T cells
d
3 weeks CD4+ T cells
Stat1fl/fl
ItgaxcreStat1fl/fl
RORγt+ Treg 0.33
Treg 5.65
RORγt+ Treg 0.40
Treg 5.97
RORγt
FOXP3
e
Stat1fl/fl
ItgaxcreStat1fl/fl
RORγt+ Treg
RORγtneg Treg
% of CD4+ T cells
f
Stat1fl/fl
Clec9a+/creStat1fl/fl
RORγt+ Treg
RORγtneg Treg
% of CD4+ T cells
g
3 weeks CD8+ T cells
Stat1fl/fl
ItgaxcreStat1fl/fl
EM 3.11
CM 25.0
Naive 66.2
EM 2.35
CM 28.3
Naive 64.0
CD44
CD62L
h
Stat1fl/fl
ItgaxcreStat1fl/fl
Naïve
Eff. Mem.
Central Mem.
0.0110
% of CD8+ T cells
i
Stat1fl/fl
ItgaxcreStat1fl/fl
VM
CD49d+
0.0067
% of CD8+ T cells
j
3 weeks CD8+ T cells
Stat1fl/fl
CD49d+ 4.62
VM 23.2
CD49d
CD44
20.3
75.7
0.69
8.28
80.7
4.89
CD44
CD62L
k
Stat1fl/fl
Clec9a+/creStat1fl/fl
CD62LnegCD49d+
<0.0001
CD62L+CD49d+
CD62Lneg VM
CD62L+ VM
% of CD8+ T cells
l
Live TCRβ+
1.58e-3
control MR1 Tetramer
MR1-6-FP
0.027
10.1
MAIT specific MR1 Tetramer
CD44
MR1-5-OP-RU
CD49d
CD8α
m
CD8+ T cells
CD49d+ 6.50
VM 34.9
CD44
From CD49d+
From VM
0.022
8.08e-3
control MR1 Tetramer
MR1- 6-FP
CD49d+ 6.59
VM 35.7
CD49d
CD44
0.070
0.073
MAIT specific MR1 Tetramer
CD44
MR1-5-OP-RU

Supplemental Figure 5: Analysis of spleen T cells in 3-week-old mice

a,b, Representative gating strategy **(a)** and quantification of **(b)** effector memory ($CD44^{+}CD62L^{neg}$), central memory ($CD44^{+}CD62L^{+}$) and naïve ($CD44^{neg}CD62L^{+}$) $CD4^{+}$ T cells in spleens of 3-week-old *Itgax^{cre}Stat1$^{fl/fl}$* (*n*=8) and *Stat1$^{fl/fl}$* (*n*=5) littermates. **c,** Quantification of the indicated populations in 3-week-old *Clec9a$^{+/cre}$Stat1$^{fl/fl}$* and *Stat1$^{fl/fl}$* littermates (*n*=9). **d,** Representative gating strategy for the identification of $Foxp3^{+}$ and $Foxp3^{+}ROR\gamma t^{+}$ Tregs. **e,** Quantification of $CD4^{+}$ Treg populations in 3-week-old *Itgax^{cre}Stat1$^{fl/fl}$* (*n*=8) and *Stat1$^{fl/fl}$* (*n*=5) littermates. **f,** Quantification of $CD4^{+}$ Treg populations in 3-week-old *Clec9a^{cre}Stat1$^{fl/fl}$* and *Stat1$^{fl/fl}$* littermates (*n*=9). **g-h,** $CD8^{+}$ T cells in the spleen were divided into effector memory ($CD44^{+}CD62L^{neg}$), central memory ($CD44^{+}CD62L^{+}$) and naïve ($CD44^{neg}CD62L^{+}$) populations. Representative gating (**g**) and quantification (**h**) of these populations in spleens of 3-week-old *Itgax^{cre}Stat1$^{fl/fl}$* (*n*=8) and *Stat1$^{fl/fl}$* (*n*=5) littermates. **i**, Quantification of splenic $CD49d^{+}$ and virtual memory (VM) $CD8^{+}$ T cell populations in 3-week-old *Itgax^{cre}Stat1$^{fl/fl}$* and *Stat1$^{fl/fl}$* littermates (*n*=4). **j,** Splenocytes from 3-week-old *Clec9a$^{+/cre}$Stat1$^{fl/fl}$* (*n*=9) and *Stat1$^{fl/fl}$* (*n*=10) mice were isolated and $CD8^{+}$ T cells were gated as $CD49d^{+}CD44^{+}$ and $CD49d^{neg}CD44^{+}$ VM. Within these populations, cells were divided into central memory ($CD44^{+}CD62L^{+}$) and effector memory ($CD44^{+}CD62L^{neg}$) cells. **k,** Subsets gated in **(j)** were quantified as percentage of total $CD8^{+}$ T cells. **l,** MAIT cells were identified in spleens of 3-week-old wild type mice as $TCR\beta^{+}$ MR1-5-OP-RU^{+} cells as shown and analyzed for CD8α and CD49d expression. 6-FP loaded MR1 tetramer was used as control. **m,** MR1-5-OP-RU signal within $CD44^{+}CD49d^{+}$ or $CD44^{+}CD49d^{neg}$ $CD8^{+}$ T cells is shown. Each dot represents one biological replicate pooled from two (**c, f, i, k**) or three (**b, e, h**) independent experiments, horizontal bars represent mean, error bars represent SD. Statistical analysis was performed and *p*-values were determined using two-tailed Welch's *t*-test. Only statistically significant comparisons are indicated. Source data are provided as a Source Data file.

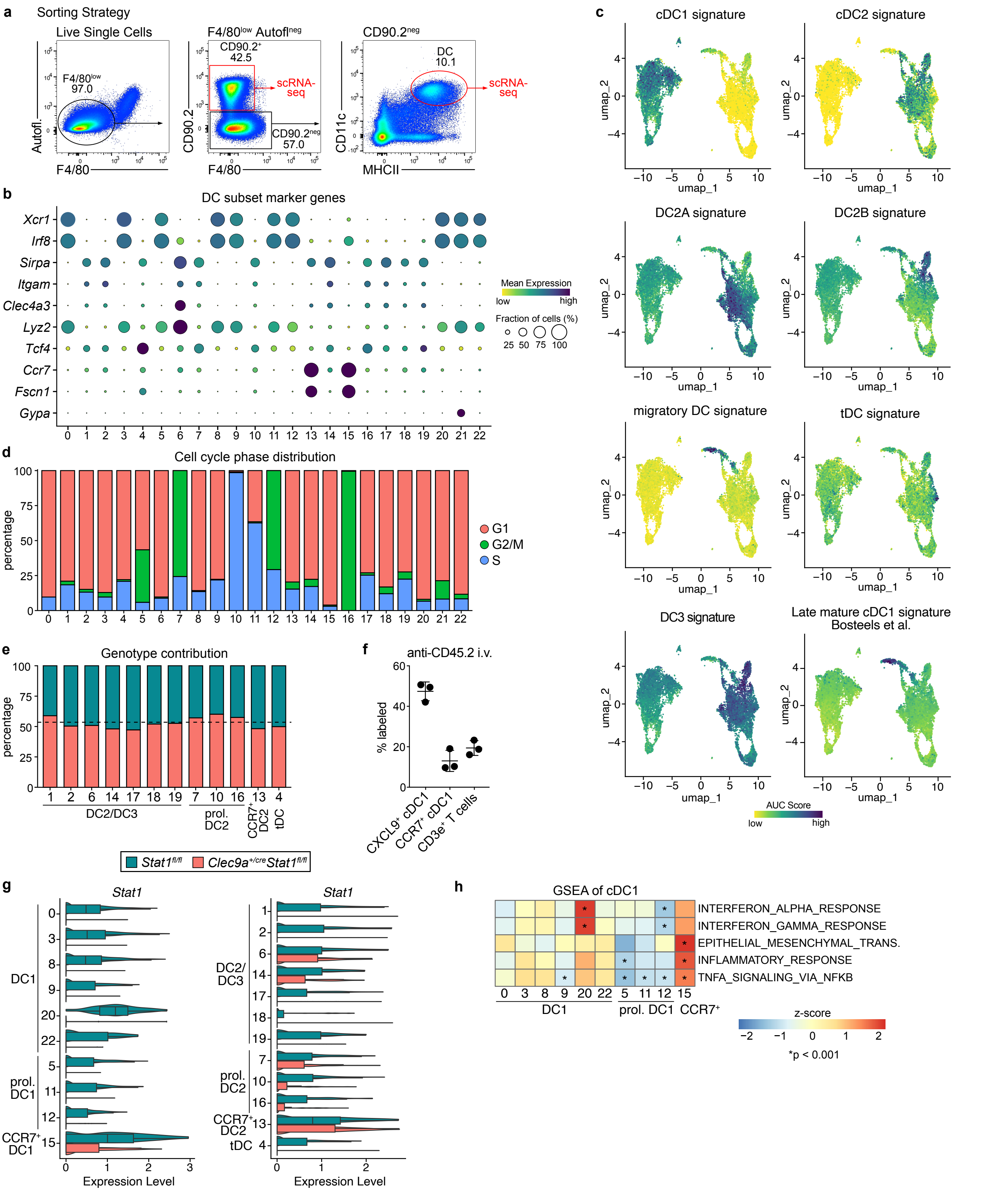

Supplemental Figure 6: scRNA-seq of spleen cDC from 3-week-old *Clec9a^{cre}Stat1$^{fl/fl}$* mice

a, Sort strategy for splenic CD90.2^{+} cells and CD11c^{+}MHCII^{+} cells from the spleens of 3-week-old *Clec9a$^{+/cre}$Stat1$^{fl/fl}$* and *Stat1$^{fl/fl}$* littermates for scRNA-seq in Fig. 6 and 7. Live leukocytes were gated and RPMs were excluded as F4/80^{high} Autofluorescence (Autofl)^{high} cells. Then CD90.2^{+} cells and CD90.2^{neg}CD11c^{+}MHCII^{+} cells were sorted. **b-e, g, h,** scRNA-seq of CD11c^{+}MHCII^{+} cells. Clusters correspond to the UMAP in Fig. 6a. **b,**

Bubble plot of selected genes used for cell type identification. **c,** UMAP display of AUC scores for the indicated gene signatures used for cluster identification. **d,** Distribution of cell cycle phase across clusters. **e,** Contribution of cells from each genotype to the indicated clusters as percentage. Dotted line represents fraction of cells from *Clec9a*$^{+/cre}$*Stat1*$^{fl/fl}$ mice as percentage of total cDC2/DC3/tDC in the dataset. **f**, 3-week-old mice were injected intravenously with anti-CD45.2 antibody. Two minutes later the indicated splenic populations were analyzed for CD45.2 labeling (n=3). Each dot represents one biological replicate from one experiment, horizontal bars represent mean, error bars represent SD. **g,** Expression of *Stat1* in cDC1 and cDC2 clusters split by genotype. Box represents interquartile range, horizontal bar represents median, whiskers represent minimum and maximum enrichment scores. **h,** Gene set enrichment scores of the indicated gene sets that were significantly enriched in cDC1 clusters 20 or 15 are shown. Statistical analysis was performed and p-values were determined using two-tailed variance-inflated Wilcoxon rank sum test with adjustment for multiple comparisons via the FDR method, *$p < 0.001$. Source data are provided as a Source Data file.

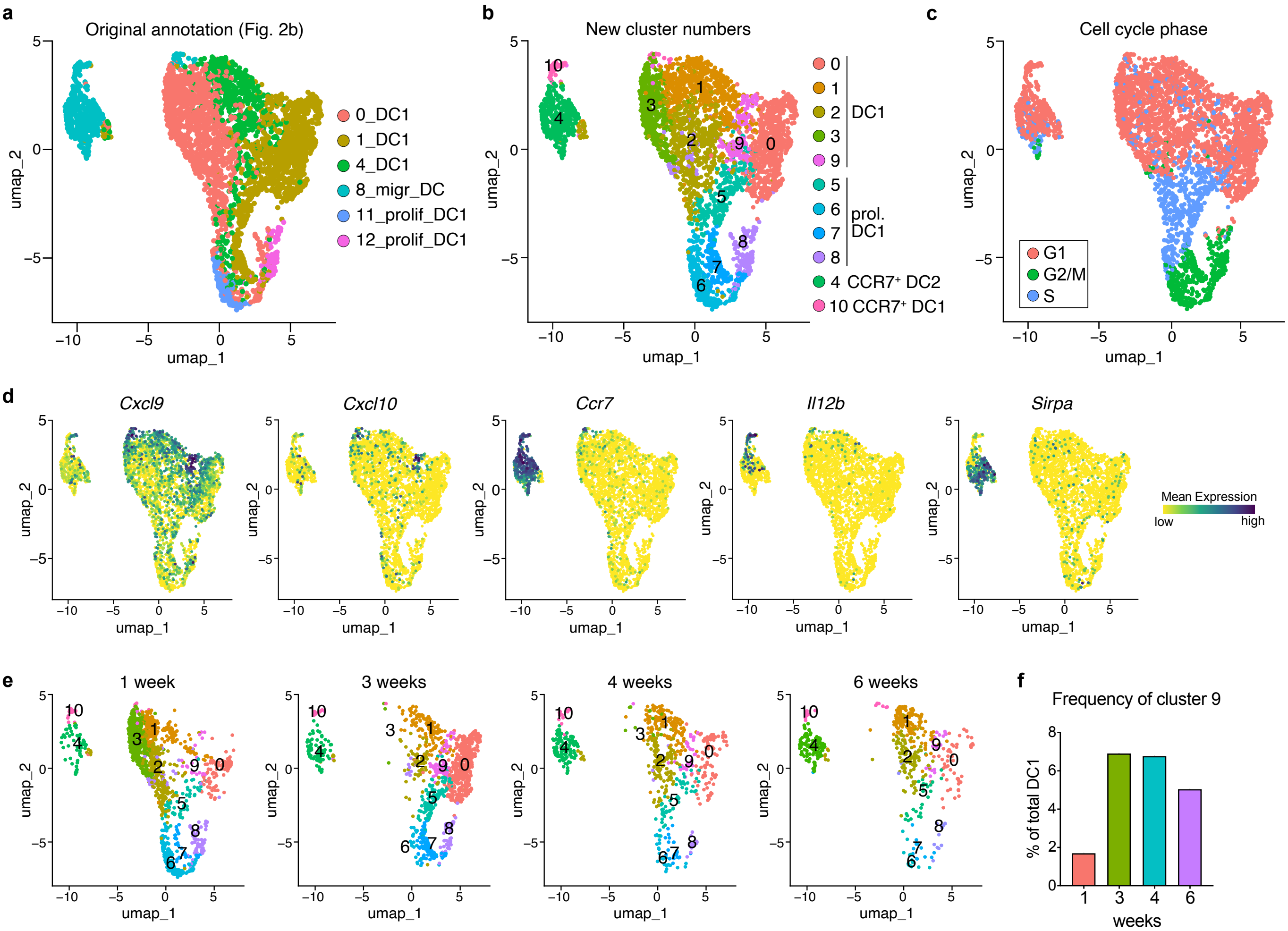

Supplemental Figure 7: Refined analysis of isolated cDC1 and CCR7^{+} cDC clusters from the scRNA-seq dataset across age shown in Fig. 2

a-f, cDC1 and CCR7^{+} cDC clusters from the scRNA-seq dataset across age (Fig. 2b) were isolated and reclustered. **a,** UMAP display of isolated cells showing their original annotation as in Fig. 2b. **b,** UMAP showing new cluster annotations after refined unsupervised clustering. **c,** UMAP displaying cell cycle phase. **d,** Expression of *Cxcl9, Cxcl10, Ccr7, Il12b,* and *Sirpa* projected on the UMAP display. **e,** UMAP split by time point. **f,** Frequency of cluster 9 as percentage of total DC1 across different time points.

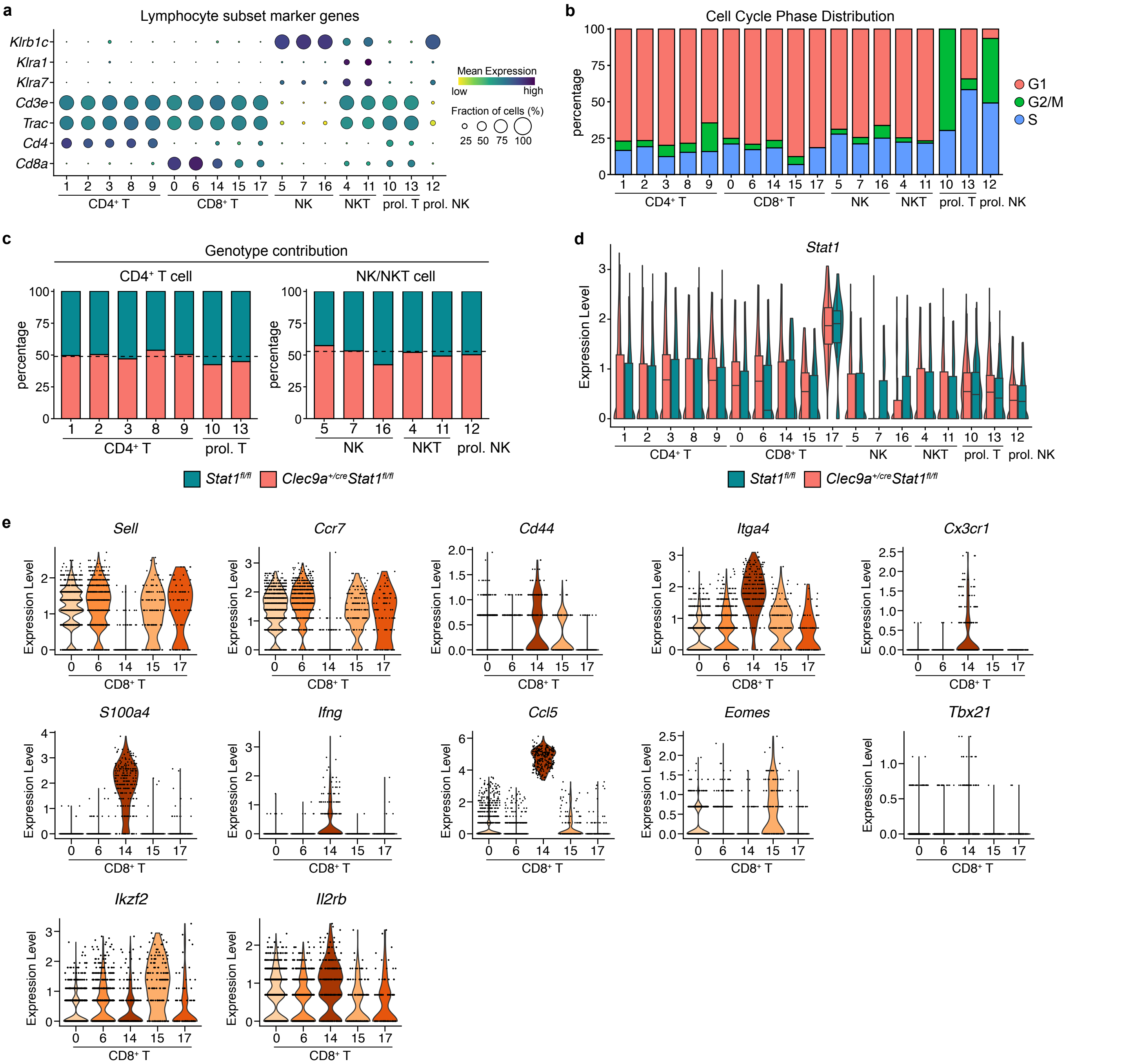

Supplemental Figure 8: scRNA-seq analysis of spleen CD90.2^{+} cells from 3-week-old *Clec9a^{cre}Stat1$^{fl/fl}$* mice
a-e, scRNA-seq analysis of CD90^{+} cells. Clusters correspond to the UMAP in Fig. 7a. **a,** Bubble plot of selected genes used for cluster identification. **b,** Distribution of cell cycle phase across clusters. **c,** Contribution of cells from each genotype to CD4^{+} T cell and NK/NKT cell clusters. Dotted line represents fraction of cells from *Clec9a$^{+/cre}$Stat1$^{fl/fl}$* mice as a percentage of total T cell or NK/NKT in the dataset. **d,** Box plot showing expression of *Stat1* in the indicated clusters split by genotype. Box represents interquartile range, horizontal bar represents median, whiskers represent minimum and maximum enrichment scores. **e,** Expression of the indicated genes used for cluster identification across CD8^{+} T cell clusters.

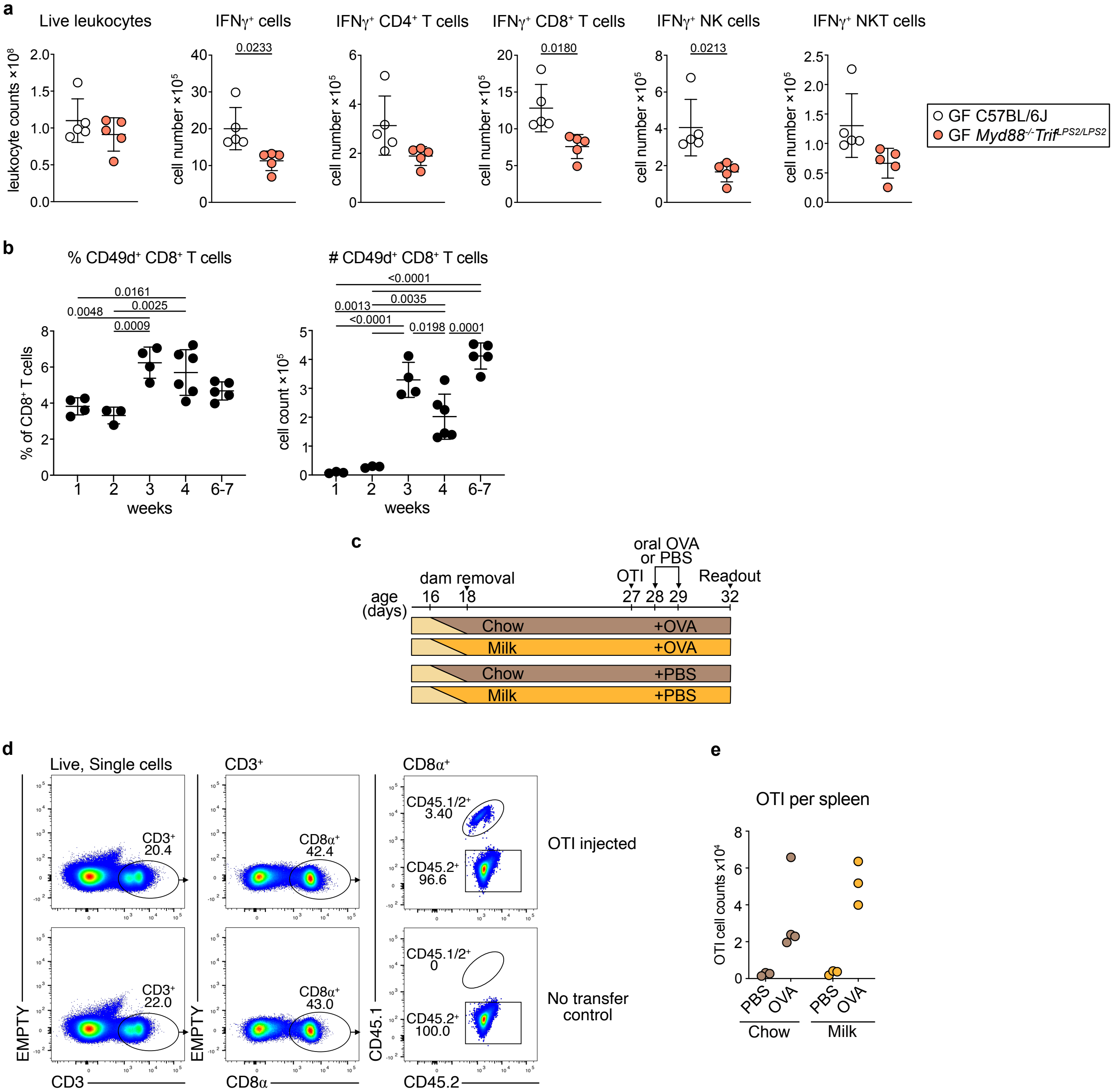

Supplemental Figure 9: Lymphocytes from germ-free *Myd88$^{-/-}$Trif$^{LPS2/LPS2}$* mice show reduced IFNγ production and specificity analyses of the food antigen response

a, Spleen cellularity, total IFNγ-producing cells and the indicated IFNγ-producing lymphocyte subsets were quantified from spleens of 3-week-old germ-free (GF) *Myd88$^{-/-}$Trif$^{LPS2/LPS2}$* or age-matched GF wild type controls (*n*=5 from one experiment). **b,** CD49d^{+} cells as percentage of total CD8^{+} T cells and numbers of CD49d^{+} CD8^{+} T cells in spleens from wild type SPF mice at the indicated ages (1 or 3 weeks (*n*=4); 2 weeks (*n*=3); 4 weeks (*n*=6); 6-7 weeks (*n*=5)). **c-e,** SPF mice were either conventionally weaned on day 18 or weaning was delayed by placing mice on formula milk from day 16 after birth while restricting access to chow. At 27 days of age mice received 1×10^{5} naïve OTI cell intravenously. On the next two consecutive days mice were gavaged with either OVA or PBS and three days later spleens were analyzed. **c,** Experimental scheme. **d,** Representative gating to distinguish CD45.1/2^{+}OTI cells from endogenous CD45.2^{+} CD8^{+} T cells. **e,** Number of OTI cells in the spleen (PBS groups or milk OVA (*n*=3); chow OVA (*n*=4)). Each dot represents one biological replicate pooled from three independent experiments (**b**) or representative of two independent experiments **(e)**. Horizontal bars represent mean, error bars represent SD. Statistical analysis was performed and *p*-values were determined using two-tailed Welch's *t*-test (**a**) or one-way ANOVA with two-tailed Tukey's multiple comparisons test (**b**). Only statistically significant comparisons are indicated. Source data are provided as a Source Data file.
