# Supplementary material for: Peri-weaning, diet-induced activation of an IFNγ-mediated regulatory circuit promotes cDC1 maturation and CD8+ T cell differentiation
Source: Nat Commun. 2026 Aug 4;17:7631. doi: 10.1038/s41467-026-75853-5 (PMC13439377; doi:10.1038/s41467-026-75853-5)
Supplement: Supplementary file 4 [file 41467_2026_75853_MOESM2_ESM.pdf]

Description of Additional Supplementary Files

Supplementary Data 1. Published gene signatures for indicated DC subsets, or states with source publication

Supplementary Data 2. Differentially expressed genes expressed higher in cDC1 cluster 0 vs cDC1 cluster 1

Supplementary Data 3. Differentially expressed genes expressed higher in cDC1 cluster 0 vs cDC1 cluster 4

Supplementary Data 4. Differentially expressed genes expressed higher in cDC1 cluster 1 vs cDC1 cluster 0

Supplementary Data 5. Differentially expressed genes expressed higher in cDC1 cluster 1 vs cDC1 cluster 4

Supplementary Data 6. Differentially expressed genes expressed higher in cDC1 cluster 4 vs cDC1 cluster 0

Supplementary Data 7. Differentially expressed genes expressed higher in cDC1 cluster 4 vs cDC1 cluster 1

Supplementary Data 8. Marker genes between cDC1 clusters from Clec9a-cre Stat1-flox and Stat1-flox mice generated via Seurat's FindAllMarkers() function

Supplementary Data 9. Top 15 Marker genes between CD90+ clusters from Clec9a-cre Stat1-flox and Stat1-flox mice generated via Seurat's FindAllMarkers() function

Supplementary Data 10. Antibody-fluorophore conjugates used in this study

Supplementary Data 11. Primer sequences used in this study

Supplementary Data 12. Comparison of standard vs ketogenic diets used in this study
